# Multi-Target Neuroprotective Compound Exhibits EAAT2-Modulating and Alzheimer’s Pathology–Attenuating Effects in In Vitro and In Vivo Models

**DOI:** 10.1021/acschemneuro.5c00873

**Published:** 2026-04-29

**Authors:** Ahmet Hacımüftüoğlu, Nurullah Saraçoğlu, Sana Saffour, Nadeem Abad, Yunus Kesgun, Nadjiba Zegheb, Ersin Gundeger, Fatma Yeşilyurt, Merve Nur Ataş, Gizem Bati-Ayaz, Öznur Altunlu, Burak Çınar, Mehmet Ali Yörük, Ufuk Okkay, Mustafa Özkaraca, Orhan Ateş, Ali Taghizadehghalehjoughi, Ferruh Lafzi, Hasan Türkez

**Affiliations:** † Department of Medical Pharmacology, Faculty of Medicine, 37503 Atatürk University, 25240 Erzurum, Turkey; ‡ Department of Chemistry, Faculty of Sciences, Atatürk University, 25240 Erzurum, Turkey; § Biotechnology Institute, 37504 Ankara University, 06135 Ankara, Turkey; ∥ Trustlife Laboratories Drug Research & Development Center, 34774 İstanbul, Turkey; ⊥ Biochemistry Department, Faculty of Pharmacy, 578738 Istanbul Health and Technology University, Sütlüce, Beyoğlu, 34275 İstanbul, Turkey; # Faculty of Veterinary Medicine, Department of Pathology, Cumhuriyet University, 58140 Sivas, Turkey; ¶ Department of Eye Diseases, Faculty of Medicine, Atatürk University, 25240 Erzurum, Turkey; ∇ Department of Medical Pharmacology, Faculty of Medicine, 121945 Bilecik Seyh Edebali University, 11230 Bilecik, Turkey; ○ Department of Medical Biology, Faculty of Medicine, Atatürk University, 25240 Erzurum, Turkey

**Keywords:** neurodegeneration, glutamate excitotoxicity, Tau pathology, β-Amyloid pathology, oxidative stress

## Abstract

Alzheimer’s disease (AD) is a debilitating neurodegenerative disorder characterized by cognitive decline and memory loss. Current treatments offer limited efficacy, necessitating the development of innovative multitarget therapeutic strategies. Here, we present *N* ^3^,*N* ^5^-bis­(2-(5-methoxy-1*H*-indol-3-yl)­ethyl)-2,6-dimethyl-4-(2-nitrophenyl)­pyridine-3,5-dicarboxamide (**HCM-01**), a novel compound developed to target multiple neurodegenerative pathways implicated in AD. In vitro assays included MTT-based cell viability analyses performed in two complementary experimental settings: primary neuronal cultures and astrocyte-based in vitro cell culture models exposed to glutamate. In primary hippocampal neuronal cultures, glutamate exposure induced a statistically significant reduction in cell viability compared with vehicle-treated controls, consistent with glutamate-induced excitotoxicity. Under these conditions, **HCM-01** treatment resulted in a statistically significant improvement in neuronal viability, showing a greater protective effect compared with donepezil and memantine. In contrast, in astrocyte-based in vitro cultures, the applied glutamate concentration did not induce overt cytotoxicity, in line with the intrinsic neuroprotective and glutamate-buffering role of astrocytes. Accordingly, astrocytic experiments were designed to assess functional modulation of glutamate-handling mechanisms rather than cell survival. Western blot analysis in C8-D1A astrocytic cells demonstrated increased expression of excitatory amino acid transporter 2 (EAAT2) following **HCM-01** treatment compared with control and reference drug-treated groups, suggesting modulation of astrocyte-mediated glutamate homeostasis. In parallel, redox analyses revealed that **HCM-01** improved oxidative/antioxidative balance, as evidenced by increased total antioxidant capacity (TAC) and reduced total oxidant status (TOS), supporting an indirect antioxidant contribution to its functional effects. In vivo behavioral assessment of **HCM-01** in a streptozotocin (STZ)-induced Alzheimer’s model in female Sprague–Dawley rats demonstrated that administration of **HCM-01** at doses of 50 mg/kg orally (oral, P.O. and intraperitoneal, I.P.) and 100 mg/kg (P.O.), significantly improved cognitive and memory functions in the passive avoidance (PA), Morris water maze (MWM), and locomotor activity tests. Moreover, histopathological and immunohistochemical analyses of different hippocampal regions revealed reduced neuronal damage, attenuation of tau pathology, antiamyloidogenic effect, and restoration of cholinergic function. Complementary in silico studies, including molecular docking, molecular dynamics simulations (MDS), and free energy calculations, suggested potential interactions of **HCM-01** with the allosteric site of EAAT2. Taken together, these findings suggest that **HCM-01** exerts neuroprotective effects against glutamate-induced excitotoxicity in primary hippocampal neurons while additionally modulating glutamatergic homeostasis and redox balance through functional mechanisms in astrocyte-based models, supporting its relevance as a multitarget preclinical candidate for early stage AD mechanisms.

## Graphical abstract

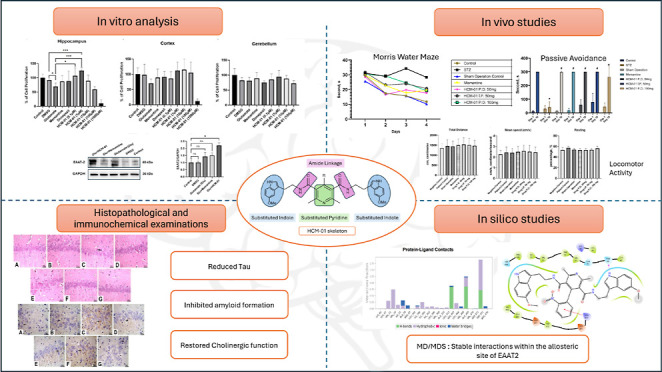

## Introduction

Alzheimer’s disease (AD) is the leading cause of dementia, accounting for 60–80% of all cases globally, with over 55 million individuals affected worldwide as of 2022. This progressive neurodegenerative disorder begins with mild cognitive impairment and advances to severe dementia, profoundly affecting daily activities, including basic functions such as swallowing and drinking.^1^ Despite its prevalence, the precise mechanisms underlying AD onset and progression remain elusive, with several competing hypotheses offering insights into the complex pathological alterations observed in the patients’ brains.^2^

One widely studied theory, the cholinergic hypothesis, implicates the deficiency of acetylcholine, a primary neurotransmitter, and cholinergic neuron dysfunction as critical contributors to AD-related learning deficits, memory loss, motor impairments, and sleep disturbances. Consequently, acetylcholinesterase (AChE) inhibitors like donepezil, rivastigmine, and galantamine have been developed, providing symptomatic relief for AD patients (Figure 1a).^3^ However, these therapies do not halt disease progression. (See Figure 2)

**Figure 1:**
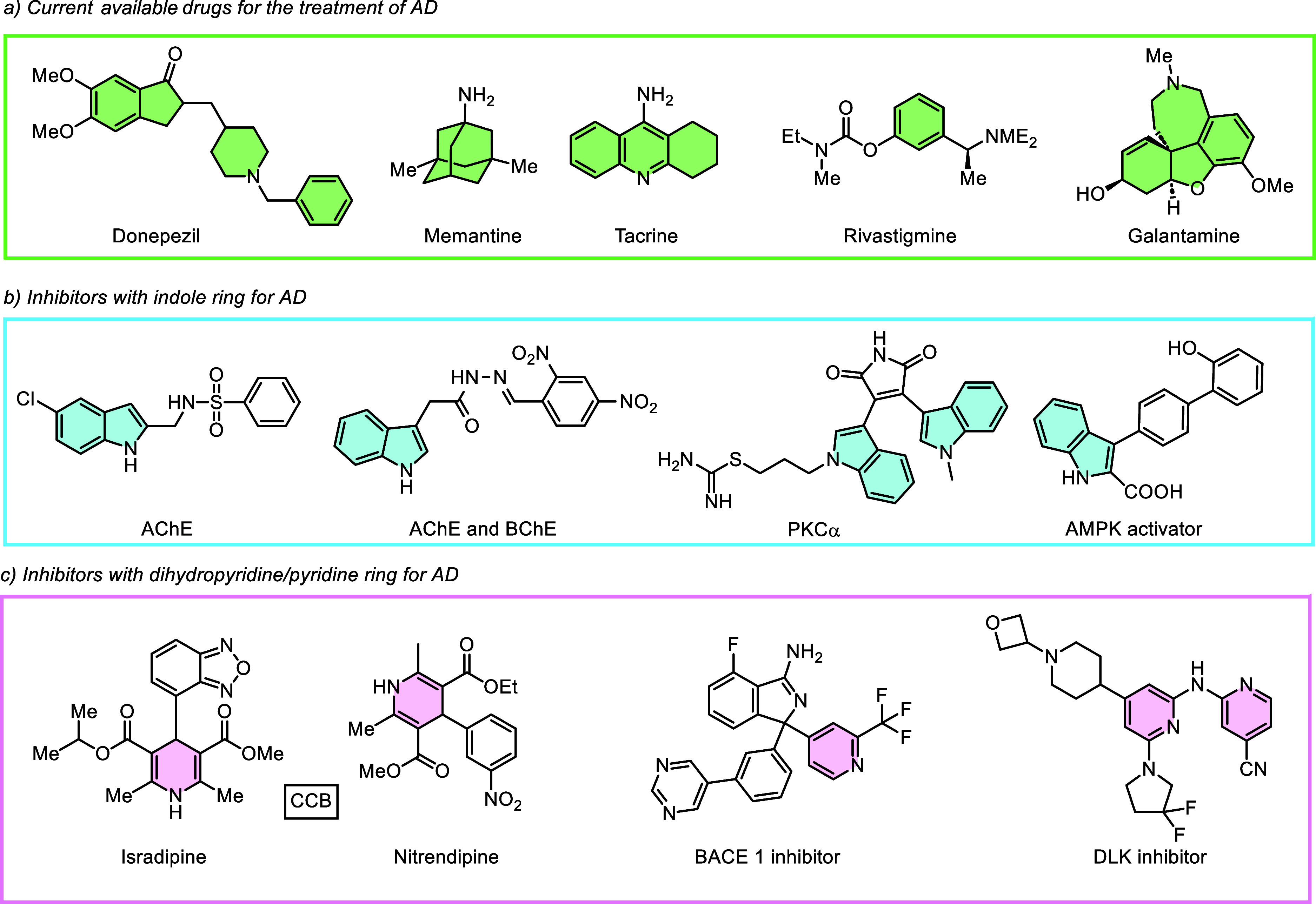
Representative bioactive profiles for Alzheimer’s disease (AD).

**Figure 2:**
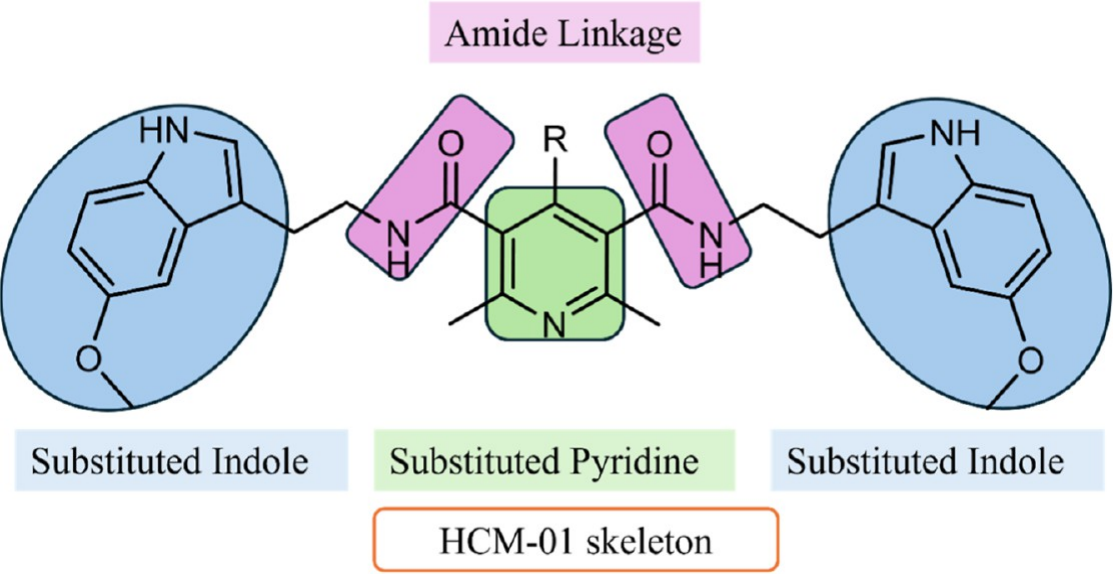
General representation of **HCM-01** skeleton.

Another prominent theory, the tau hypothesis, attributes neuronal death and cognitive decline to the accumulation and spread of hyperphosphorylated tau protein, forming neurofibrillary tangles (NFT). Although tau-directed monoclonal antibodies such as ABBV-8E12 have been explored, clinical trials have yet to demonstrate significant cognitive improvement.^4,5^

In addition, oxidative stress has been identified as a major contributor to AD progression. Reactive oxygen species (ROS) damage neurons, promote amyloid beta (Aβ) aggregation, and facilitate NFT formation. Antioxidant-based interventions have shown promise in preclinical models, representing another potential therapeutic avenue.^6,7^

Central to many of these pathological alterations is the excitatory neurotransmitter glutamate, which plays a pivotal role in neuroplasticity and memory. A recent meta-analysis revealed that elevated levels of glutamate and aspartate in the synaptic cleft can lead to neuronal death through excitotoxicity, primarily due to impaired glutamate reuptake.^8^ Among the therapeutic strategies targeting glutamate toxicity, riluzole has shown efficacy by enhancing glutamate reuptake through excitatory amino acid transporter 2 (EAAT2/GLT-1), blocking α-amino-3-hydroxy-5-methyl-4-isoxazolepropionic acid (AMPA) and *N*-methyl-d-aspartate (NMDA) receptors, and inhibiting voltage-dependent sodium and calcium channels.^9^ It is important to note that glutamate-mediated excitotoxicity does not necessarily result in immediate neuronal cell death but may initially manifest as functional synaptic dysfunction, metabolic impairment, and oxidative imbalance, particularly during early stages of neurodegenerative processes.^10,11^

In this context, astrocytes play a critical neuroprotective role by maintaining extracellular glutamate homeostasis, primarily through EAAT2-mediated clearance, thereby limiting excitotoxic stress before irreversible neuronal loss occurs.^12,13^ Accordingly, experimental astrocyte-based in vitro cell culture models that focus on functional glutamatergic stress and astrocyte-mediated regulation, rather than acute cytotoxicity, may provide valuable insights into early disease mechanisms and therapeutic intervention strategies.^14^

The multifactorial nature of AD has spurred growing interest in developing multifunctional therapeutic agents targeting diverse pathological pathways.^15–18^ Researchers have explored combining established pharmacophores to generate novel compounds with enhanced efficacy.^19–21^

Melatonin, a naturally occurring hormone, exemplifies a promising approach due to its broad neuroprotective properties. Its indole core has demonstrated potent anti-Alzheimer’s effects, including antioxidant activity,^19,22^ modulation of tau hyperphosphorylation, inhibition of cholinesterases, and regulation of mitochondrial energy metabolism.^23,24^ Moreover, the indole ring has proven its potential to exert anti-Alzheimer’s properties by inhibiting protein aggregation and NFT formation through modulation of key targets, such as protein kinase C alpha (PKCα),^25^ protein kinase R-like endoplasmic reticulum kinase (PERK),^26^ AMP-activated protein kinase (AMPK),^27^ and monoamine oxidases B (MAO-B) enzymes.^28^

Furthermore, antihypertensive calcium channel blockers (CCBs), such as isradipine^29^ and nitrendipine^30^ have established anti-Alzheimer’s and neuroprotective properties^31–33^ through their antioxidants,^34^ anti-inflammatory,^35^ antiphospholipase,^36^ antitau,^37^ and antiamyloid^38^ activities. Most CCBs share a dihydropyridine (DHP) nucleus, which confers a wide range of biological properties, especially for AD.^30,39,40^ Additionally, the pyridine aromatic ring has also demonstrated diverse neuroprotective and anti-Alzheimer characteristics by inhibiting AChE, as observed with compounds such as tacrine and huperzine A,^41–43^ along with inhibition of β-site amyloid precursor protein cleaving enzyme-1 (BACE1) enzyme^44^ and dual leucine zipper kinase (DLK) Figure 1c.^45^

These findings highlight the potential of pyridine-based scaffolds as multifunctional agents capable of modulating multiple key pathways involved in AD. Based on this rationale, we synthesized the hybrid molecule *N* ^3^,*N* ^5^-bis­(2-(5-methoxy-1*H*-indol-3-yl)­ethyl)-2,6-dimethyl-4-(2-nitrophenyl)­pyridine-3,5-dicarboxamide (**HCM-01**), as described in previously filed patents,^46–50^ which combines indole and pyridine pharmacophores into a single scaffold to achieve a synergistic effect against oxidative stress and glutamate-induced excitotoxicity.

To assess the neuroprotective efficacy of **HCM-01** under AD-like conditions, the intracerebroventricular (ICV) streptozotocin (STZ)-induced rat model was employed to mimic the metabolic and cognitive impairments associated with sporadic AD. This model induces key pathological hallmarks of sporadic AD including brain insulin resistance, oxidative stress, and cholinergic dysfunction. However, it should be noted that while the ICV-STZ model reproduces several key features of sporadic AD, it does not fully recapitulate the complex and multifactorial nature of the human disease, particularly amyloid and tau pathologies.^51^

In this context, the study was designed to evaluate the neuroprotective potential and underlying mechanisms of **HCM-01** through complementary in vitro, in vivo, and in silico approaches. We hypothesized that integrating indole and pyridine scaffolds within a single molecule could produce a multitarget effect capable of mitigating oxidative stress and excitotoxicity. This strategy aimed to provide preclinical evidence supporting **HCM-01** as a potential therapeutic candidate for the treatment of sporadic AD.

## Results and Discussion

### Chemistry

The compound **HCM-01** was designed by combining the CCB nifedipine and the neurotransmitter melatonin into a single molecular skeleton. DHP ring, the primary core of nifedipine, was oxidized to a pyridine ring and subsequently subjected to ester hydrolysis to obtain its carboxylic acid form (**5**). The pyridine ring has been extensively studied for its wide range of biological properties.^52,53^ Its presence significantly influences the pharmacological profile of the entire molecule by enhancing potency, permeability, metabolic stability, and ligand–protein binding.^41^

In parallel, the indole moiety, derived from melatonin, was incorporated due to its strong radical-scavenging and neuroprotective properties, as well as its occurrence in neurotransmitters such as serotonin and several therapeutic agents.^54^ Previous studies have also demonstrated that indole-containing structures can ameliorate neuroinflammation and inhibit cholinesterase activity, both of which are critical in AD pathogenesis.^55^ Hence, integrating the pyridine nucleus of modified nifedipine,^4,5^ with the 5-methoxytryptamine fragment obtained from melatonin deacetylation^2^ was hypothesized to yield a multifunctional neuroprotective scaffold.

Experimentally, **HCM-01** was synthesized through a four-step route (Scheme 1). Melatonin (**1**) was deacetylated under acidic conditions (10% sulfuric acid), followed by basification, to yield crude 5-methoxytryptamine (**2**) (Scheme 1, step 1). Subsequently, the dihydropyridine ring of nifedipine (3) was oxidized to a pyridine ring with phenyliodine bis­(trifluoroacetate) (PIFA) in dichloromethane (DCM) at room temperature, affording intermediate (**4**) (Scheme 1, step 2), which was further subjected to ester hydrolysis to generate the carboxylic acid derivative (**5**) (Scheme 1, step 3). In the final step, amine (2) and carboxylic acid (**5**) intermediates underwent an amidation reaction in the presence of *N*,*N′*-dicyclohexylcarbodiimide (DCC) and 1-hydroxybenzotriazole (HOBt) to afford **HCM-01** in a good yield. Spectroscopic analyses (^1^H, ^13^C NMR, and HRMS) confirmed the proposed structure.

**Scheme 1:**
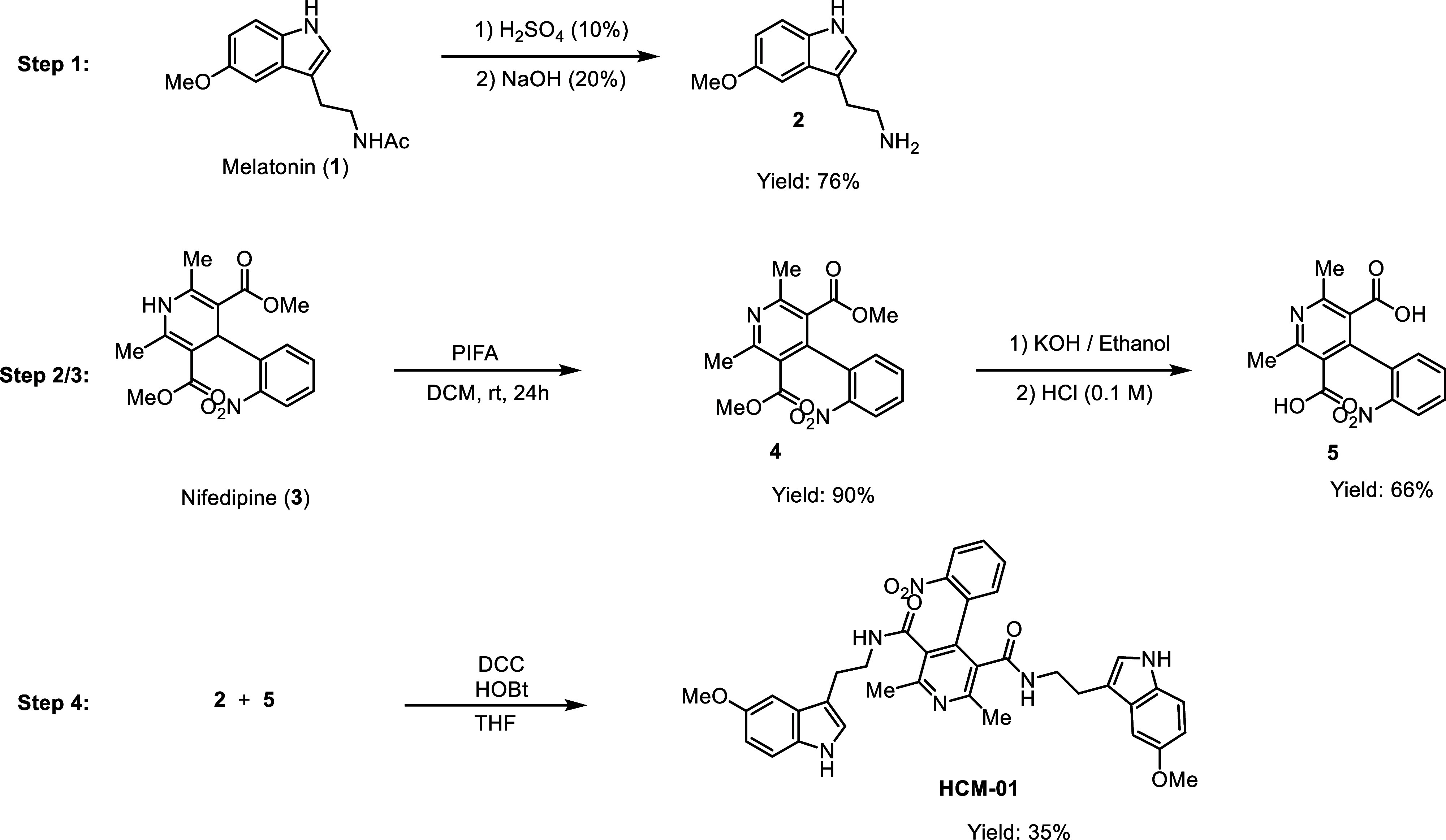
Synthesis Steps of Compound **HCM-01** ^a^

a DCM: dichloromethane, PIFA: phenyliodine bis­(trifluoroacetate); DCC: *N*,*N′*-dicyclohexylcarbodiimide; HOBt: 1-hydroxybenzotriazole, THF: tetrahydrofurane.

### In Vitro Studies

#### Effects of **HCM-01** on Glutamate-Induced Excitotoxicity in Primary Neuronal Cultures

In vitro, neurotoxicity can be experimentally induced through various well-established stressors that mimic pathological changes observed in neurodegenerative diseases Agents such as glutamate,^56^ okadaic acid,^57^ and hydrogen peroxide (H_2_O_2_)^58^ have been shown to induce excitotoxicity, mitochondrial dysfunction, and oxidative stress in neuronal cultures. Among these, glutamate exposure is widely employed to model excitotoxic mechanisms relevant to AD, particularly in vulnerable neuronal populations.

Glutamate, a fundamental excitatory neurotransmitter in the central nervous system (CNS), contributes to neurodegenerative processes by inducing excitotoxic neuronal injury, oxidative stress, and synaptic dysfunction when extracellular homeostasis is disrupted.^59^ Maintenance of glutamate homeostasis is primarily mediated by astrocytic excitatory amino acid transporter 2 (EAAT2/GLT-1),^60^ which clears excess glutamate from the synaptic cleft and thereby limits overactivation of ionotropic glutamate receptors, including NMDA, AMPA, and kainate receptors (KARs).^61,62^

Given that AD predominantly impacts the hippocampal and cortical areas, which play major roles in cognitive function and neurogenesis within the brain,^63^ agents that selectively provide neuroprotection to these regions are considered valuable in Alzheimer’s research. Moreover, growing evidence highlights the involvement of the cerebellum in cognitive function, and its impairment has been associated with cognitive deficits in AD.^64^

Primary neuronal cultures were exposed to glutamate (10 μM) to induce excitotoxic stress, and cell viability was assessed using the MTT assay to evaluate the protective potential of **HCM-01**. **HCM-01** was tested at concentrations of 0.1, 1, 10, 100, and 1000 μM, while memantine and donepezil were used as reference compounds at 10 μM.

In this study, cell cultures derived from the hippocampus, cortex, and cerebellum were exposed to glutamate at a concentration of 10^–5^ M to induce excitotoxicity to assess the neuroprotective potential of **HCM-01** against glutamate-induced toxicity using the [3-(4,5-dimethylthiazol-2-yl)-2,5-diphenyltetrazolium bromide] (MTT) assay. The MTT assay results revealed that glutamate exposure reduced cell viability to 69%, 70%, and 81% in hippocampal, cortical, and cerebellar cultures, respectively (Figure 3a–c). Memantine moderately improved cell viability in hippocampal and cortical cultures to 87% and 88%, respectively, while donepezil showed a mild increase in viability to 89% and 91% in hippocampal and cortical cultures, respectively (Figure 3a,b). The test compound **HCM-01** significantly improved cell viability at concentrations of 0.1 μM and 1 μM. Specifically, in hippocampal cells, **HCM-01** demonstrated significant neuroprotective effects, increasing cell viability to 107% and 125% at 0.1 μM and 1 μM concentrations, respectively, representing a 14% and 50% increase compared to the glutamate-treated control (Figure 3a). In cortical cultures, **HCM-01** exhibited substantial neuroprotection, with cell viability increasing to 115% at 10 μM concentration (Figure 3b).

**Figure 3:**
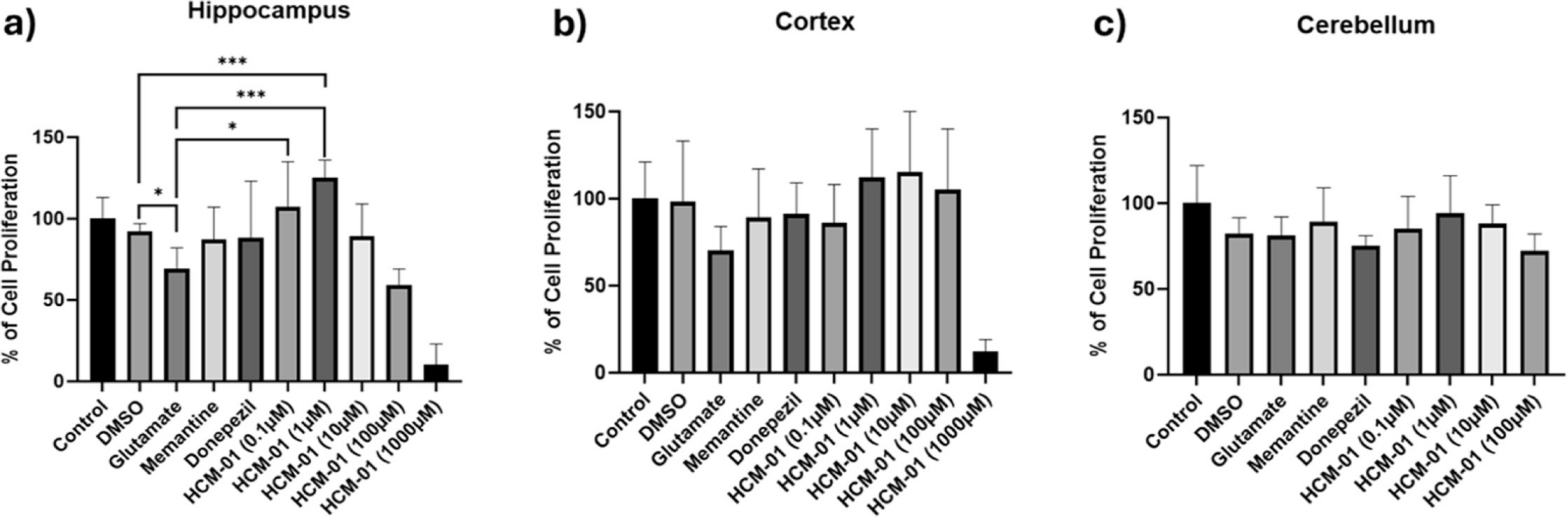
Effects of **HCM-01** on glutamate-induced excitotoxic stress in primary culture. MTT-based cell viability analysis in primary neuronal cultures derived from (a) hippocampus, (b) cortex, and (c) cerebellum following exposure to glutamate (10 μM) and treatment with **HCM-01** or reference compounds. Data are presented as mean ± SD of six independent experiments (*n* = 6). Statistical analysis was performed using Student’s *t*-test for pairwise comparisons with the glutamate group. *p < 0.05, **p < 0.01, ***p < 0.001 compared with the glutamate group.

These results indicate that **HCM-01** possesses superior neuroprotective properties compared with both memantine and donepezil in cortical cultures. In cerebellar cultures, **HCM-01** demonstrated moderate neuroprotective effects at concentrations of 0.1 μM, 1 μM, which were still superior to the effects observed with memantine and donepezil (Figure 3c).

At higher concentrations (100 and 1000 μM), **HCM-01** reduced cell viability across all neuronal cultures, indicating a concentration-dependent cytotoxic effect.

Collectively, these results demonstrate that **HCM-01** confers significant neuroprotection against glutamate-induced excitotoxicity in primary hippocampal neurons and region-dependent functional protection in cortical and cerebellar cultures within a defined concentration window.

#### Modulation of Astrocytic Viability and EAAT2 Expression in In Vitro Cell Culture Models

The intrinsic glutamate-buffering system functions through astrocyte–neuron interactions as part of the glutamate–glutamine cycle, regulating glutamate uptake and preventing excitotoxicity via EAAT2-mediated conversion of glutamate into glutamine (Figure 4a). Cell viability analysis showed no significant differences between the groups under the applied conditions. Unlike primary neuronal cultures, glutamate exposure did not cause notable cytotoxicity in astrocytes, consistent with their intrinsic glutamate-buffering and neuroprotective role (Figure 4b). Western blot analysis showed that EAAT2 expression increased only in the glutamate + **HCM-01** cotreatment group. Glutamate alone, vehicle, control, and Memantine did not produce a significant change in EAAT2 levels (Figure 4c,d). These findings indicate that **HCM-01** enhances astrocytic glutamate-handling capacity under excitotoxic conditions rather than altering basal EAAT2 expression. While the in vitro results mainly indicate increased EAAT2 expression, this effect may partly reflect a glutamate-mediated adaptive compensatory mechanism rather than direct transporter affinity.^65^ Therefore, these findings may provide an initial indication of potential biological relevance, warranting further validation through affinity-based and uptake assays to confirm direct interactions and clarify the underlying biological efficacy.

**Figure 4:**
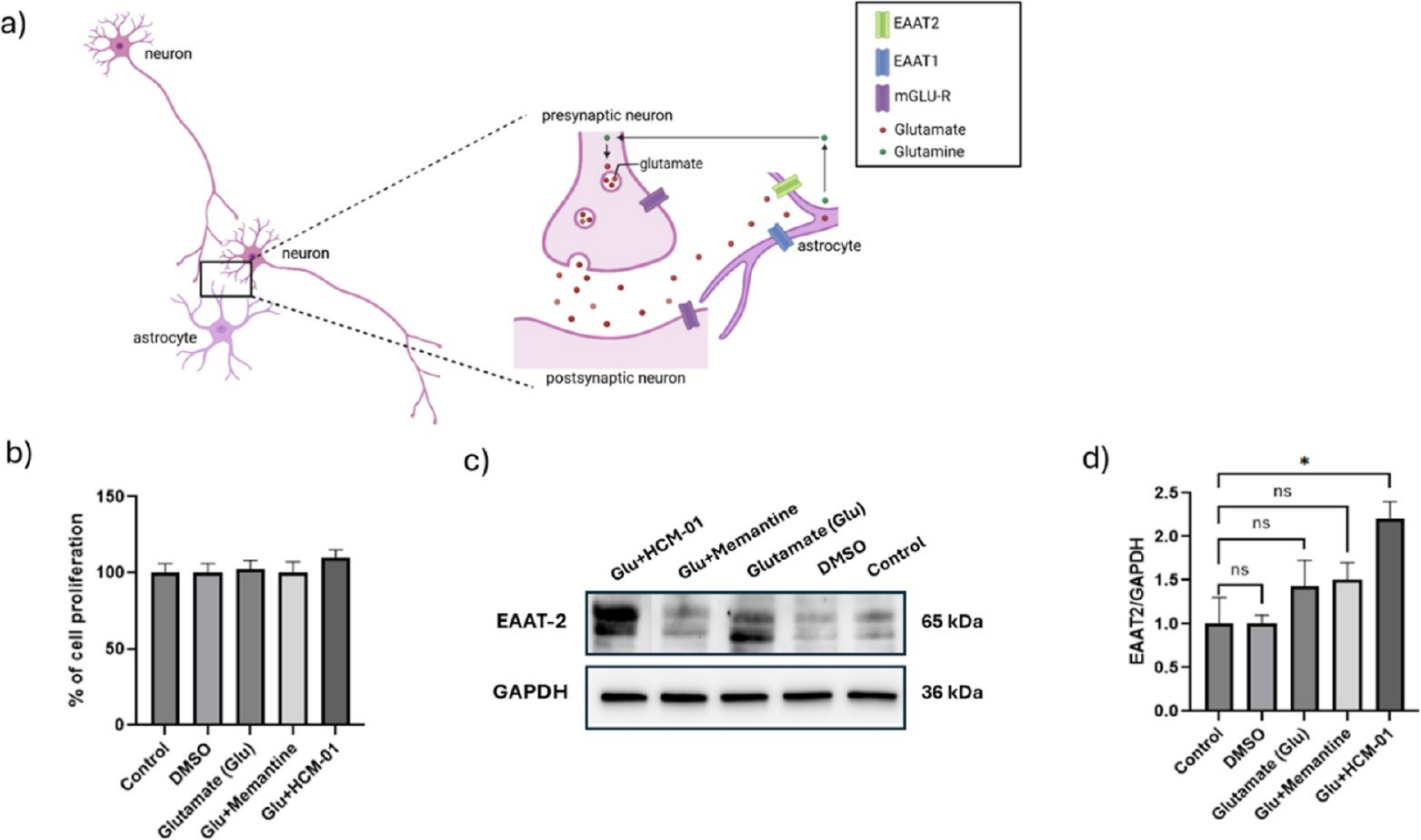
Modulation of astrocytic EAAT2 expression by **HCM-01** under glutamate exposure. (Created with BioRender.com). (a) Schematic representation for the glutamine–glutamate cycle between neurons and astrocytes. (b) Astrocyte cell viability following glutamate exposure and cotreatment. (c) Representative immunoblot images of EAAT2 expression (d) Densitometric analysis of EAAT2 normalized to GAPDH and expressed fold changes relative to control. Data are presented as mean ± SD of three independent experiments (*n* = 3). Statistical analysis was performed using Student’s *t*-test for pairwise comparisons with the control group. *p < 0.05, ns: nonsignificant compared with the control group.

#### Modulation of Oxidative/Antioxidative Balance by **HCM-01**

High extracellular glutamate levels are one of the primary causes of oxidative stress and increased ROS within the brain, which eventually leads to neurodegeneration. Diverse reports have demonstrated the ability of antioxidants to restore redox homeostasis and support neuronal function.^66,67^

To assess whether the neuroprotective effects of **HCM-01** are associated with modulation of oxidative stress, oxidative/antioxidative changes were evaluated in primary hippocampal neuronal cultures by measuring total antioxidant capacity (TAC) and total oxidant status (TOS) levels under glutamate-induced stress conditions (Table 1). The results indicated that at a concentration of 10 μM, **HCM-01** treatment increased antioxidant levels compared to the control group. Although TAC values declined at 1 μM and 10^–1^ μM concentrations of **HCM-01**, they remained higher than those observed in the glutamate-treated control. It was also observed that the oxidant levels in **HCM-01**–treated samples were lower at 1 μM compared to the glutamate control group. However, at 10^–1^ μM, oxidant levels increased slightly relative to the glutamate control.

**Table 1:** Effect of **HCM-01** on TAC and TOS Levels^a^

group	TAC ± SD (mmol Trolox E/L)	TOS ± SD (mmol H_2_O_2_ E/L)
control	8,67 ± 0.75	4.65 ± 0.28
glutamate control	4.5 ± 0.45	9.59 ± 0.87
**HCM-01** (10^4^ μM)	9.63 ± 0.84**	3.96 ± 0.23**
**HCM-01** (103 μM)	9.44 ± 0.77**	3.32 ± 0.28**
**HCM-01** (102 μM)	9.65 ± 0.83**	3.41 ± 0.31**
**HCM-01** (10 μM)	9.48 ± 0.67**	3.85 ± 0.27**
**HCM-01** (1 μM)	7.32 ± 5.32*	5.77 ± 4.89**
**HCM-01** (10–1 μM)	5.56 ± 0.61	8.66 ± 0.68

a Data are presented as mean ± SD. Statistical analysis was performed using Student’s *t*-test for pairwise comparisons with the glutamate control group. *p < 0.05, **p < 0.001 compared with the glutamate control group.

Consistent with these findings, TOS levels were significantly reduced in **HCM-01**-treated groups across most concentrations compared with the glutamate control. The most pronounced reduction in oxidant status was observed at low micromolar concentrations of **HCM-01**. At the lowest tested concentration (10^–1^ μM), TOS levels showed a partial increase relative to glutamate control, indicating a loss of antioxidant efficacy at subthreshold concentrations.

Overall, treatment with **HCM-01** enhanced TAC levels and generally reduced TOS levels compared to the glutamate-induced stress conditions, indicating improved oxidative/antioxidative balance and suggesting potential indirect antioxidant properties to its neuroprotective effects. These findings are consistent with the observed improvements in neuronal viability and astrocytic glutamate-handling capacity, suggesting that redox modulation represents a complementary mechanism underlying the functional effects of **HCM-01**. Further investigations are warranted to evaluate specific oxidative damage markers, such as lipid peroxidation and protein carbonylation, and underlying mechanisms involved.

### In Vivo Studies

In vivo models of neurotoxicity are commonly induced using agents such as aluminum chloride (AlCl_3_),^68^ kainic acid,^69^ and STZ.^70^ In this study, we aimed to evaluate the neuroprotective effects of **HCM-01** in STZ-induced Alzheimer’s disease-like rat model, where STZ (3 mg/kg) was bilaterally injected into the lateral ventricles under stereotaxic guidance to induce brain insulin resistance, oxidative stress, and cognitive impairment characteristic of sporadic AD. The compound **HCM-01** was administered either orally (P.O.) at doses of 50 or 100 mg/kg, or intraperitoneally (I.P.) at a dose of 50 mg/kg, once daily for 24 days. For comparison, a memantine-treated group (10 mg/kg, P.O.) was included as a reference control. The neuroprotective effects were assessed through behavioral paradigms, including the Morris Water Maze (MWM), passive avoidance (PA) and locomotor activity assays.

#### Morris Water Maze (MWM) Results

The MWM is a behavioral test that evaluate spatial learning in rodents. It relies on distal visual cues to guide navigation from various start locations around the perimeter of an open swimming arena toward a submerged escape platform. Spatial learning is assessed across repeated training trials, whereas reference memory is determined by the animal’s preference for the former platform location during probe trials in which the platform is removed.

According to the MWM results (Figure 5), the data from the untreated sham-operated control and the untreated healthy control groups were comparable. The negative control and sham-operated groups collectively demonstrated the fastest learning in the MWM test. Conversely, the STZ-treated (patient control) group displayed impaired spatial learning, and in some cases, failed to acquire the task. The **HCM-01**-treated group and the memantine control group showed comparable results, exhibiting a moderate level of improvement compared to the patient control. However, their improvement in learning ability did not reach the level observed in the negative control group, which demonstrated superior spatial learning performance overall.

**Figure 5:**
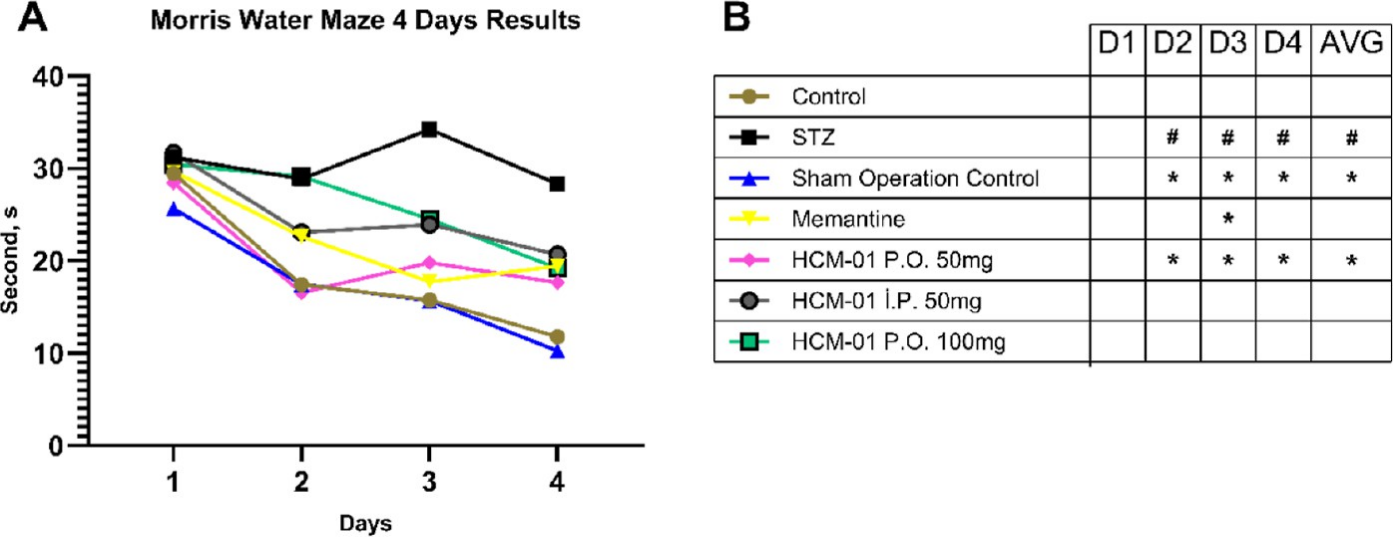
(A) The time of learning in seconds over 4 days after administration of 50 mg (P.O.and I.P.), 100 mg of **HCM-01** (P.O.), and memantine (10 mg/kg). Each group (*n* = 8); (B) Statistical comparison of daily and 4 day averages of Morris water maze results # Indicates statistically significant difference compared to the control group. * Indicates difference compared to the STZ group. (D: Day, AVG: Average, STZ: Streptozotocin, P.O.: Per Os, I.P.: Intraperitoneal).

#### Passive Avoidance Results

The PA task is a fear-augmented test used to evaluate learning and memory in rodent models of CNS disorders. In this task, nocturnal animals, which are naturally more active at night and sleep during the day, are introduced into a lighted chamber that they instinctively avoid by entering a dark chamber. However, the dark chamber is connected to a mild electric current, serving as an aversive stimulus. With repeated exposure, healthy animals are expected to develop a memory of the stimulus and adapt by remaining in the lighted, electric-free chamber. In contrast, sporadic AD-like model is unable to retain this memory and tend to re-enter the dark chamber.

In this experiment, the time that animals spent in the electric-free lighted chamber was recorded in seconds on days 19–20 and 40–41. For each group (Figure 6). Animals in the AD model group (treated with STZ) displayed impaired learning and memory, as evidenced by their shorter time spent in the lighted chamber and frequent re-entries into the dark chamber. In contrast, animals treated with **HCM-01** demonstrated significant improvement, with the duration in the lighted chamber reaching up to 300 s on day 41. This result indicates that **HCM-01** facilitated memory development in the STZ-induced Alzheimer-like pathology model. Similarly, animals treated with memantine showed comparable improvements. Notably, both the untreated negative control group and the **HCM-01**-treated Alzheimer-induced group displayed similar outcomes, suggesting that **HCM-01** was effective in enhancing memory retention under these conditions.

**Figure 6:**
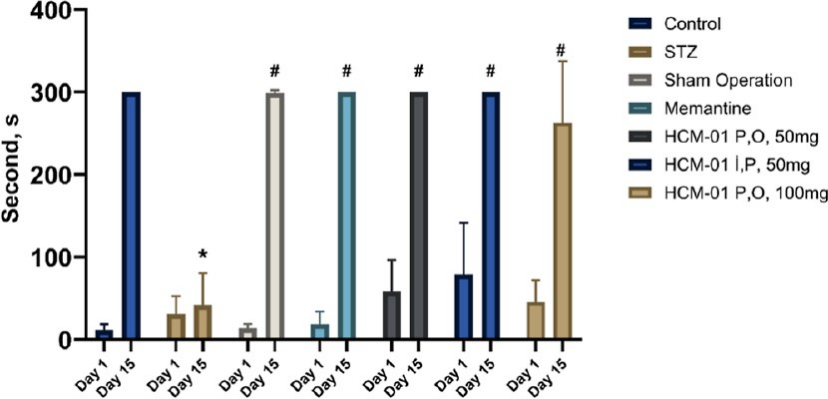
Time spent by rats in the electric-free compartment (s) on days 19 and 41 of the experiment. * Indicates a significant difference compared to the control group. # Indicates a significant difference compared to the STZ group. (STZ: Streptozotocin, P.O.: Per Os, I.P.: Intraperitoneal).

#### Locomotor Activity Results

To explicitly rule out the possibility of motor confounds in the interpretation of cognitive outcomes, spontaneous locomotion was quantified in infrared beam–equipped open-field activity cages. Three end points were prespecified: total distance (cm), mean speed (cm/s; distance divided by session duration), and resting percentage (immobility index). Across all experimental groups, no main effect of group was detected for total distance, mean speed, or resting percentage (all *p* > 0.05), and all Tukey contrasts were nonsignificant. Visually, individual observations were found to be clustered with substantial overlap between groups and similar dispersion around the mean (Figure 7a–c; individual values with mean ± SD), indicating the absence of systematic hypo- or hyperactivity as well as comparable immobility across conditions. Taken together, these findings demonstrate intact and equivalent gross motor performance among the groups, thereby supporting the conclusion that the memory effects reported below are unlikely to be attributable to mobility deficits.

**Figure 7:**
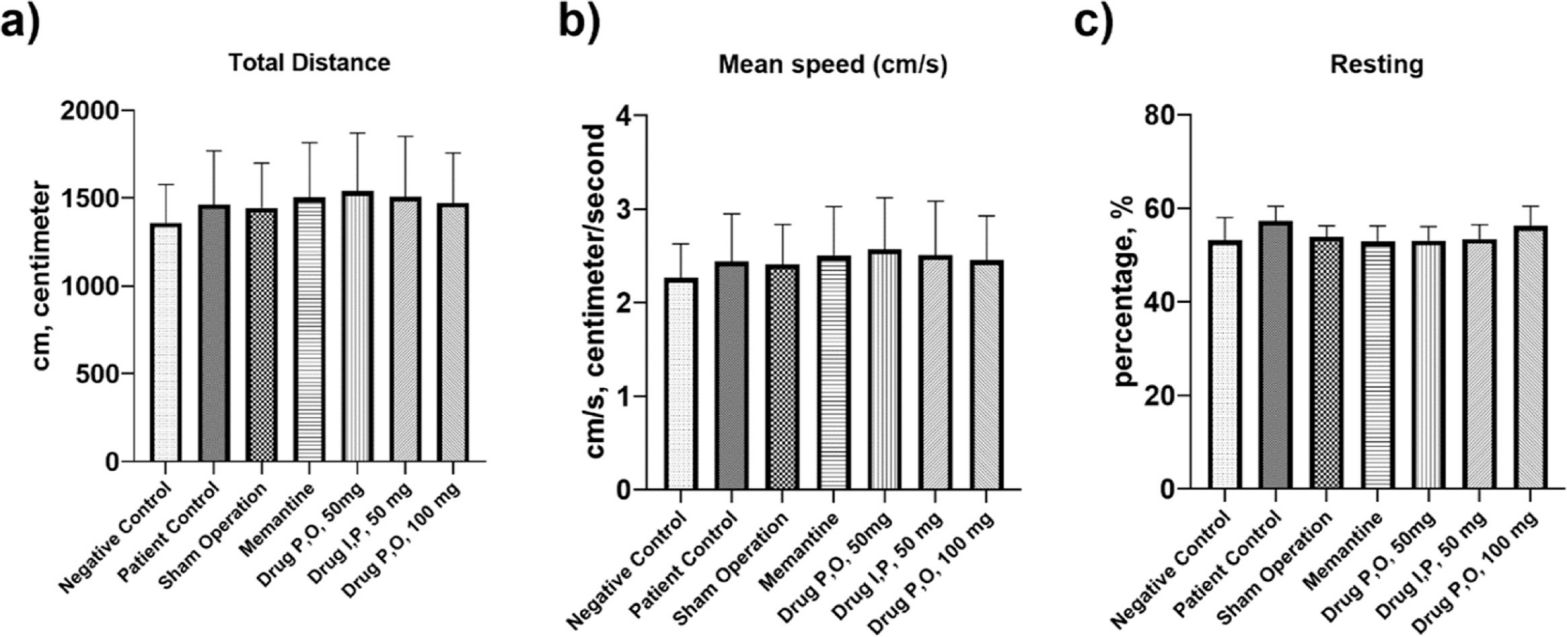
Locomotor activity results. (a) Total distance (cm) traveled, (b) mean speed (cm/s), and (c) resting time percentage (%) (*p* > 0.05).

These findings are consistent with the growing body of evidence supporting EAAT2 induction as an effective neuroprotective strategy. Previous studies have shown that EAAT2 activators, such as β-lactam antibiotics (e.g., ceftriaxone) and translational modulators like LDN/OSU-0212320, enhance EAAT2 expression in astrocytes and restore glutamate uptake in rodent models and cultured cells.^71^ For instance, LDN/OSU-0212320 has been shown to activate EAAT2 translation, leading to an increase in functional glutamate uptake within hours and providing neuroprotection against excitotoxic injury in astrocyte–neuron cocultures.^72^ Similarly, ceftriaxone treatment elevated EAAT2 expression up to 4-fold in rodent brains, delayed motor neuron degeneration, and improved survival and behavioral performance in Amyotrophic Lateral Sclerosis (ALS) models.^71,73^

It has also been reported that alterations in the glutamatergic system may develop in the STZ-induced diabetes model, particularly that reduced glutamate uptake in the hippocampus may facilitate synaptic glutamate accumulation, thereby promoting NMDA/AMPA-mediated excitotoxicty.^74^ Therefore, some of the neurological and histological changes observed in our study may be explained through processes associated with glutamate-mediated excitotoxic mechanisms.

#### Histopathological Examination

In addition to behavioral assessments, histopathological analyses of the hippocampal cornu Ammonis (CA1/CA2 and CA3) regions, along with immunohistochemical evaluations of AChE, Tau, and β-amyloid, were performed to investigate the mechanisms underlying **HCM-01**-mediated neuroprotection.

Histopathological examination revealed statistically significant differences among the experimental groups (Table 2; Figures 8–10). In negative control and sham-operated groups, the neuronal architecture of the CA1/CA2 and CA3 regions appeared normal and well-preserved. In contrast, the treatment groups displayed varying degrees of neuronal pyknosis and degeneration within the CA1/CA2 and CA3 subfields.

**Table 2:** Statistical Analysis of Degenerative Changes Observed in Neurons

groups	CA1/CA2	CA3
negative control	0.00 ± 0.00^a^	0.33 ± 0.51^a^
sham operation	0.16 ± 0.40^a^	0.16 ± 0.40^a^
STZ	0.83 ± 0.40^b^	2.66 ± 0.51^b^
memantine (10 mg/kg)	0.33 ± 0.51^a^	1.66 ± 0.51^c^
**HCM-01** (P.O. 50 mg/kg)	0.16 ± 0.40^a^	1.83 ± 0.40^c^
**HCM-01** (P.O. 100 mg/kg)	0.16 ± 0.40^a^	1.66 ± 0.51^c^
**HCM-01** (I.P. 50 mg/kg)	0.16 ± 0.40^a^	1.66 ± 0.51^c^

**Figure 8:**
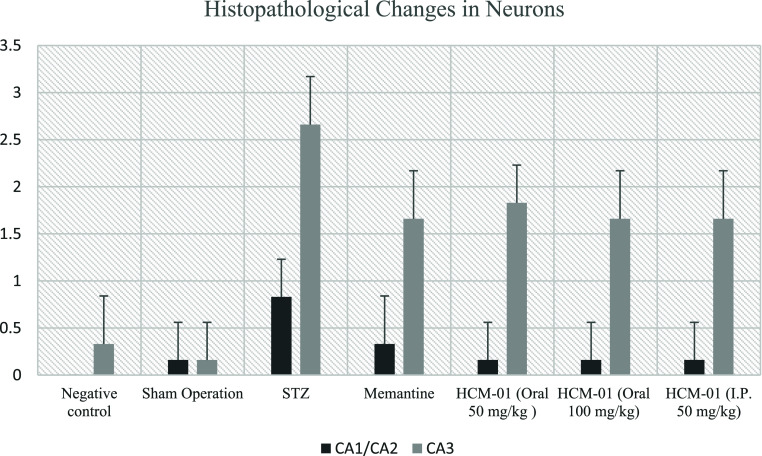
Degenerative changes observed in hippocampal CA1/CA2 and CA3 regions.

**9:**
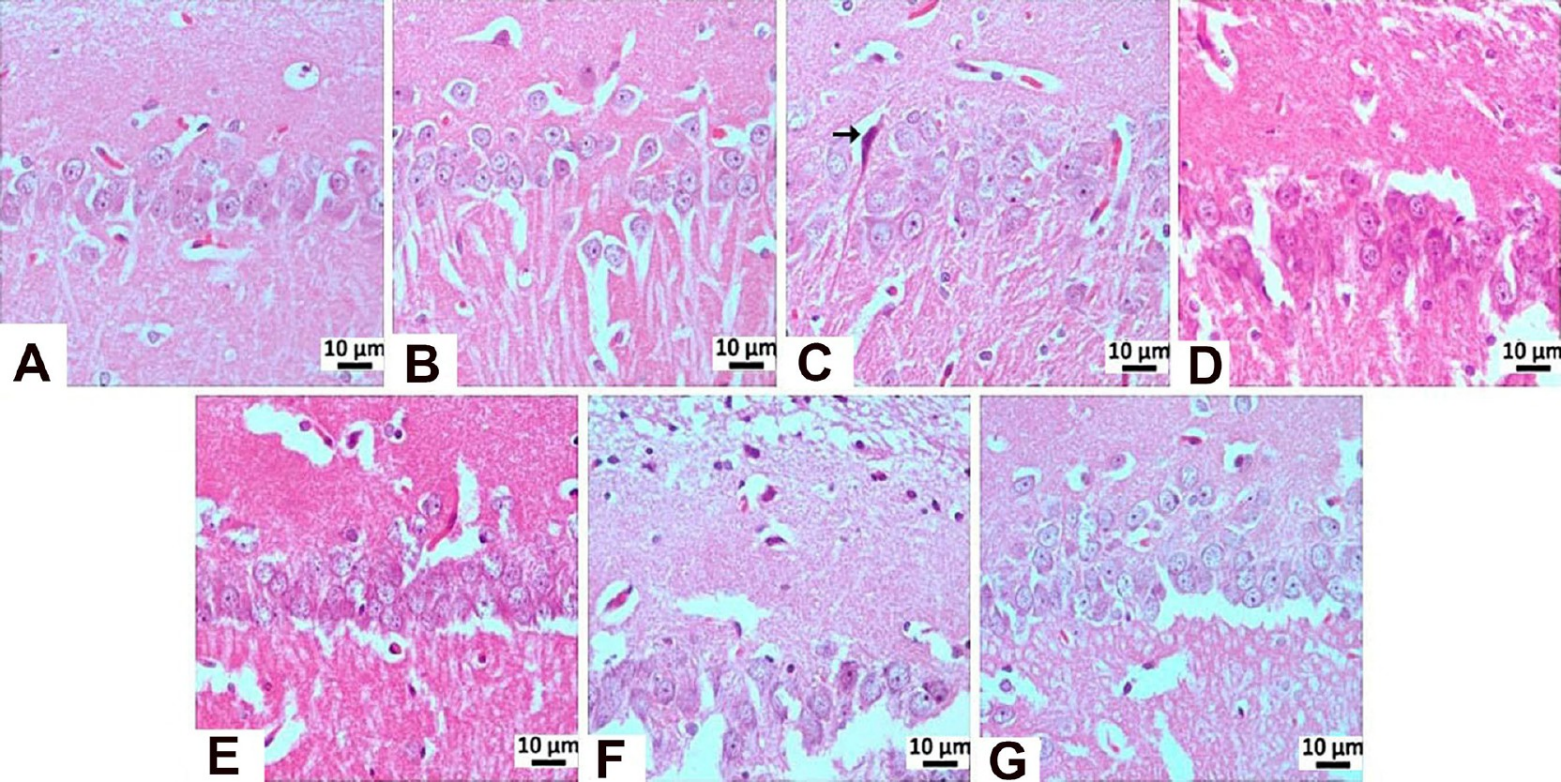
Histopathological view of the CA1/2 region: (A) negative control group, (B) sham operated groups showing normal histological appearance, (C) STZ group, mildly pyknotic neurons (arrow), (D) memantine group (10 mg/kg), (E) **HCM-01** (P.O. 50 mg/kg) group, (F) **HCM-01** (P.O. 100 mg/kg) group, (G) **HCM-01** (I.P. 50 mg/kg) groups showing normal histological appearance, hippocampus, H–E stain. (40×).

**Figure 10:**
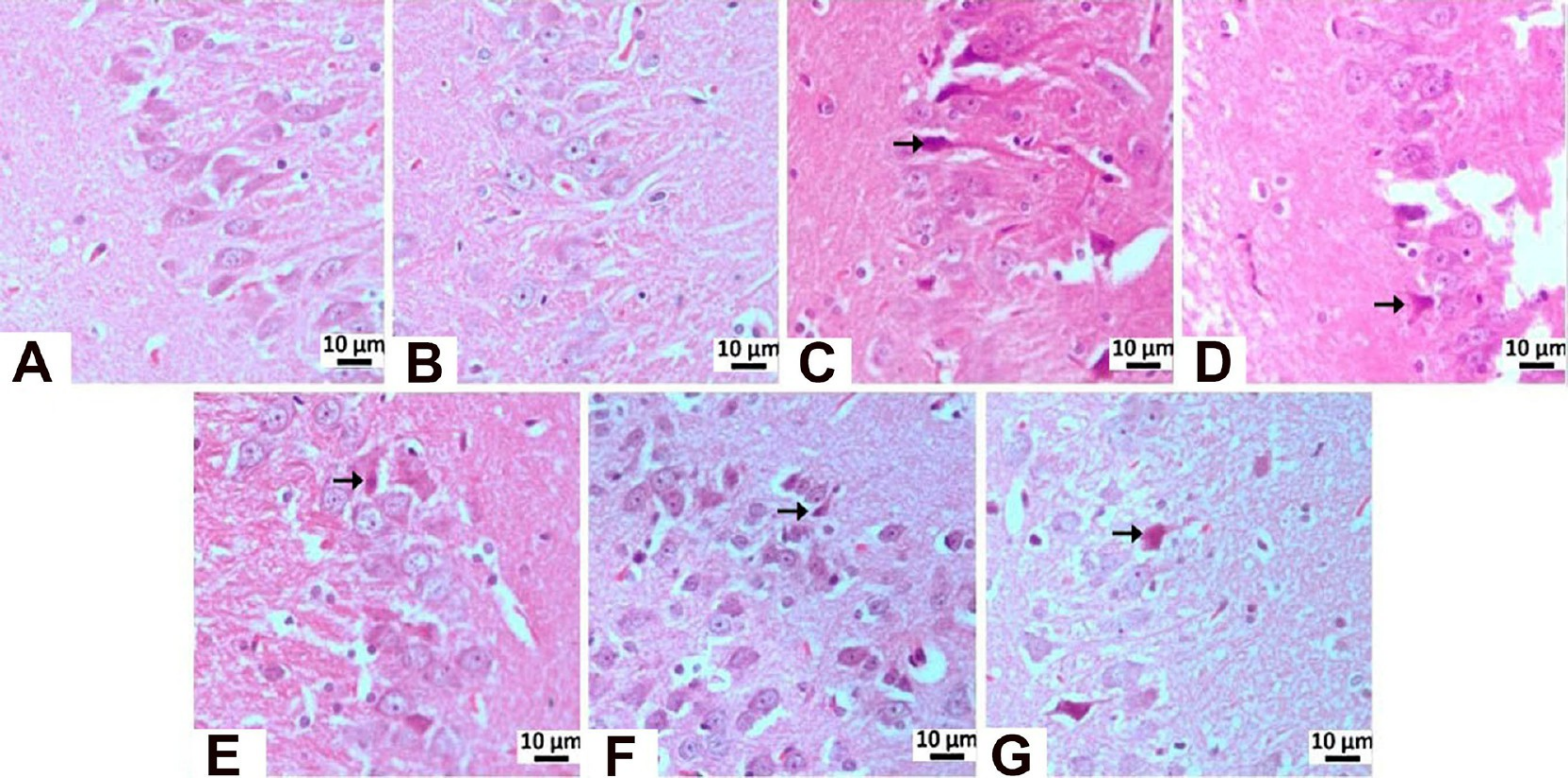
Histopathological view of the CA3 region: (A) negative control group, (B) Sham Operated groups showing normal histological appearance, (C) STZ group-Severely, (D) memantine (10 mg/kg) group-moderately, (E) **HCM-01** (P.O. 50 mg/kg) group-moderately, (F) **HCM-01** (P.O. 100 mg/kg) group-moderately, (G) **HCM-01** (I.P. 50 mg/kg) Neurons with moderate pyknotic changes (arrow), hippocampus, H–E stain. (40×).

In the STZ control group, mild neuronal degeneration was observed in the CA1/CA2 region, whereas no histopathological abnormalities were detected in the memantine (10 mg/kg), **HCM-01** (P.O. 50 mg/kg), **HCM-01** (oral 100 mg/kg), and **HCM-01** (I.P. 50 mg/kg) groups. In the CA3 region of the positive control group, severe neurodegeneration was observed. In contrast, moderate neuronal degeneration was observed in the memantine (10 mg/kg), **HCM-01** (P.O. 50 mg/kg), **HCM-01** (P.O. 100 mg/kg), and **HCM-01** (I.P. 50 mg/kg). Microscopically, pyknosis, representing a stage of necrosis, was characterized by neurons showing degenerative changes, including dark, shrunken, and pyknotic nuclei.

#### Immunohistochemical Examinations

In immunohistochemical staining for Tau, moderate immunopositivity was observed in the CA1/2 region in the STZ, memantine (10 mg/kg), and **HCM-01** (P.O. 100 mg/kg) groups, whereas mild immunopositivity was detected in **HCM-01** (P.O. 50 mg/kg) and **HCM-01** (I.P. 50 mg/kg). In the CA3 region, very severe immunopositivity was observed in the STZ group, indicating pronounced Tau pathology consistent with neurodegeneration. Severe immunopositivity in the **HCM-01** (P.O. 100 mg/kg) group, moderate immunopositivity in both the memantine (10 mg/kg) and **HCM-01** (P.O. 50 mg/kg) groups, and mild immunopositivity in the **HCM-01** (I.P. 50 mg/kg) groups, suggesting a dose- and route-dependent attenuation of Tau pathology (Table 3; Figures 11–13).

**Table 3:** Immunohistochemical Staining of Tau^a^

groups	CA1/CA2	CA3
negative control	0.83 ± 0.40^a^	1.16 ± 0.40^a^
sham operation	0.83 ± 0.40^a^	1.16 ± 0.40^a^
**STZ**	1.83 ± 0.40^b^	3.66 ± 0.51^b^
memantine (10 mg/kg)	1.66 ± 0.51^b^	1.83 ± 0.40^c^
**HCM-01** (P.O. 50 mg/kg)	1.16 ± 0.40^a^	2.00 ± 0.00^c^
**HCM-01** (P.O. 100 mg/kg)	1.66 ± 0.51^b^	2.83 ± 0.40^d^
**HCM-01** (I.P. 50 mg/kg)	1.16 ± 0.40^a^	1.33 ± 0.51^a^

a ^a,b,c,d^ It indicates the difference between groups in the same column (*p* < 0.05).

**Figure 11:**
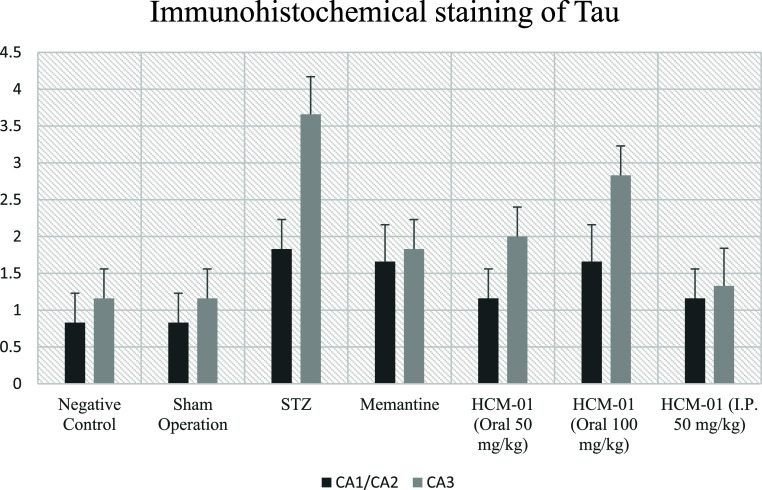
Immunohistochemical staining of Tau.

**12:**
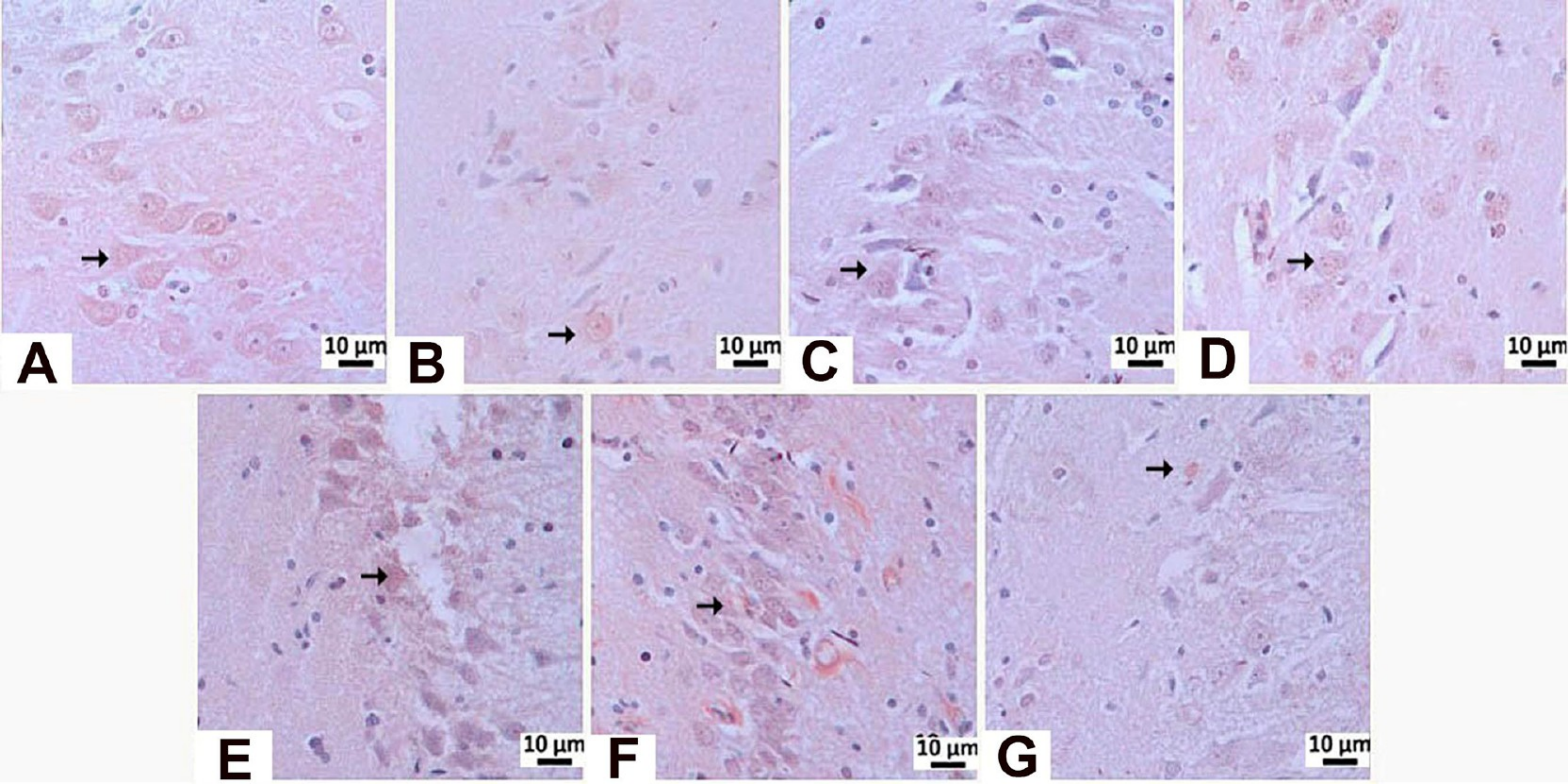
Tau immunopositivity in the CA3 region: (A) negative control group, mild level, (B) sham operated group-mild level, (C) STZ group-very severe level, (D) memantine (10 mg/kg) group-moderate level (E) **HCM-01** (P.O. 50 mg/kg) moderate level, (F) **HCM-01** (P.O. 100 mg/kg) group-severe level, (G) **HCM01** (I.P. 50 mg/kg) group-mild level of immunopositivity (arrow). Hippocampus, IHC. (40×).

**Figure 13:**
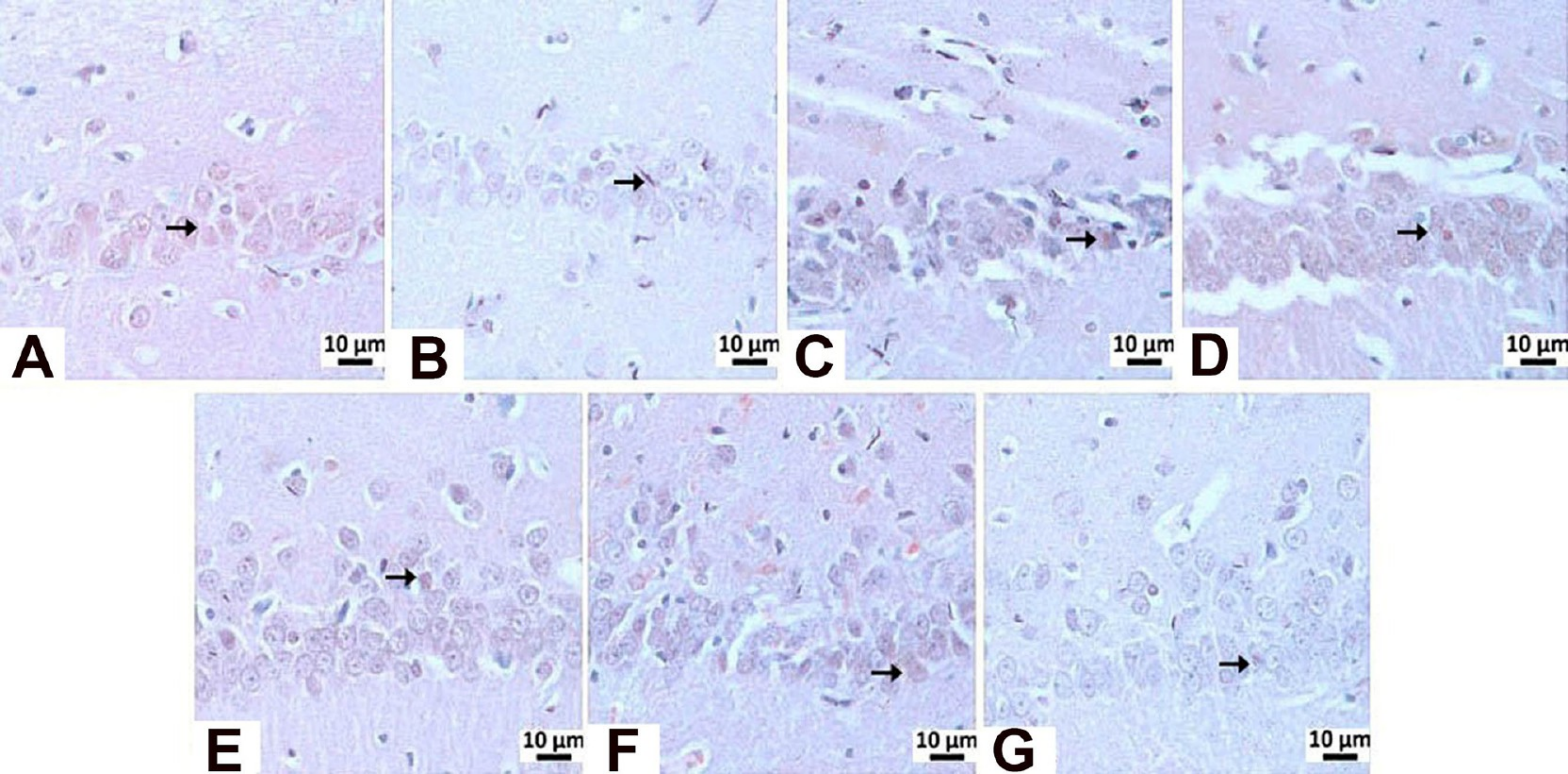
Tau immunopositivity in the CA1/2 region: (A) Negative control group-mild level, (B) Sham Operated group-mild level, (C) STZ group-moderate level, (D) memantine (10 mg/kg) group-moderate level, (E) **HCM-01** (P.O. 50 mg/kg) group-mild level, (F) **HCM-01** (P.O. 100 mg/kg)-moderate level, (G) **HCM-01** (I.P. 50 mg/kg)-mild level of immunopositivity (arrow). Hippocampus, IHC. (40×).

In immunohistochemical staining for β-Amyloid, mild immunopositivity was detected only in the STZ group within the CA1/CA2 region, whereas no immunopositivity was observed in any other group. In the CA3 region, severe immunopositivity was observed in the STZ group, mild immunopositivity in the memantine-treated group, and complete absence of immunopositivity in all **HCM-01**–treated groups (Table 4; Figures 14–16).

**Table 4:** Immunohistochemical Staining with β-Amyloid^a^

groups	CA1/CA2	CA3
negative control	0.33 ± 0.51^a^	0.16 ± 0.40^a^
sham operation	0.16 ± 0.40^a^	0.33 ± 0.51^a^
STZ	0.16 ± 0.40^b^	2.83 ± 0.40^b^
memantine	0.33 ± 0.51^a^	1.66 ± 0.51^c^
**HCM-01** (P.O. 50 mg/kg)	0.33 ± 0.51^a^	0.16 ± 0.40^a^
**HCM-01** (P.O. 100 mg/kg)	0.33 ± 0.51^a^	0.33 ± 0.51^a^
**HCM-01** (I.P. 50 mg/kg)	0.16 ± 0.40^a^	0.16 ± 0.40^a^

a ^a,b,c,d^ It indicates the difference between groups in the same column (*p* < 0.05).

**Figure 14:**
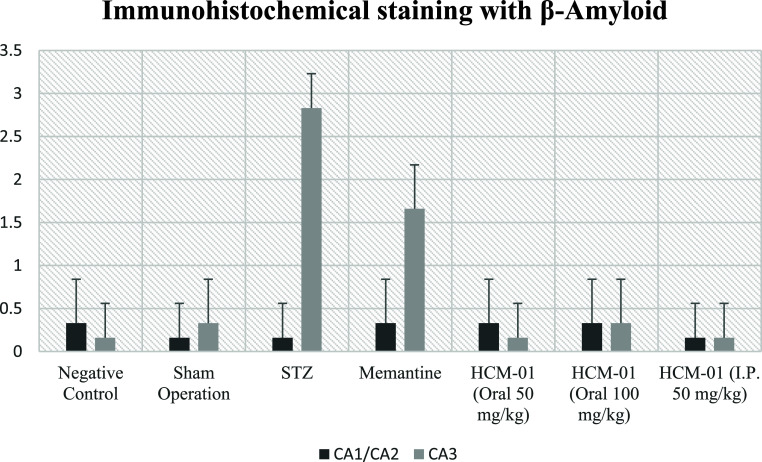
Immunohistochemical staining with β-Amyloid.

**15:**
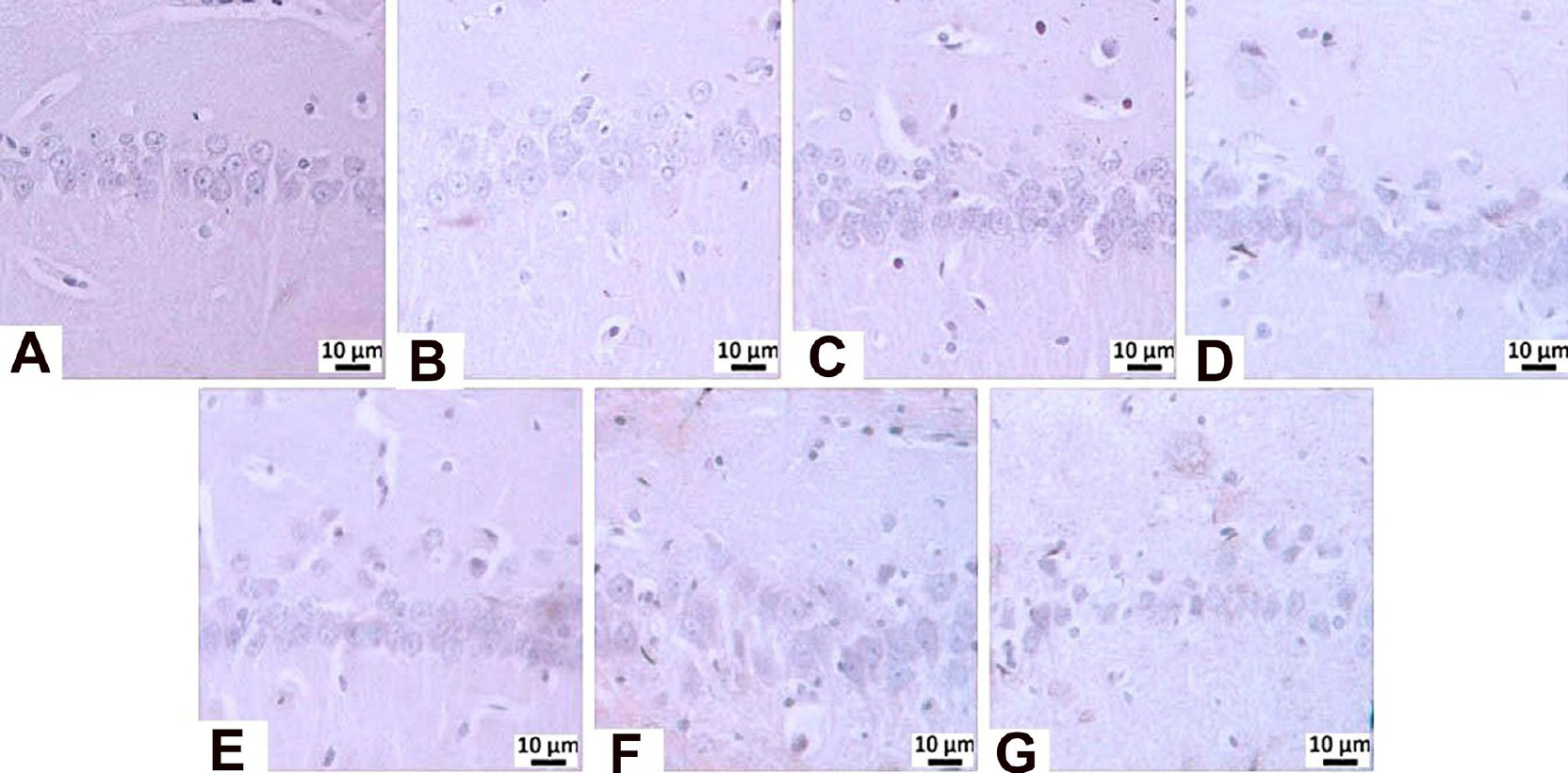
β-Amyloid immunopositivity in the CA1/2 region: (A) negative control group, (b) sham operated group-immunonegativity, (C) STZ group-mild level of immunopositivity (arrow), (D) memantine (10 mg/kg) group, (E) **HCM-01** (P.O. 50 mg/kg) group, (F) **HCM-01** (P.O. 100 mg/kg) group, (G) **HCM-01** (I.P. 50 mg/kg) group-immunonegativity. Hippocampus, IHC, (40×).

**Figure 16:**
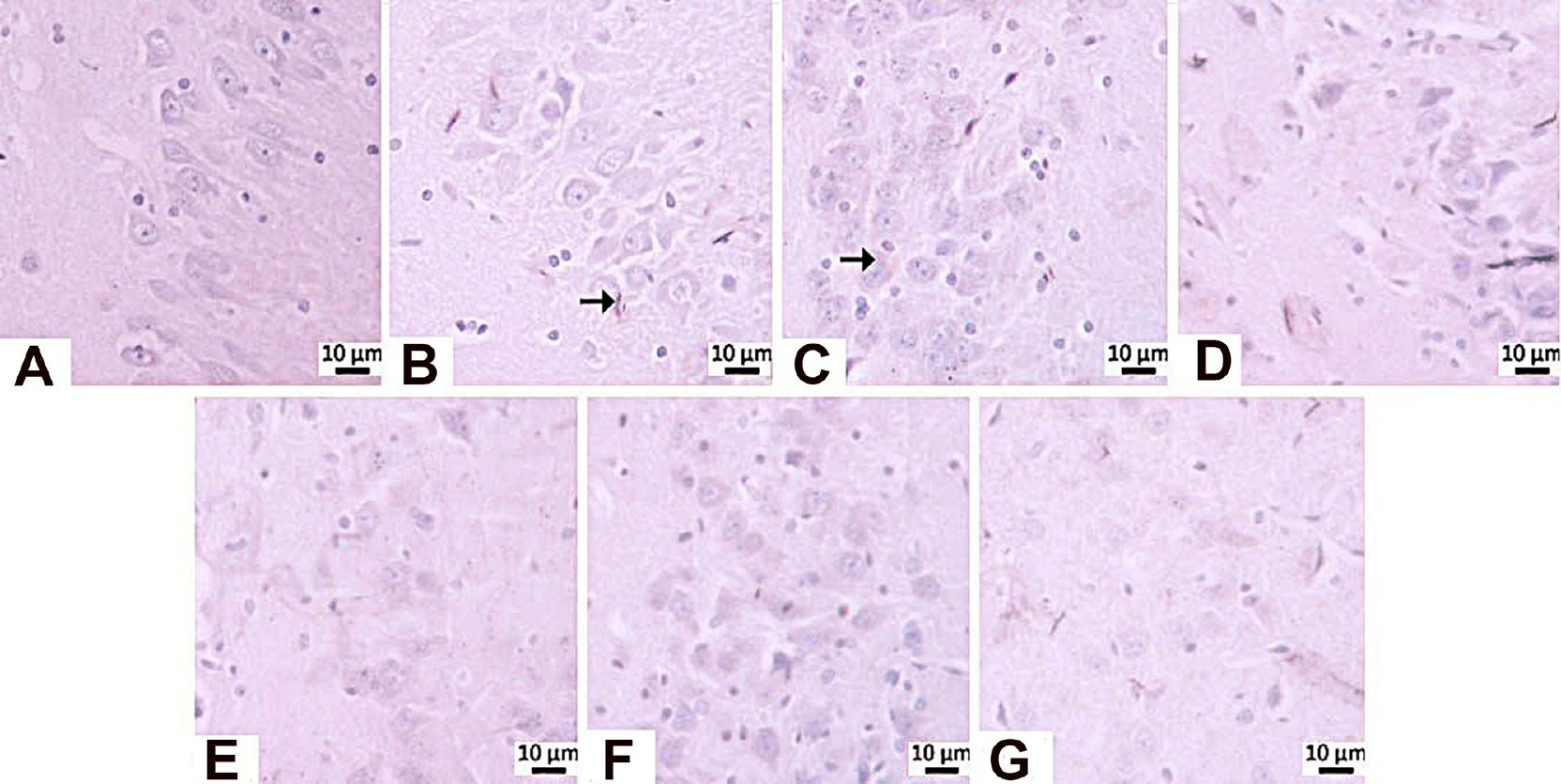
β-Amyloid immunopositivity in the CA3 region: (A) Negative control group, (B) Sham Operated group-immunonegativity, (C) STZ group-severe level, (D) memantine group-mild level of immunopositivity (arrow), (E) **HCM-01** (P.O. 50 mg/kg) group, (F) **HCM-01** (P.O. 100 mg/kg) group, (G) **HCM-01** (I.P. 50 mg/kg) group-immunonegativity, hippocampus, IHC. (40×).

Given that higher β-amyloid immunopositivity interprets greater plaque burden and neuronal toxicity, the absence of β-amyloid immunopositivity indicates effective inhibition of Aβ aggregation or enhanced clearance. In contrast, memantine treatment resulted in only a partial reduction of β-amyloid deposition, as evidenced by mild residual immunopositivity.

In immunohistochemical staining for AChE, no significant immunopositivity was observed in the CA1/2 region across all groups. In contrast, the CA3 region exhibited differential AChE immunoreactivity: very severe in the STZ group, severe in the memantine-treated group, moderate in the **HCM-01** (P.O. 100 mg/kg) group, and mild in all remaining **HCM-01**–treated groups (Table 5; Figures 17–19).

**Table 5:** Immunohistochemical Staining with AChE^a^

groups	CA1/CA2	CA3
negative control	0.16 ± 0.40^a^	1.00 ± 0.00^a^
sham operation	0.16 ± 0.40^a^	1.00 ± 0.00^a^
STZ	0.16 ± 0.40^a^	3.66 ± 0.51^b^
memantine	0.16 ± 0.40^a^	2.83 ± 0.40^c^
**HCM-01** (P.O. 50 mg/kg)	0.33 ± 0.51^a^	2.16 ± 0.40^d^
**HCM-01** (P.O. 100 mg/kg)	0.16 ± 0.40^a^	1.33 ± 0.40^a^
**HCM-01** (I.P. 50 mg/kg)	0.16 ± 0.40^a^	1.16 ± 0.40^a^

a ^a,b,c,d^ It indicates the difference between groups in the same column (*p* < 0.05).

**Figure 17:**
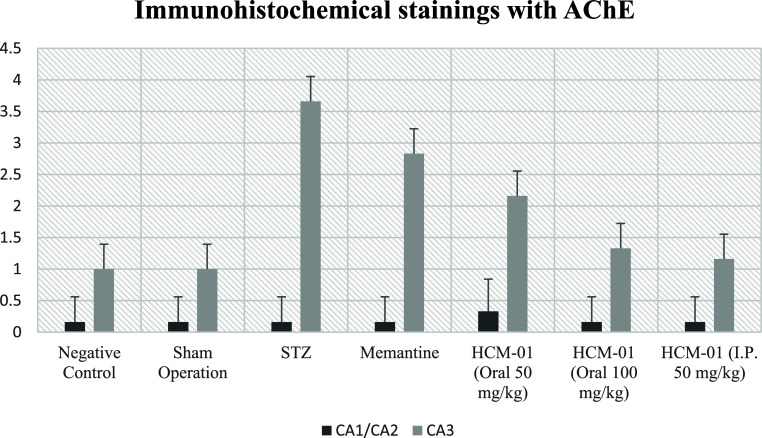
Immunohistochemical staining with AChE.

**18:**
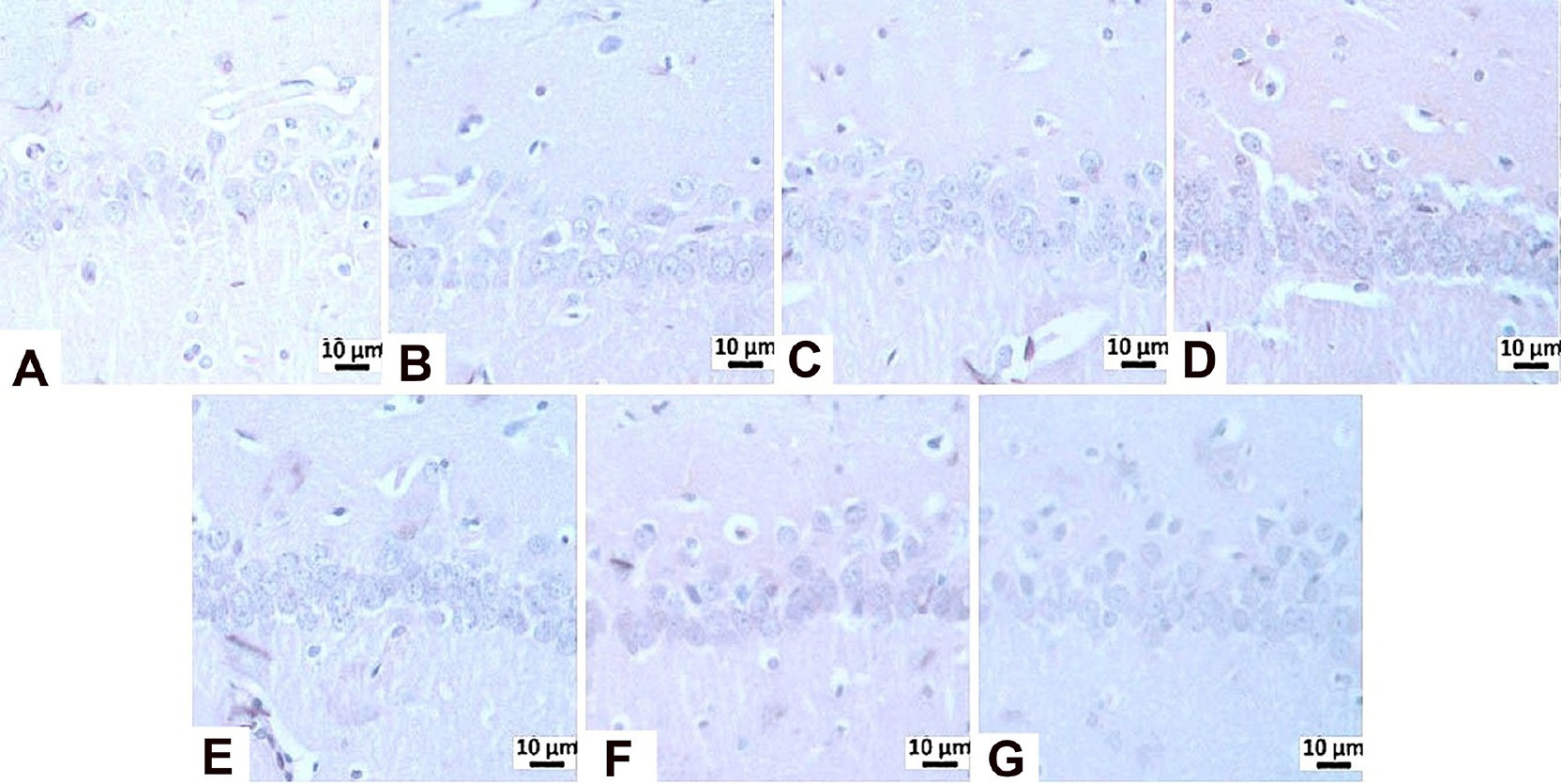
AChE immunonegativity in the CA1/2 region: (A) negative control group, (B) sham operated group, (C) STZ group, (D) memantine (10 mg/kg) group, (E) **HCM-01** (P.O. 50 mg/kg) group, (F) **HCM-01** (P.O. 100 mg/kg) group, (G) **HCM-01** (I.P. 50 mg/kg) group, hippocampus, IHC. (40×).

**Figure 19:**
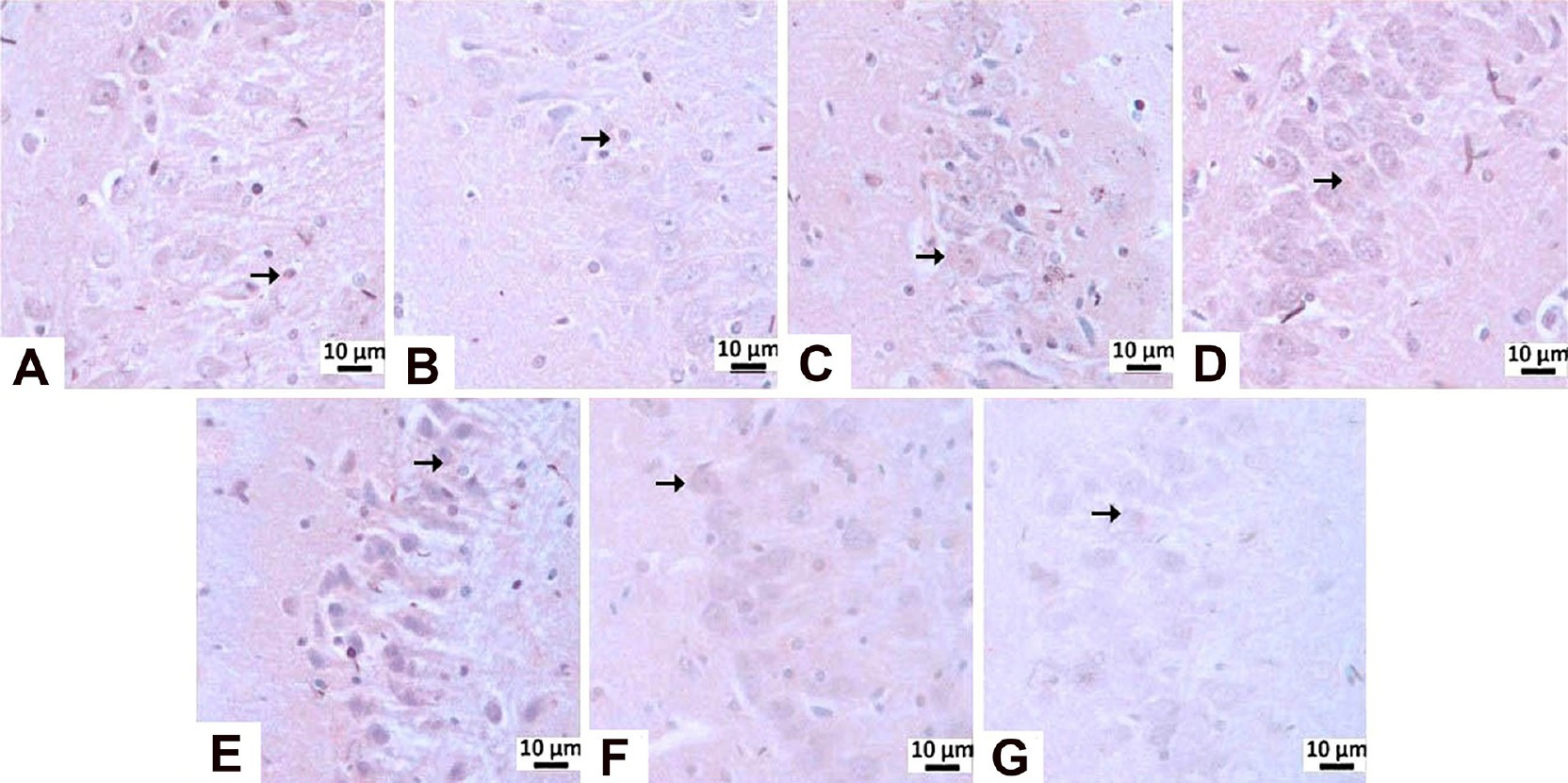
AChE immunopositivity in the CA3 region: (A) negative control group-mild level, (B) sham operated group-Mild level, (C) STZ group, very severe level, (D) memantine (10 mg/kg) group-severe level, (E) **HCM-01** (P.O. 50 mg/kg) group-moderate level, (F) **HCM-01** (P.O. 100 mg/kg) group-Mild level, (G) **HCM-01** (I.P. 50 mg/kg) group-Mild level of immunopositivity (arrow). Hippocampus, IHC. (40×).

AChE is a critical enzyme responsible for acetylcholine breakdown, and its overexpression is associated with cholinergic dysfunction, cognitive deficits, and memory impairment in AD. The marked AChE immunopositivity in the STZ group confirms cholinergic degradation consistent with Alzheimer-like pathology. Notably, **HCM-01** significantly decreased AChE immunopositivity, suggesting a restoration of cholinergic function and supporting its neuroprotective potential.

#### Measurement of HCM01 in Brain Tissues after Behavioral Assessment

Brain tissue samples were collected from six rats that had received an oral dose of 50 mg/kg of **HCM-01**, 60–75 min postadministration, based on preliminary pharmacokinetic studies. The presence of **HCM-01** in brain tissue was confirmed by HRMS analysis. The characteristic ion peaks of **HCM-01** were detected at [M + H]^+^ = 661.28334, [M + 2H]^+^ = 662.28120, [M + 3H]^+^ = 663.28266, and [M + 4H]^+^ = 664.28638 (Table 6 and Figure 20), demonstrating its ability to penetrate the blood–brain barrier.

**Table 6:** HRMS Analysis Results of **HCM-01**

*m*/*z*	z	abund	formula	ion
647.26238	1	8041.2		
**661.28334**		**238935.27**		(M + H)^+^
661.43074		15112.56		
661.49624	1	26906.7		
661.57080		18127.3		
**662.28120**	**1**	**221143.47**	**C**_**37**_ **H**_**37**_ **N**_**6**_ **O**_**6**_	(**M** + 2H)^+^
662.49773	1	12277.18		
**663.28266**	**1**	**57177.05**	**C**_**37**_ **H**_**37**_ **N**_**6**_ **O**_**6**_	**(M** + 3H)^+^
**664.28638**	**1**	**8249.14**	**C**_**37**_ **H**_**37**_ **N**_**6**_ **O**_**6**_	(**M** + 4H)^+^
671.26101	1	81032.48		
672.26480	1	54687.11		
673.26764	1	15920.92		
675.29118	1	8144.67		
683.25846	1	26897.2		
684.26054	1	10653.33		
699.23032	1	7780.94		
703.28779	1	7784.91		

**Figure 20:**
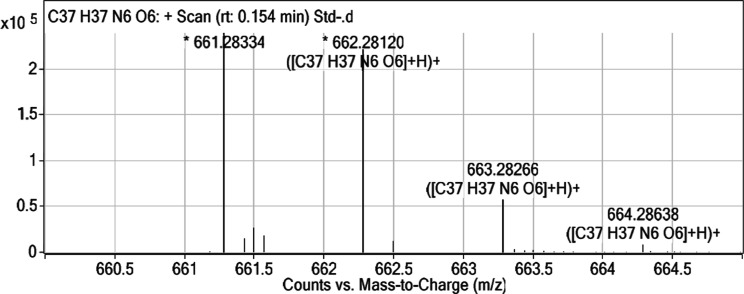
HRMS analysis results of **HCM-01**.

### In Silico Studies

#### Molecular Docking (MD)

The molecular docking (MD) was performed at the allosteric site of EAAT2 to explore the possible interactions of **HCM-01** in the designated molecular environment. The allosteric site was identified based on previously reported structural analyses of EAAT2.^75,76^ The docking results indiacted that **HCM-01** interacts directly with key residues within the allosteric pocket, suggesting a favorable and specific binding profile. The calculated binding free energy further supported the hypothesis that **HCM-01** acts as a potential allosteric activator of EAAT2, potentially contributing to reduced extracellular glutamate levels in the synaptic cleft.^77^

Both rigid and flexible MD analyses were performed using Schrödinger software to comprehensively evaluate the interaction profile and docking scores of **HCM-01**. The binding poses were analyzed and visualized using the Maestro interface and compared with the known positive modulator GT949 (Figure 21).

**Figure 21:**
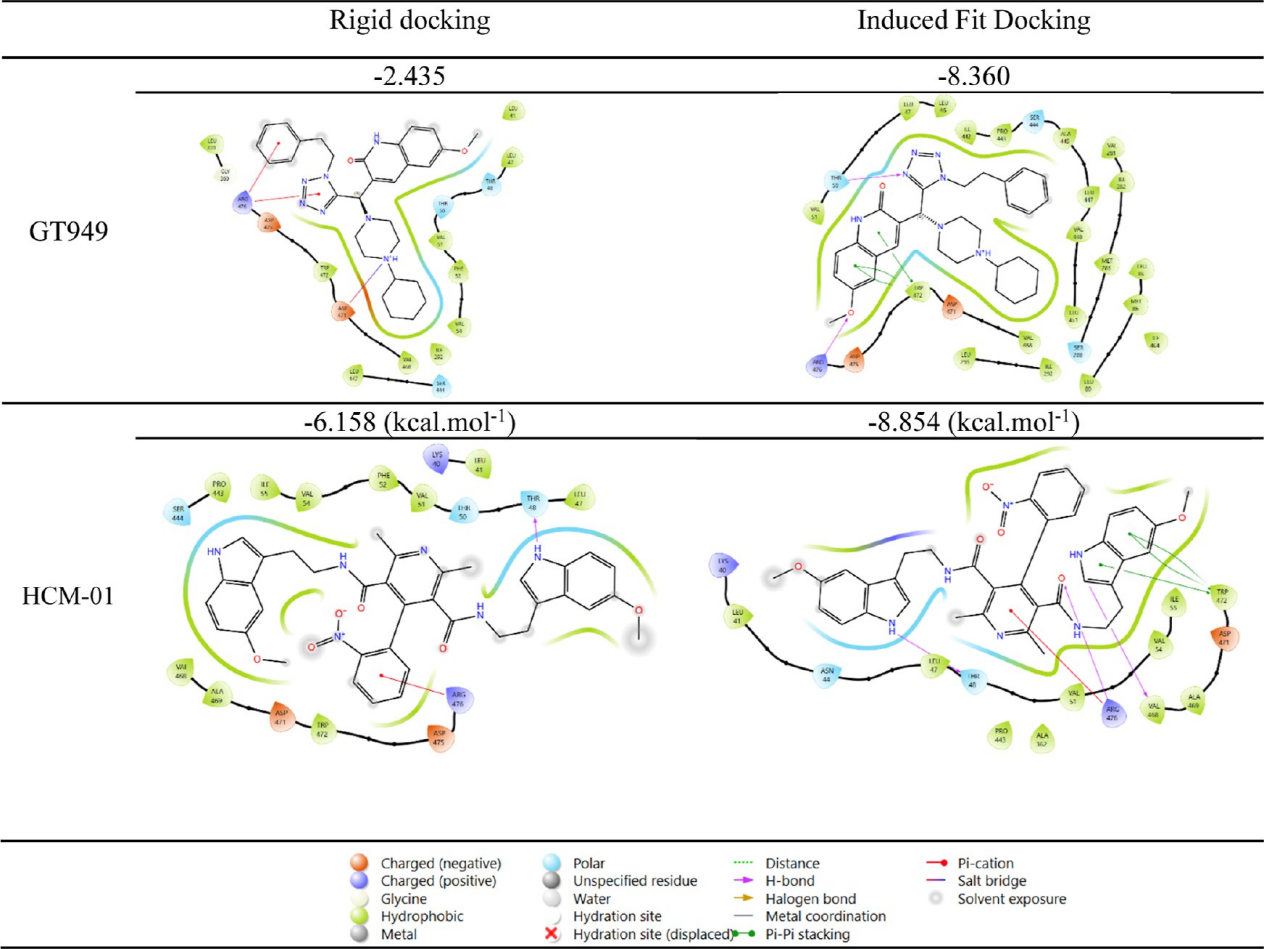
2D interactions and docking scores of **HCM-01** and **GT949** with the EAAT2 transporter.

The Induced-Fit Docking (IFD) analysis of **HCM-01** produced a favorable docking score of −8.854 kcal/mol, comparable to GT949 IFD docking score (−8.360 kcal/mol), suggesting a strong potential binding affinity for the allosteric site of the EAAT2 transporter. In contrast, the rigid docking analysis exhibited a better docking score for **HCM-01** (−6.158 kcal/mol) compared to GT949 (−2.435 kcal/mol), further supporting its superior interaction potential.

According to the 2D ligand interaction diagrams, the interactions were facilitated by hydrogen bonding, hydrophobic contacts, and π–π stacking interactions. In the rigid docking model, a π-cation interaction was observed with ARG476, while the IFD analysis revealed three π–π stacking interactions with TRP472, along with π-cation and hydrogen bonding interactions involving ARG476. Conversely, rigid docking of GT949 showed only two π-cation and salt bridge interactions with ARG476 and ASP471, respectively. The IFD results for GT949, however, revealed three π–π stacking interactions with TRP472 and one hydrogen bond with ARG476.

These findings confirm the stable and specific accommodation of **HCM-01** within the EAAT2 allosteric site, emphasizing its strong binding affinity and adaptability, particularly when transporter conformational flexibility was considered.

#### Molecular Dynamics Simulation

A 200 ns (ns) molecular dynamics simulation (MDS) was performed following the docking studies to evaluate the dynamic stability and conformational behavior of the EAAT2-**HCM-01** complex. The simulation incorporated a water environment and a cell membrane model, encompassing all three EAAT2 transporter chains. These thorough computational studies offer a multidimensional understanding of the molecular interactions and conformational dynamics of **HCM-01** within the designated binding environment.

The stability of a protein–ligand complex can be evaluated by calculating the Root Mean Square Deviation (RMSD), which quantifies the average deviation of atomic positions from the initial conformation over the course of the simulation (Figure 22).

**Figure 22:**
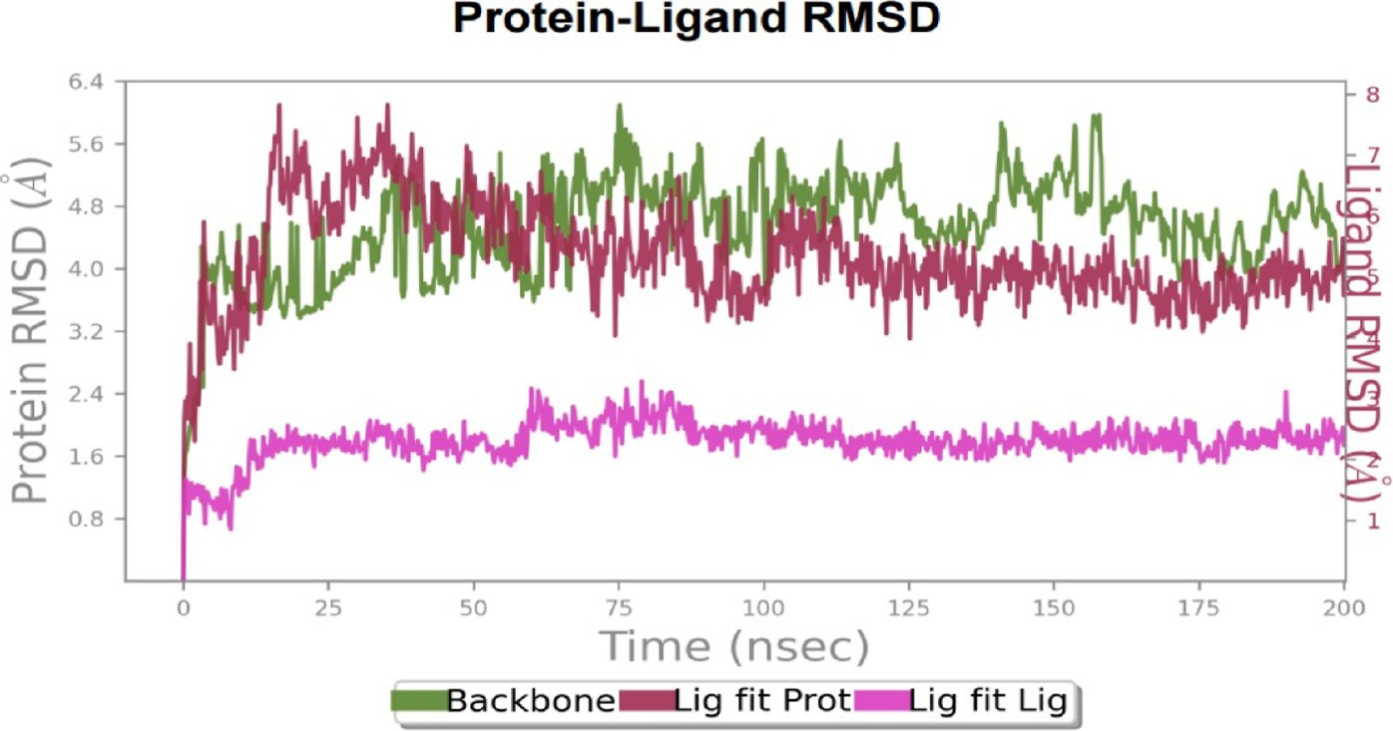
RMSD of the complex **HCM-01**-EAAT2, the ligand, and the backbone over the 200 ns at 300 K.

Initially, the RMSD values of the EAAT2-**HCM-01** complex fluctuated between 3 and 6 Å but eventually stabilized around 4 Å as favorable interactions were established. This stabilization indicates that the ligand’s binding position remained relatively stable, despite minor conformational fluctuations within the protein’s binding pocket. The relatively high overall RMSD values observed for the entire protein-ligand-membrane system primarily reflect the mobility of extramembrane domains, flexible loop regions, and protein adjustments to the lipid bilayer environment. In contrast, the transmembrane core and ligand-binding pocket remained structurally stable throughout the simulation (as supported by RMSF and protein-ligand contact analyses below).^78^

The “Lig fit Lig” plot, which illustrates the ligand’s intrinsic RMSD in relation to its reference conformation, demonstrates the ligand’s internal conformational stability. The consistently low RMSD values throughout the trajectory confirm minimal internal structural deviations, indicating that **HCM-01** maintained a stable conformation within the binding site.

Root Mean Square Fluctuation (RMSF) analysis was performed to evaluate the residue-level flexibility of EAAT2 side chains upon binding to **HCM-01**. RMSF values provide insight into the dynamic behavior and mobility of individual amino acid residues throughout the simulation.^79^ According to the RMSF plot, **HCM-01** interacted with EAAT2 residues highlighted by green-colored vertical bars; these key residues exhibited minimal fluctuations (<1.6 Å) (Figure 23). This suggests that these residues maintained a relatively rigid and stable conformation in the presence of **HCM-01**, supporting the formation of stable protein–ligand interactions. In contrast, loop regions of the transporter demonstrated higher RMSF values, peaking in the range of 3–5 Å. These areas are located apart from the binding pocket and are characterized by their higher flexibility. In summary, the RMSF values support the stability of the protein–ligand complex, as the majority of protein residues displayed minimal fluctuations, while only peripheral loop regions exhibited the expected flexibility.

**Figure 23:**
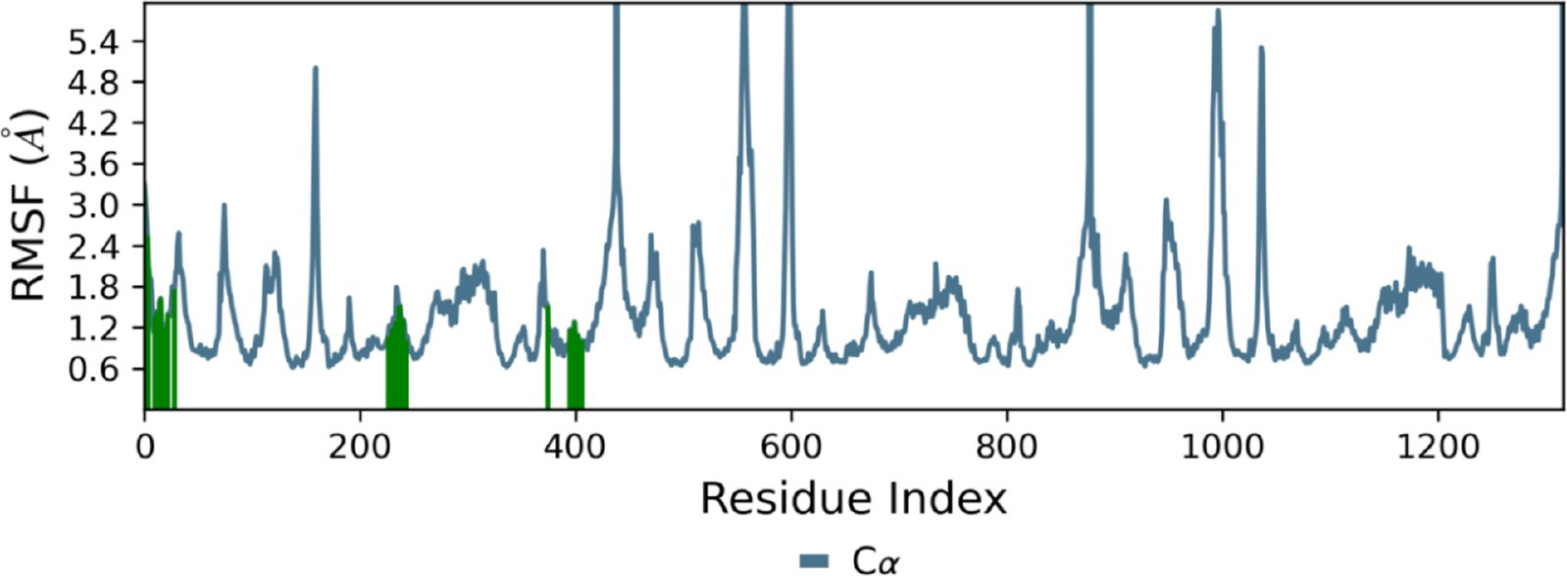
RMSF of the complex **HCM-01**-EAAT2 over the 200 ns at 300 K. Protein residues that interact with **HCM-01** are shown as green vertical bars.

In general, hydrogen bonds (H-bonds) are key interactions that allow the ligand to anchor within specific protein sites. In Figure 24, the green columns indicate the percentage of hydrogen bonds the ligand establishes with specific residues during the simulation. Certain residues, such as TRP472, show significant interactions with the ligand as they create a large percentage of hydrogen bonds. Hydrophobic interactions (purple columns) were formed with hydrophobic amino acids such as LEU, ILE, and VAL, which contributed to the stabilization of **HCM-01** within the protein’s hydrophobic pockets. Ionic interactions involving ARG residues are also evident (pink columns), facilitating binding through electrostatic attraction. Finally, water bridges (blue columns) were detected, providing adaptive stabilization between the ligand and the protein surface.

**Figure 24:**
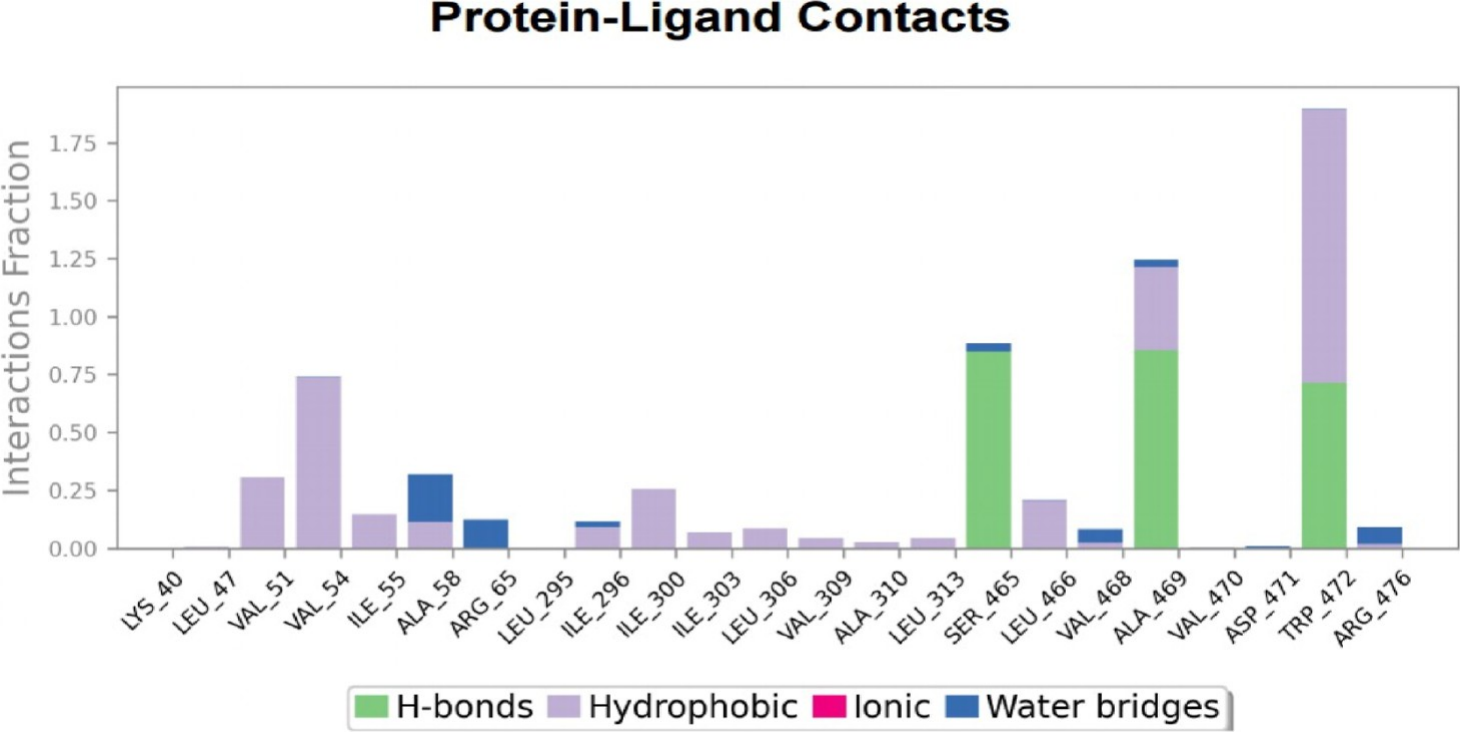
Protein–ligand contacts of **HCM-01** during the simulation.

A time-based graph illustrating the frequency and the persistence of these interactions over the period of the simulation is shown in Figure 25, while detailed ligand–atom interactions with the protein residues are presented in Figure 26. The high interaction percentages displayed in the schematic are consistent with the frequent interaction of residues like SER465, ALA469, and TRP472. Specifically, SER465 exhibits an 84% occupancy rate in hydrogen bonding, making a substantial contribution to the ligand’s interaction with the cavity. TRP472 exhibits strong and diverse interactions with the ligand, showing 70% hydrogen bonding and 35% π–π stacking interactions. The frequency of interactions throughout the simulation highlights the stability of **HCM-01** within the allosteric site.

**Figure 25:**
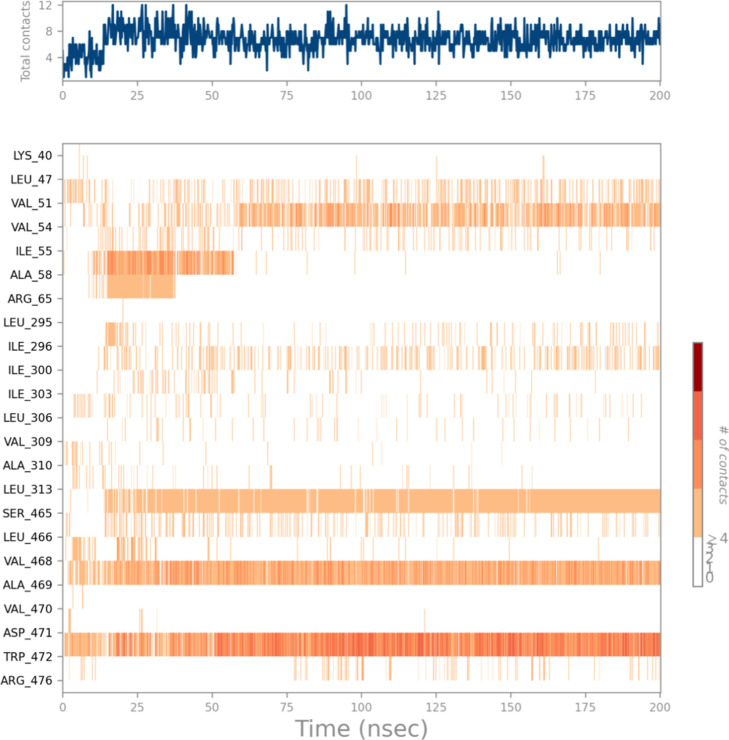
A timeline representation of the interactions and contacts (H-bonds, hydrophobic, ionic, water bridges). The top panel shows the total number of specific contacts between the protein and the ligand during the trajectory. The bottom panel shows which residues interact with the ligand in each trajectory frame. On the scale to the right of the plot, a darker shade of orange indicates that some residues make multiple distinct contacts with the ligand.

**Figure 26:**
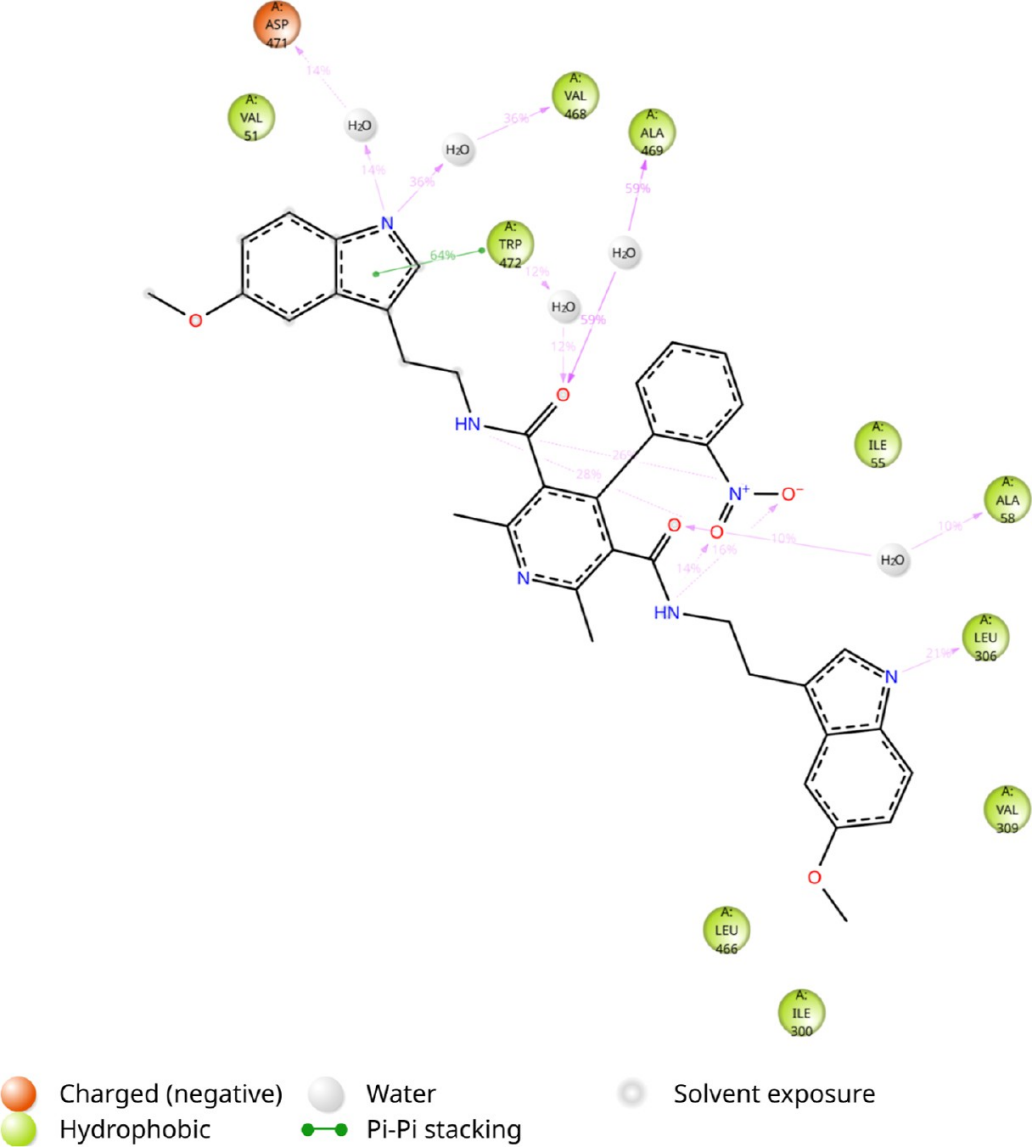
Detailed ligand–atom interactions with the protein residues that take place for more than 3.0% of the simulation time in the selected trajectory (0.00 to 200.0 ns) are displayed.

In contrast to our findings, MDS analyses for compound AK-968/15360623,^80^ revealed 20 protein–ligand interactions with EAAT2 residues, none of which were located within the allosteric pocket.

Although **HCM-01** has not yet been experimentally validated, these findings suggest that **HCM-01** has significant binding properties because it interacts directly with key residues within the allosteric pocket, indicating a favorable and specific interaction profile. Therefore, future investigations will aim to experimentally validating these molecular interactions and elucidating the underlying structure–activity relationships.

#### MM-GBSA Energies

The binding free energy of the **HCM-01**-EAAT2 complex was calculated using the Prime-MMGBSA method to evaluate the binding efficacy across three independent runs. In general, hydrophobic, hydrogen, electrostatic, and van der Waals contacts are the most common types of interactions in typical drug–protein binding and collectively influence the overall binding energy.^81^

In this study, favorable binding interactions, such as hydrogen bonds (H-bond), lipophilic (Lipo), van der Waals (vdW), and π–π packing (packing) interactions, were found to stabilize the complex, indicating the formation of a strong and energetically favorable association between **HCM-01** and EAAT2. Conversely, Coulombic, covalent, and Generalized Born electrostatic solvation (Solv GB) energies contributed unfavorably to the total binding energy, slightly weakening the overall interaction. The total binding free energy (MMGBSA dG Bind) of the **HCM-01**-EAAT2 complex was calculated to be −60.97 kcal/mol (Table 7), signifying a highly favorable and stable binding. This substantial negative energy value indicates that **HCM-01** possesses a strong affinity for EAAT2, supporting the results obtained from molecular docking and dynamic interaction analyses.

**Table 7:** Binding Free Energy (kcal mol^–1^) Calculated by the MM-GBSA Method

energy component	kcal mol^–1^
MMGBSA dG bind	–60.97
MMGBSA dG bind Coulomb	3.83
MMGBSA dG bind covalent	3.5
MMGBSA dG bind H bond	–0.25
MMGBSA dG bind Lipo	–22.19
MMGBSA dG bind packing	–7.04
MMGBSA dG bind SelfCont	–0.01
MMGBSA dG bind Solv GB	17.9
MMGBSA dG bind vdW	–56.71

## Conclusions

The compound **HCM-01** has been designed and developed to target multiple neurodegenerative pathways implicated in AD. Its neuroprotective potential was systematically evaluated using complementary in vitro, in vivo, and in silico approaches.

In vitro investigations demonstrated that **HCM-01** confers significant neuroprotection against glutamate-induced excitotoxicity in primary hippocampal neuronal cultures, where treatment resulted in a marked improvement in cell viability. In cortical and cerebellar neuronal cultures, **HCM-01** provided region-dependent functional protection within a defined concentration range. In contrast, glutamate exposure did not induce cytotoxicity in astrocytic cultures, consistent with their intrinsic neuroprotective role. Under these noncytotoxic conditions, **HCM-01** increased EAAT2 expression in astrocytes, suggesting modulation of astrocyte-mediated glutamate-handling capacity rather than direct effects on cell survival.

In parallel, TAC and TOS analyses performed in primary hippocampal neurons revealed that **HCM-01** improves oxidative/antioxidative balance under glutamate-induced stress conditions. Notably, the 1 μM concentration emerged as a convergent effective dose across experimental paradigms, coinciding with maximal neuronal viability, enhanced EAAT2 expression, and attenuation of oxidative burden. These findings support redox modulation as a complementary mechanism contributing to the neuroprotective profile of **HCM-01**.

In vivo behavioral assessment of **HCM-01** in the STZ-induced AD model indicated a trend toward improved cognitive and memory functions. Histopathological and immunohistochemical evaluations demonstrated the ability of **HCM-01** to ameliorate AD-related pathological changes by attenuating tau formation, reducing amyloidogenic processing, and partially restoring cholinergic neurotransmission, supporting the functional relevance of the in vitro findings.

Complementary MD and MDS studies supported the potential of **HCM-01** to interact with the allosteric site of the EAAT2 transporter, which plays a central role in regulating the neurotoxic glutamate neurotransmitter in the synaptic cleft. However, direct transporter activity and uptake assays will be required to validate this proposed interaction.

Taken together, in vitro and in vivo results, combined with molecular modeling data, suggest that **HCM-01** may exert neuroprotective effects through attenuation of glutamate-induced excitotoxicity, improvement of oxidative balance, and modulation of astrocyte-mediated glutamate regulation. These results support **HCM-01** as a promising multitarget preclinical candidate for further investigation in early stage and sporadic AD models.

## Materials and Methods

### Chemistry

Reagents (melatonin CAS: 73-31-4; and nifedipine CAS: 21829-25-4), catalysts, and solvents were purchased from Sigma-Aldrich and BLD Pharm and were used without further purification. Reactions were monitored via LC–MS (Thermo Fisher TSQ Series, Athena C18-WP column, Waltham, MA USA, water with 0.01% formic acid) or TLC (silica gel, Kieselgel 60, E. Merck, Germany). Proton NMR (^1^H NMR) spectra were recorded on an Advance Bruker spectrometer (Bruker, Billerica, MA, USA) at 500 MHz, while carbon-13 NMR (^13^C NMR) spectra were recorded at 125 MHz in δ/ppm.

**HCM-01** is prepared because of four synthesis steps. These steps are given below.

### Step 1: 2-(5-Methoxy-1*H*-indole-3-yl)­ethan-1-amine (**2**)

Melatonin (**1**) was heated at 90 °C for 8 h in a solution of 1.0 g (4.3 mmol) of melatonin in 40 mL of 10% H_2_SO_4_. After cooling the reaction mixture to room temperature, 20% NaOH was added until the medium became alkaline. The mixture was then washed with ethyl acetate (3 × 20 mL), and the organic phase was dried over Na_2_SO_4_. The solvent was removed under vacuum to yield 550 mg (67%) of crude product (**2**).

Yellow solid; mp 122 °C; ^1^H NMR (500 MHz, DMSO-*d*_6_): δ 10.64 (s, 1H), 7.22 (d, *J* = 8.7 Hz, 1H), 7.08 (s, 1H), 6.99 (d, *J* = 2.4 Hz, 1H), 6.71 (dd, *J* = 8.7, 2.4 Hz, 1H), 3.76 (s, 3H), 2.81 (t, *J* = 7.1 Hz, 2H), 2.72 (t, *J* = 7.1 Hz, 2H), 1.37 (s, 2H). ^13^C NMR (126 MHz, DMSO-*d*_6_): δ 152.9, 131.4, 127.7, 123.3, 112.4, 111.9, 110.9, 100.2, 55.3, 42.7, 29.6. HRMS (ESI)*m*/*z* calcd for C_11_H_15_ON [M + H]^+^: 191.11789; found, 191.11752. The spectra are available in the Supporting Information document (Figures S1–S3).

### Step 2: Dimethyl 2,6-Dimethyl-4-(2-nitrophenyl)­pyridine-3,5-dicarboxylate (**4**)

Nifedipine (**3**) (1.0 g, 2.89 mmol) was dissolved in 25 mL CH_2_Cl_2_ (dichloromethane), and 1.37 g (3.18 mmol) PIFA ((bis­(trifluoroacetoxy)­iodo)­benzene) was added. The mixture was stirred at room temperature for 12 h. The crude product was washed with water (3 × 20 mL), and the organic phase was dried over Na_2_SO_4_. The solvent was removed under vacuum, and the oxidation product (**4**) was crystallized from hexane, yielding 900 mg (90%).

Yellow solid; mp 96 °C; ^1^H NMR (500 MHz, DMSO-*d*_6_): δ 8.26 (d, *J* = 8.0 Hz, 1H), 7.80 (t, *J* = 7.5 Hz, 1H), 7.72 (t, *J* = 7.9 Hz, 1H), 7.29 (d, *J* = 7.5 Hz, 1H), 3.44 (s, 6H), 2.55 (s, 6H). ^13^C NMR (126 MHz, DMSO-*d*_6_): δ 166.6, 156.1, 146.9, 144.7, 133.9, 130.9, 130.6, 130.4, 124.5, 124.4, 52.3, 23.1. HRMS (ESI)*m*/*z* calcd for C_17_H_17_O_6_N_2_ [M + H]^+^: 345.10811; found, 345.10757. The spectra are available in the Supporting Information document (Figures S4–S6).

### Step 3: 2,6-Dimethyl-4-(2-nitrophenyl)­pyridine-3,5-dicarboxylic acid (**5**)

Dimethyl 2,6-dimethyl-4-(2-nitrophenyl)­pyridine-3,5-dicarboxylate (**4**) (900 mg, 2.61 mmol) was dissolved in 15 mL ethanol and 15 mL H_2_O, and 733 mg (13.07 mmol) KOH was added. The reaction mixture was boiled at 80 °C for 16 h. After cooling to room temperature, the solvent was removed under vacuum, and the residue was dissolved in 30 mL of water. The pH was adjusted to 2 using 0.1 M HCl, and the solution was kept at 2 °C for 12 h. The resulting crystals were filtered and dried, yielding 50 mg (66%) of product (**5**).

Beige solid; mp 265.5 °C; ^1^H NMR (500 MHz, DMSO-*d*_6_): δ 13.27 (s, 2H), 8.24 (d, *J* = 8.2 Hz, 1H), 7.79 (t, *J* = 7.5 Hz, 1H), 7.69 (t, *J* = 7.8 Hz, 1H), 7.31 (d, *J* = 7.5 Hz, 1H), 2.55 (s, 6H). ^13^C NMR (126 MHz, DMSO-*d*_6_): δ 168.0, 154.5, 147.1, 143.4, 133.7, 131.7, 131.1, 130.0, 125.8, 124.3, 23.1. HRMS (ESI)*m*/*z* calcd for C_15_H_13_O_6_N_2_ [M + H]^+^: 317.07681; found, 317.07626. The spectra are available in the Supporting Information document (Figures S7–S9).

### Step 4: *N* ^3^ *,N* ^5^-Bis­(2-(5-methoxy-1*H*-indol-3-yl)­ethyl)-2,6-dimethyl-4-(2-nitrophenyl)­pyridine-3,5-dicarboxamide (**HCM-01**)

Mixture 1: After dissolving 2,6-dimethyl-4-(2-nitrophenyl)­pyridine-3,5-dicarboxylic acid (5) in 10 mL THF (tetrahydrofuran), 550 mg (1.74 mmol), HOBt (1-hydroxybenzotriazole hydrate) 470 mg (3.488 mmol) are added and mixed for 10 min. Subsequently, 661 mg (3.48 mmol) DCC is added to the reaction mixture and stirred for 30 min.

Mixture 2: In another reaction balloon, after dissolving 661 mg (3.48 mmol) of 5-methoxytryptamine (**2**) in 10 mL THF, Et_3_N (triethylamine) 485 μL (3.48 mmol) is added and the mixture is stirred for 30 min.

The resulting mixture 2 is added to mixture 1 and stirred at room temperature for 12 h. Afterward, the DCU formed is filtered through filter paper, and the solvent is removed under vacuum. Crude product is first washed with NaHCO_3_ (3 × 30 mL); then with 5% KHSO_4_ (3 × 30 mL) and finally with water (3 × 30 mL), and the organic phase is dried over Na_2_SO_4_. The solvent of the organic phase is removed under vacuum. The resulting crude product is purified through a silica gel column with MeOH/CH_2_Cl_2_ (5:95); and a 400 mg (35%) yellow solid product **HCM-01** is obtained.

Yellow solid; mp 224.3 °C; ^1^H NMR (500 MHz, DMSO-*d*_6_): δ 10.58 (s, 2H), 7.99–7.95 (m, 3H), 7.70 (t, *J* = 7.6 Hz, 1H), 7.51 (t, *J* = 7.9 Hz, 1H), 7.37 (d, *J* = 7.4 Hz, 1H), 7.20 (d, *J* = 8.6 Hz, 2H), 6.94 (s, 2H), 6.84 (d, *J* = 2.4 Hz, 2H), 6.71 (dd, *J* = 8.7, 2.4 Hz, 2H), 3.77 (s, 6H), 3.22–3.15 (m, 4H), 2.44 (s, 7H), 2.35–2.22 (m, 5H). ^13^C NMR (126 MHz, DMSO-*d*_6_): δ 166.1, 152.9, 152.9, 147.3, 142.1, 132.8, 132.1, 131.3, 130.5, 129.5, 128.8, 127.3, 123.9, 123.0, 112.0, 111.0, 111.0, 100.0, 55.3, 24.5, 22.2. HRMS (ESI)*m*/*z* calcd for C_37_H_37_O_6_N_6_ [M + H]^+^: 661.27691; found, 661.27661. LCMS-purity: 99.21%. The spectra are available in the Supporting Information document (Figures S10–S13).

### In Vitro Assays

#### Cell Culture and Establishment of Glutamate Excitotoxicity

Newborn Sprague–Dawley rat pups were used to obtain cortical, hippocampal, and cerebellar neurons. The heads of the rats were quickly decapitated, the skulls were opened, and the cortex and hippocampus were removed and placed in HBSS solution. After macrodigestion with a scalpel, these cell communities were taken into a flask. Then trypsin/ethylenediamine tetra acetic acid (EDTA) (0.25% trypsin- 0.02% EDTA) was added for microlysis and kept in an incubator at 37 °C and 5% CO_2_ for 5 min. At the end of this time, the cells were centrifuged at 1200 rpm for 5 min by pipetting well. Fresh medium (88% NBM (Neurobasal medium, Gibco, USA), 10% FBS (Fetal bovine serum, Gibco, USA), 2% B-27 (Supplement, ThermoFisher, Germany), and 0.1% antibiotic (Penicillin- Streptomycin)) were added to the supernatant. They were then seeded on 96-well plates and incubated at 37 °C and 5% CO_2._ ^82,83^ Primary neurons were allowed to grow and develop branches in 96-well plates for 21 days. During this process, the medium was refreshed every 5 days.

The primary neuron cultures were then exposed to neurotoxic glutamate (10^–5^ M) for 5 min. Following this, the role of **HCM-01** in mitigating glutamate toxicity was assessed by applying various concentrations of **HCM-01** (0.1 μM, 1 μM, 10 μM, 100 μM, and 1000 μM) in separate wells and incubating for 24 h.

The C8-D1A (CRL-2541), an astrocyte-type cell line, was purchased from the American Type Culture Collection (USA). The cells were cultured in Dulbeco’s Modified Eagle Medium (DMEM) (11965092, Gibco, ThermoFisher Scientific, Massachusetts, ABD) supplemented with 10% Fetal Bovine Serum (FBS) (16140071, Gibco, ThermoFisher Scientific, Massachusetts, ABD) 1% Penicillin–Streptomycin, and 1% l-Glutamine. Cultures were maintained at 37 °C in a humidified atmosphere containing 5% CO_2_.

C8-D1A cells were seeded at a density of 2 × 10^4^ cells/well in 96-well plates for cell proliferation assay. And the following day, cells were treated with 10^–5^ M glutamate and 1 μM of **HCM-01** for 24 h.

Initially, astrocytes of mouse origin and neurons of mouse origin are cultured separately in 25 cm^2^ or 75 cm^2^ cell culture flasks for proliferation. The bottom of transwell systems was coated with poly-l-lysine (1 mg/mL), and neurons are seeded into the bottom of wells in a 12-well or 6-well transwell system for coculture. After 24 to 48 h of incubation to allow neuronal attachment, astrocytes/astroglial cells are seeded into the upper wells. Cells are checked after 24 h, and images are taken under a microscope using a 10× objective in bright-field mode.

l-Glutamic acid (G8415, Sigma-Aldrich) at defined concentrations was then applied to the cultures for 24 h. Following the incubation, cells are transferred to new well plates for the MTT [3-(4,5-dimethylthiazol-2-yl)-2,5-diphenyltetrazolium bromide] assay, which was performed on primary culture. And a cell proliferation assay was performed in the same 96-well plate with toxicity treatment for astrocyte culture. For a 6-well plate, 100 μL of MTT solution (475989, Sigma-Aldrich, Missouri, USA) (5 mg/mL in PBS) was added to each well; for a 12-well plate, 25 μL was added, and 96-well plate, 10 μL was used. After 2 h of incubation, the MTT solution was removed, and 1 mL (for 6-well plates) or 250 μL (for 12-well plates) or 100 μL (for 96-well plates) of DMSO was added to dissolve the formazan crystals. Once the formazan crystals were completely dissolved, absorbance was measured at 570 nm using a microplate spectrophotometer (Quant, Bad Friedrichshall, Biotek, Germany and Agilent Technologies, BioTek, Epoch).^84^

#### Western Blot

C8-D1A cells were seeded at a density of 3 × 10^5^ cells/well in 6-well plates. After 24 h, cells were treated with 10^–5^ M glutamate and 1 μM of **HCM-01**. Following 24 h of treatment, cells were washed once with PBS and lysed in 100 μL of Cell Lytic M (Sigma-Aldrich, C2978) solution per well. Cell lysis was performed using a cell scraper, and lysates were transferred to 1.5 mL microcentrifuge tubes. The lysates were centrifuged at 12,000*g* for 20 min at 4 °C. The supernatants, containing the protein fractions, were collected and stored at −80 °C until Western blot analysis.

The protein concentration of the samples was determined by the BCA (Bicinchoninic Acid) protein assay (T9300A, Takara, Kusatsu, Japan). Concentrations were equalized to 20 μg/μL and after mixing with 4× Laemmli buffer containing β-mercaptoethanol, each sample was denatured at 95 °C for 5 min. And the proteins were loaded into 4–20% Mini-PROTEAN TGX Stain-Free Protein Gels (4568094, BioRad). Subsequently, the proteins were separated by SDS-polyacrylamide gel electrophoresis (SDS-PAGE, Criterion Cell and PowerPac Basic Power Supply, 1656019, BioRad) at 120 V, 400 A for 60 min. The proteins were transferred onto a 0.2 μm PVDF transfer membrane (17001919, BioRad) using the Trans-Blot Turbo Transfer System (1704150, BioRad). Membranes were blocked with 5% nonfat dry milk in Tris Buffered Saline containing 0.2% Tween-20 (TBST) for 30 min at room temperature. Membranes were incubated with primary antibodies, overnight at 4 °C with anti-EAAT2 (1:1000; E3P5K, Cell Signaling, Massachusetts, USA) and then incubated for 1 h at room temperature with anti-GAPDH (1:20.000; ab181602, Abcam, Cambridge, UK) as a control. Goat antirabbit IgG H&L (1:2000; ab96899, Abcam, Cambridge, UK) was used as a secondary antibody. Protein bands were visualized using enhanced chemiluminescence (ECL, 1705061, BioRad) reagents and imaged with a ChemiDoc (Bio-Rad). EAAT2 protein band intensities were quantified using ImageJ software and normalized to GAPDH levels. The experiment was repeated three times independently, and data were presented as the mean ± standard deviation (SD).

#### TAC and TOS

The presence of oxidative stress in primary hippocampal neuronal cultures was detected by determining the level of TAC, which inhibits the formation of the 2,2′-azinobis­(3-ethylbenzothiazoline-6-sulfonate (ABTS) radical cation through antioxidants. These assays provide a general measure of the cellular redox state. The analysis of total antioxidant levels was performed using the TAC kit (E-BC-K801-M, Elabscience) on the lysate obtained from the cells after drug exposure for 24 h. The results obtained were analyzed using the SPSS program.

### In Vivo Alzheimer’s Rat Model

A total of 56 female Sprague–Dawley rats were used for the STZ-induced Alzheimer’s rat model, divided into 7 groups of 8 animals each. Female Sprague–Dawley rats were used because AD shows a higher prevalence and faster progression in women, making females a more clinically relevant model for this pathology.^85^

The timeline for behavioral tests is represented in Table 8.

- Group 1: Control group. No surgical procedure or intracerebroventricular injection was performed in this group.
- Group 2 (Sham operation): This group underwent the same surgical procedure and received bilateral intracerebroventricular injections of a pH 4.5 citrate buffer (vehicle) but did not receive streptozotocin.
- Group 3 (STZ): Streptozotocin group (disease group). This group received bilateral ICV injections of streptozotocin on the first day.
- Group 4 (STZ + **MEM**): Streptozotocin + memantine group. Following streptozotocin injection, this group received memantine treatment (10 mg/kg) for 24 days.^86^
- Group 5 (STZ + **HCM**-**01**): Streptozotocin + HCM group. After streptozotocin injection, this group was treated with **HCM-01** (50 mg/kg) orally for 24 days.
- Group 6 (STZ + **HCM**-**01**): Streptozotocin + HCM group. After streptozotocin injection, this group was treated with **HCM-01** (50 mg/kg) I.P. for 24 days.
- Group 7 (STZ + **HCM**-**01**): Streptozotocin + HCM group. After streptozotocin injection, this group was treated with **HCM-01** (100 mg/kg) orally for 24 days.

**Table 8:** Timeline of Behavioral Studies

1 day	19–20 day	21 day	39 day	40–41 day	42–46 days	46 day
STZ treatment	passive avoidance test pretreatment measurement	drug treatment started	locomotor activity test	passive avoidance test posttreatment measurement	Morris water maze test	sacrifice, tissue and were taken

The animals were divided into groups in a double-blind manner based on the behavioral test results obtained on the 21st day. On day 0 of treatment, injections were administered intraperitoneally (I.P.) to the control, STZ, STZ + MEM, and STZ + HCM groups.

The control group did not receive any treatment, while the sham-operated group received a citrate buffer as the vehicle. The STZ + MEM group received memantine orally at 10 mg/kg, and the STZ + HCM groups received **HCM-01** at doses of 50 and 100 mg/kg. Due to the high concentration of the drug, it was administered only at 50 mg/kg I.P. These injections continued for 24 days until the animals were sacrificed on the 46th day. Two small holes were drilled in the skull, large enough for the STZ injection needle to pass through and penetrate the brain surface. Following the coordinates measured from the bregma, the lateral ventricles were reached: −0.8 mm on the anteroposterior axis, ±1.4 mm on the mediolateral axis, and −3.6 mm on the dorsoventral axis.^87^

The coordinates for ICV injections were determined using the rat brain atlas. The coordinates for both ventricles were determined bilaterally as follows: 0.8 mm anteroposterior, 1.5 mm mediolateral, and 3.6 mm dorsoventral. On the first day of the experiment, after ensuring that the rats were immobilized securely in the stereotaxic apparatus, anesthesia was induced with a ketamine/xylazine (100/10 mg/kg) dose to prevent head movement. Following immobilization, the area where the incision would be made was cleaned with a 10% povidone-iodine solution. An incision was then made with a sterile scalpel blade at the cleaned area. After the incision, the coordinates determined using the rat brain atlas were marked on the cleaned area, and the marked regions were gently drilled with a dental drill to avoid damaging the brain surface.

The brain membranes in the drilled regions were lifted using a fine needle, and a volume of 10 μL of STZ solution, prepared and filled into a Hamilton syringe, was injected bilaterally into the ventricles at a dose of 3 mg per kilogram.^87^

The pH 4.5 citrate buffer was injected into the sham-operated group at the specified coordinates. After the procedures, the incision site was sutured using a 4/0 thread. A waiting period of 21 days was allowed for the development of the AD-like phenotype. On days 19–20 after STZ induction and before randomization, pretreatment screening tests, including the Passive Avoidance Test, were performed. Animals that did not show a decline in memory performance (i.e., those in whom the AD-like phenotype did not develop) were excluded from the study, and the remaining animals were randomized under double-blind conditions on day 21. Drug treatments were initiated on day 21 according to the respective group protocols and continued for 24 days. Locomotor activity tests were performed on day 39, and post-treatment Passive Avoidance Tests were conducted on days 40–41. The Morris Water Maze test was performed between days 42 and 46. Finally, on day 46, the animals were sacrificed, and tissue samples were collected for further analyses.

#### Passive Avoidance Test

A passive (step-through) avoidance apparatus consisting of two adjoining compartments, a light chamber and a dark chamber, was used (MAY two-compartment Passive Avoidance; Commat Ltd.), Ankara, Turkey; internal dimensions per chamber (25 × 25 × 25 cm). The compartments were separated by an automated guillotine door. A top-mounted camera captured behavior, and image acquisition/timing and latency extraction were performed in EthoVision XT (Noldus Information Technology, Wageningen, The Netherlands). The floor of the dark chamber consisted of parallel stainless-steel grid rods (diameter [3 mm], spacing [10 mm]) connected to a constant-current shock generator.

On the first day of the experiment, each rat was placed in the light chamber. Following a 10 s acclimatization period, the door opened automatically. Upon full entry into the dark chamber (all four paws), the door closed, and a single foot-shock (1.0 mA for 3 s) was delivered via the dark-chamber grid floor. The rat was then removed and returned to its home cage. This single, brief reinforcement (1.0 mA/3 s) was selected a priori to secure robust long delay retention, while minimizing exposure; shock output was verified before sessions, animals were monitored post-trial, and independent open-field locomotor analyses confirmed no group-wise motor impairment.^88,89^

The Passive Avoidance Test was performed as both a pretreatment and post-treatment assessment. Retention was evaluated 24 ± 2 h after the training session at each measurement period. During the retention test, rats were placed in the light compartment, and the door was opened as in the training session; no electrical shock was delivered. The step-through latency (s) to enter the dark compartment was recorded with a cutoff time of 300 s, and animals that did not enter the dark chamber within this period were assigned a latency value of 300 s.

The first assessment was performed before treatment initiation (days 19–20), while the second assessment was conducted after 24 days of treatment (days 40–41).

For graphical presentation, the results obtained on the first and second retention days were used. All test sessions were carried out during the light phase within a fixed time interval. The chambers were cleaned with 70% ethanol and dried between trials to prevent odor interference.

The Passive Avoidance Test evaluates aversive associative memory, which represents a form of inhibitory avoidance learning. Higher step-through latency values indicate better memory performance. Each assessment consisted of one training session followed by one retention session. The learning criterion was defined as reaching the maximum latency of 300 s, and no animals were excluded based on latency values. Data were presented as mean ± standard deviation.^90,91^

#### Morris Water Maze Test

The MWM in this study was used to assess hippocampus-dependent spatial learning (acquisition), operationalized as a progressive reduction in escape latency across training days. Spatial learning was assessed in a circular black pool (diameter 1.5 m, wall height 0.6 m) filled to a depth of approximately 42 cm. Water temperature was maintained at 25 ± 1 °C and rendered opaque with a nontoxic opacifier to prevent visibility of the submerged escape platform (12.5 × 12.5 cm, fixed location in a single target quadrant, 1–2 cm below the surface). An overhead camera linked to tracking software recorded swim paths. Stable extra-maze visual cues (geometric shapes) were mounted on the surrounding walls and remained constant throughout acquisition; cardinal directions were defined within the software (north, south, east, west).^90^

Acquisition training (multitrial/day design). Each rat completed four trials per day for four consecutive days. Within each day, trials began from the four distinct start positions (north, south, east, west) in pseudorandom order; the platform location did not change across trials or days. Maximum trial duration was 90 s. If the platform was not located within this period, the rat was gently guided to the platform and allowed a brief 20–30 s rest on it before the next trial. Escape latency (s) was recorded for every trial. For statistical analyses and plotting, trial-level data were averaged within day to yield one daily value per animal, thereby capturing within-day learning while minimizing fatigue and simplifying longitudinal comparisons across days. The primary outcome was daily mean escape latency (s) across days 1–4, expressed as the mean.^90^

#### Locomotor Activity Test

To preclude motor confounds in the interpretation of memory outcomes, spontaneous locomotion was quantified in computer-linked open-field activity cages equipped with intersecting infrared beams (MAY Act series; Commat Ltd., Ankara, Turkey). Each rat was tested individually in a transparent, square acrylic arena under constant illumination for a single 10 min session. Tests were conducted during the light phase at a fixed time of day; animals were habituated to the testing room for at least 30 min beforehand. Between trials, arenas were cleaned with 70% ethanol and thoroughly dried to minimize olfactory cues and carry-over effects. Data were acquired using the manufacturer’s software at the native sampling rate.^90^ The prespecified primary end points were total distance (cm) and mean speed (cm/s; distance divided by session duration), with resting percentage serving as the immobility index (caclulated according to the manufacturer’s default immobility threshold). The data were analyzed using one-way analysis of variance (ANOVA), with separate analyses conducted for each end point, followed by Tukey’s HSD pairwise comparisons.

#### Histopathological Examinations

The brain tissues of the rats were fixed in a 10% neutral buffered formalin solution after necropsy. Tissues were then processed through routine alcohol-xylene steps and embedded in paraffin blocks. Sections of 4 μm thickness were cut onto poly lysine-coated slides and stained with hematoxylin–eosin. The observed pyknotic and degenerative changes in the neurons of the cornu Ammonis (CA1/CA2, CA3) regions were semiquantitatively evaluated as absent (−), mild (+, 1–2 pyknotic and degenerative cell), moderate (++, 3–5 pyknotic and degenerative cells), and severe (+++, 6> pyknotic and degenerative cells), by examining six different areas from six sections of each specimen.

#### Immunohistochemical Examinations

Sections of 4 μm thickness, mounted on poly lysine-coated slides, were deparaffinized using a xylene and alcohol series, followed by washing with PBS. Subsequently, endogenous peroxidase activity was blocked by incubating the tissues in 3% H_2_O_2_ for 10 min. Antigen retrieval was performed by treating the sections with antigen retrieval solution for two cycles of 5 min each at 500 W. Afterward, sections were incubated overnight at +4 °C with primary antibodies against β-amyloid (Santa Cruz, Catalog no. sc-28365, dilution 1/100), Tau (Santa Cruz, Catalog no. sc-390476, dilution 1/2000), and AChE (Santa Cruz, Catalog no. sc-373901, dilution 1/100). For secondary detection, the Large Volume Detection System: anti-Polyvalent, HRP (ThermoFisher, Catalog no: TP-125-HL) was applied according to the manufacturer’s recommendations. AEC (3-Amino-9-Ethylcarbazole) was used as the chromogen. Counterstaining was performed with Mayer’s Hematoxylin, and then the sections were coverslipped with an aqueous-based mounting medium for examination under a light microscope. Immunopositivity in the cornu ammonis (CA1/CA2, CA3) regions was semiquantitatively evaluated as absent (−), mild (+, <10% immunopositive area), moderate (++, 11–20% immunopositive area), severe (+++, 21–30% immunopositive area), and very severe (++++, 31>% immunopositive area) by examining six different areas from six sections of each specimen.

#### Measurement of **HCM-01** in Brain Tissues

Brain tissues were homogenized using a bead homogenizer to ensure uniform disruption. The homogenates were centrifuged at 10,000 rpm to remove large particles, and the supernatant was collected. Methanol was added to the supernatant to precipitate residual proteins, followed by additional centrifugation. The clarified samples were subjected to QTOF-HRMS mass spectrometric analysis, and the obtained spectra were compared with those of the standard reference group.

### In Silico Studies

#### Molecular Docking (MD)

The computational study was performed on a computer system consisting of a DELL Intel­(R) Core (TM) i9-13900 HX CPU @ 2.20 GHz processor with 32.0 GB RAM and a 64 bit operating system. The Schrödinger Small-Molecule Drug Discovery Suite (2021-4, Schrödinger, LLC, New York, NY, 2021) was utilized. The Schrödinger software’s Ligprep module was used to create the 3D coordinates for **HCM-01**. OPLS4 force field was used to calculate tautomers, partial atomic charges, and ionization states at pH 7.0.^92^

The crystal structure of EAAT2 (PDB ID: 7XR4)^93^ was downloaded from the Protein Data Bank^94^ based on specific parameters such as resolution (3.40 Å). The Protein Preparation Wizard tool^95^ was used to prepare the protein structure, which included adding hydrogen atoms, filling in the empty loops using Prime, and removing water molecules from the crystallographic structure. At a pH of 7.0, PROPKA was used to evaluate the protonation status of ionizable protein groups. After assigning charges to each atom, the structure underwent energy minimization and refinement using the OPLS4 force field.

Glide^96^ and IFD^97^ modules were used for rigid and flexible docking, respectively. One dependable and efficient docking method for considering flexibility in both ligands and the binding pocket residues of target transporters is the IFD module of the Maestro molecular modeling package.^97^ Glide/SP (Standard Precision) was performed for **HCM-01** with both rigid and flexible docking.

The grid box for the allosteric site within the EAAT2 transporter was identified based on literature refs 76 and 98 which indicated the involvement of key residues including Lys299, Ala362, Asp471, Trp472, Asp475, Arg476, Thr479, Leu295, and Asp485.

#### Molecular Dynamics Simulation (MDS)

Following these investigations, the Desmond module^99^ from the Schrödinger suite was used to perform a 200 ns MDS. Given the presence of the 7XR4 cell membrane protein, we incorporated aqueous environment modeling alongside all three protein chains and the cell membrane model. Prior to simulation, the complexes underwent preprocessing, optimization, and minimization steps. The OPLS3e force field was used during the minimization process.^100^ To create the simulation system, the System Builder Tool was employed. The transferable intermolecular potential three-point (TIP3P) solvent model was added, and an orthorhombic box with dimensions of 10 × 10 × 10 Å was employed. The cell membrane was modeled using POPC parameters. The neutralization of the model was conducted by the addition of counterions when needed. Additionally, 0.15 M NaCl was included to mimic the physiological state. The simulations were carried out under NPT (Isothermal–Isobaric) ensemble conditions, with a pressure of 1 atm and a temperature of 300 K. Before the initiation of the simulation, the complex was allowed to relax. Trajectories were saved at intervals of 200 ps for subsequent analysis of the simulation results.

#### Free Energy Calculation by Prime-MM/GBSA

The free energy of protein–ligand complexes was determined using molecular mechanics force fields and implicit solvation, employing the Prime-MM/GBSA module^101^ of Schrödinger Suite. Briefly, the protein–ligand pose was energy minimized using the local optimization feature of Prime, and binding free energies were calculated using the MM/GBSA continuum solvent model encompassing the OPLS3e force field,^100^ VSGB solvent model,^102^ and the rotamer search algorithm.^103^

#### Calculations and Statistical Analysis

Behavioral and biochemical data were analyzed in GraphPad Prism v10.4 (GraphPad Software, San Diego, CA, USA). For each variable, normality was assessed using the Shapiro–Wilk test (with Q–Q plot inspection), and homogeneity of variances using Levene’s test. Parametric data were analyzed using one-way ANOVA. Tukey and LSD tests were then used for posthoc comparisons between all pairs. End points exhibiting non-normality were analyzed using the Kruskal–Wallis test with Mann–Whitney *U* test. MWM escape latency data were analyzed using two-way repeated-measures ANOVA (Group × Day).

The histopathological and immunohistochemical findings were analyzed using SPSS 20.00 (IBM Corp., Armonk, NY, USA) software. The difference between groups was assessed using nonparametric tests, specifically the Kruskal–Wallis test for overall differences among groups, followed by the Mann–Whitney *U* test to identify the specific groups contributing to the differences (*p* < 0.05). Results are expressed as mean ± SD.

### Ethical Statement

Ethic Committee: Local Ethics Council of Animal Experiments (Atatürk University-HADYEK); Number: 42190979-050.01.04-E.2100074699; Date: 10.03.2021.

## Supplementary Material



## Notes

Spectroscopic and histological data supporting compound characterization and biological findings. Figures S1–S13 present the ^1^H NMR, ^13^C NMR, HRMS, and LC–MS spectra of compounds (2), (4), (5), and **HCM-01**. Figure S14 shows Western blot images of EAAT2 expression. Tables S1–S4 summarize the neuronal degeneration analysis and immunohistochemical staining for Tau, β-amyloid, and AChE (PDF)
