# Supplementary material for: Multi-Target Neuroprotective Compound Exhibits EAAT2-Modulating and Alzheimer’s Pathology–Attenuating Effects in In Vitro and In Vivo Models
Source: ACS Chem Neurosci. 2026 Apr 29;17(10):1919–44. doi: 10.1021/acschemneuro.5c00873 (PMC13195668; doi:10.1021/acschemneuro.5c00873)
Supplement: Supplementary file 1 [file cn5c00873_si_001.pdf]

Multi-Target Neuroprotective Compound Exhibits EAAT2-Modulating and Alzheimer's Pathology–Attenuating Effects in In Vitro and In Vivo Models

Ahmet Hacimuftuoglu [a*], Nurullah Saracoglu [b,c], Sana Saffour [d], Nadeem Abad [d], Yunus Kesgun [d], Nadjiba Zegheb [d], Ersin Gundeger [e], Fatma Yesilyurt [a], Merve Nur Ataş [d], Gizem Bati-Ayaz [d], Öznur Altunlu [a], Burak Çınar [a], Mehmet Ali Yörük [a], Ufuk Okkay [a], Mustafa Özkaraca [f], Orhan Ates [g], Ali Taghizadehghalehjoughi [h], Ferruh Lafzi [b], Hasan Türkez [i]

[a]Department of Medical Pharmacology, Faculty of Medicine, Atatürk University, 25240, Erzurum, Türkiye

[b]Department of Chemistry, Faculty of Sciences, Atatürk University, 25240, Erzurum, Türkiye

[c]Biotechnology Institute, Ankara University, 06135, Ankara, Türkiye

[d]Trustlife Labs Drug Research & Development Center, 34774, İstanbul, Türkiye

[e]Biochemistry Department, Faculty of Pharmacy, Istanbul Health and Technology University, Sütlüce, Beyoğlu, 34275, İstanbul, Türkiye

[f]Faculty of Veterinary Medicine, Department of Pathology, Cumhuriyet University, Sivas, Turkey

[g]Department of Eye Diseases, Faculty of Medicine, Atatürk University, 25240, Erzurum, Türkiye

[h]Department of Medical Pharmacology, Faculty of Medicine, Bilecik Seyh Edebali University, 11230 Bilecik, Türkiye

[i]Department of Medical Biology, Faculty of Medicine, Atatürk University, 25240, Erzurum, Türkiye

Table of Figures

Figure S1: ^{1}H-NMR spectrum of compound (2) S3
Figure S2: ^{13}C-NMR spectrum of compound (2) S3
Figure S3: HRMS spectrum of compound (2) S4
Figure S4: ^{1}H-NMR spectrum of compound (4) S5
Figure S5: ^{13}C-NMR spectrum of compound (4) S5
Figure S6: HRMS spectrum of compound (4) S6
Figure S7: ^{1}H-NMR spectrum of compound (5) S7
Figure S8: ^{13}C-NMR spectrum of compound (5) S7
Figure S9: HRMS spectrum of compound (5) S8
Figure S10: ^{1}H-NMR spectrum of HCM-01 .... S9
Figure S11: ^{13}C-NMR spectrum of HCM-01 .... S9
Figure S12: HRMS spectrum of HCM-01 .... S10
Figure S13:LCMS spectrum of HCM-01 .... S11
Figure S14: Western Blot immunoblot images of EAAT2 expression S12

Table of Tables

Table S1. Statistical analysis of degenerative changes observed in neurons. S12
Table S2. Immunohistochemical staining of Tau. S13
Table S3. Immunohistochemical staining with β-Amyloid. S13
Table S4. Immunohistochemical staining with AChE. S13

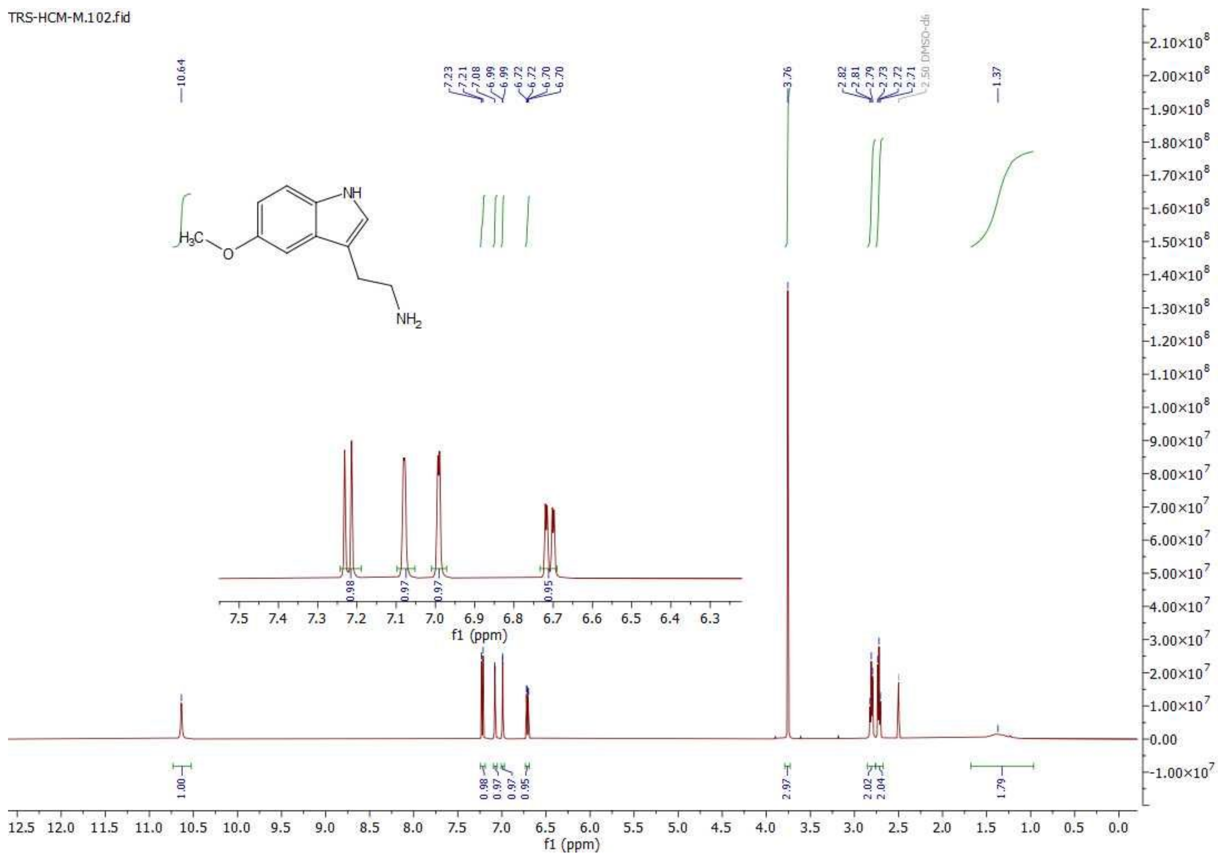

Figure S1: ^{1}H-NMR spectrum of compound (2)

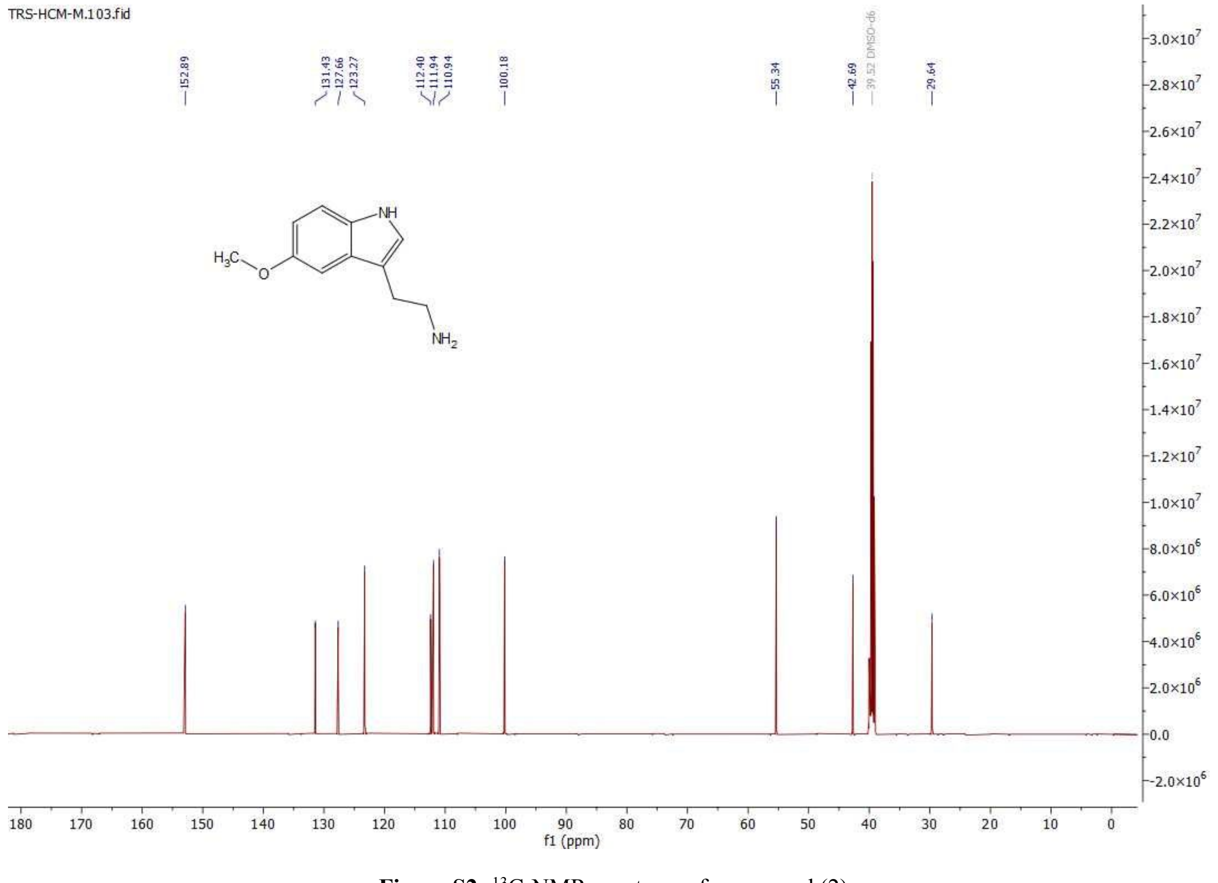

Figure S2: ^{13}C-NMR spectrum of compound (2)

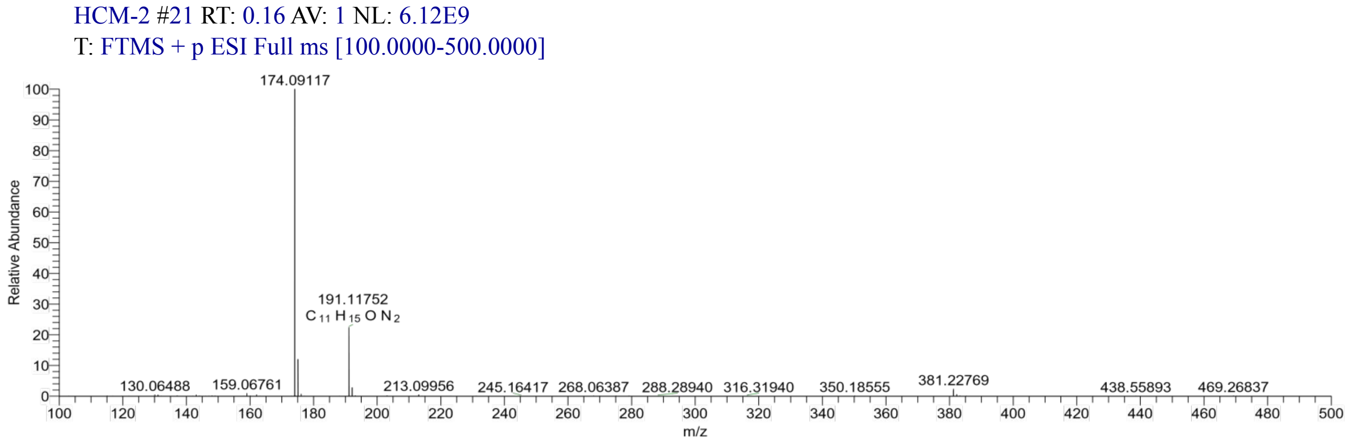

HCM-M#21 RT: 0.16 SM: 7G
T: FTMS + p ESI Full ms [100.0000-500.0000]

m/z	Intensity	Relative	Theo. Mass	Delta (ppm)	RDB equiv.	Composition
131.07271	23488050.0	0.36				
137.13223	8137748.5	0.12				
143.07283	23754112.0	0.36				
148.07553	9037323.0	0.14				
159.06761	55212428.0	0.85				
162.09108	27288442.0	0.42				
174.02744	8506340.0	0.13				
174.09117	6528799744.0	100.00				
174.15056	10665929.0	0.16				
175.09433	779257024.0	11.94				
176.09750	41097324.0	0.63				
191.11752	1450855552.0	22.22	191.11789	-1.92	5.5	$C_{11}H_{15}ON_2$
192.12099	171043120.0	2.62				
193.12450	9149948.0	0.14				

Figure S3: HRMS spectrum of compound (2)

^{1}H NMR (500 MHz, DMSO-*d_6*) δ 10.64 (s, 1H), 7.22 (d, *J* = 8.7 Hz, 1H), 7.08 (s, 1H), 6.99 (d, *J* = 2.4 Hz, 1H), 6.71 (dd, *J* = 8.7, 2.4 Hz, 1H), 3.76 (s, 3H), 2.81 (t, *J* = 7.1 Hz, 2H), 2.72 (t, *J* = 7.1 Hz, 2H), 1.37 (s, 2H). **^{13}C NMR (126 MHz, DMSO-*d_6*)** δ 152.9, 131.4, 127.7, 123.3, 112.4, 111.9, 110.9, 100.2, 55.3, 42.7, 29.6. **HRMS (ESI) *m/z*** calcd for $C_{11}H_{15}ON$ $[M+H]^+$: **191.11789**; found: **191.11752**.

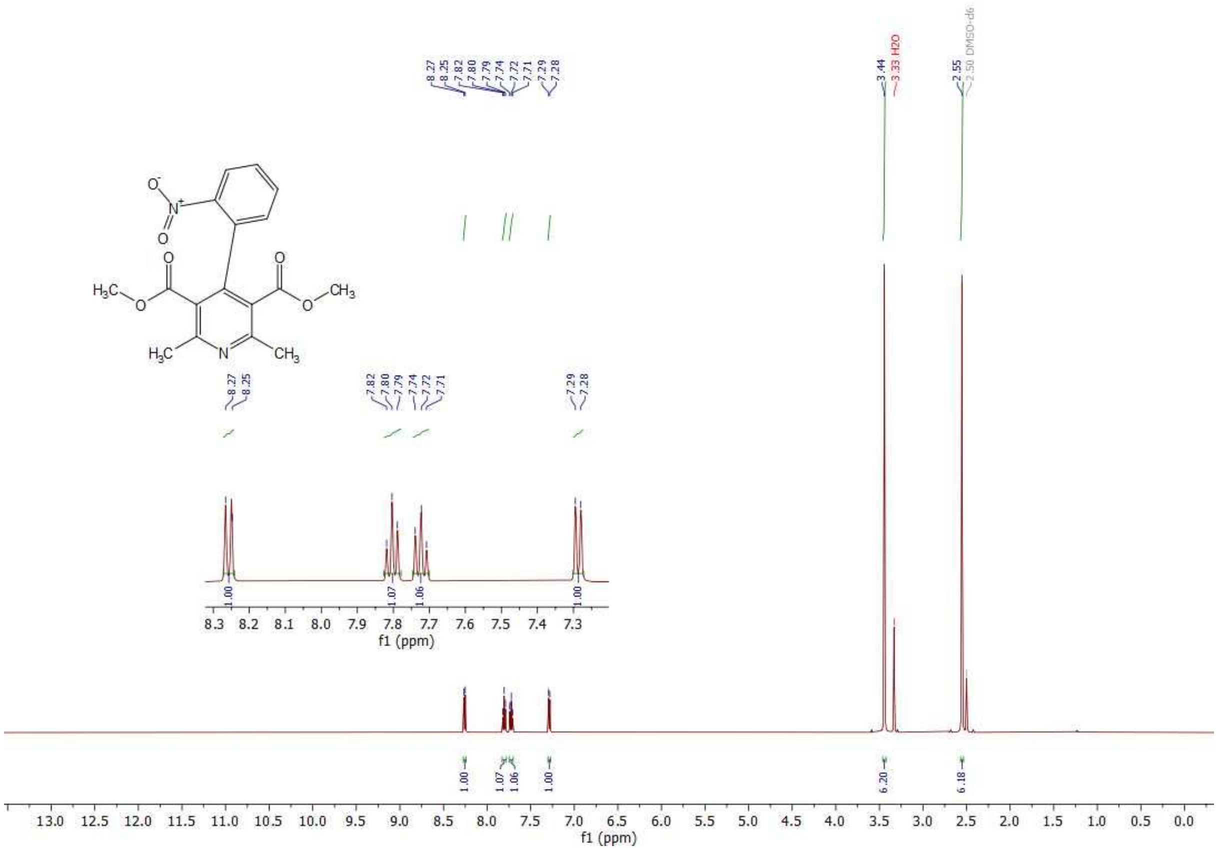

Figure S4: ^{1}H-NMR spectrum of compound (4)

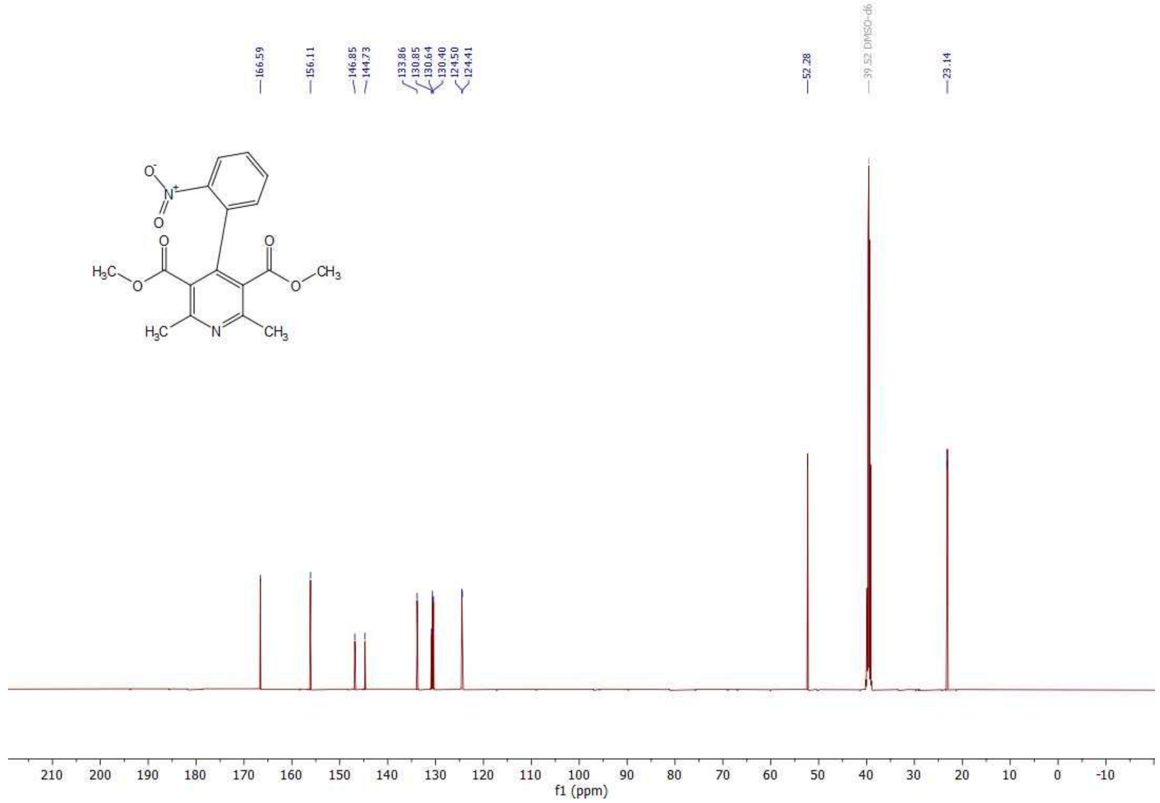

Figure S5: ^{13}C-NMR spectrum of compound (4)

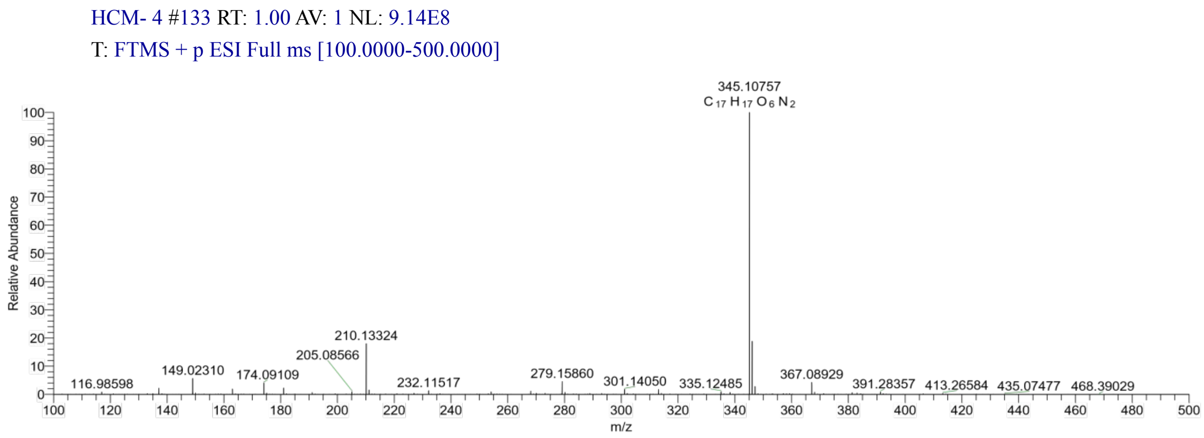

HCM-S1#17 RT: 0.12 SM: 7G
T: FTMS + p ESI Full ms [100.0000-500.0000]

m/z	Intensity	Relative	Theo. Mass	Delta (ppm)	RDB equiv.	Composition
210.13310	24467356.0	0.27				
256.09464	9156029.0	0.10	256.09414	1.93	5.0	$C_{12}H_{16}O_6$
284.08926	20087162.0	0.22				
345.10757	9169178624.0	100.00	345.10811	-1.56	10.5	$C_{17}H_{17}O_6N_2$
346.11060	1742119808.0	19.00				
347.11285	248328896.0	2.71				
348.11621	26667322.0	0.29				
357.10825	9222507.0	0.10				
359.08731	14232158.0	0.16				
367.08929	294679680.0	3.21				
368.09235	53954892.0	0.59				
369.09357	9298507.0	0.10				
373.10248	22943612.0	0.25				
375.11780	16743730.0	0.18				

Figure S6: HRMS spectrum of compound (4)

^{1}H NMR (500 MHz, DMSO-*d_6*) δ 8.26 (d, *J* = 8.0 Hz, 1H), 7.80 (t, *J* = 7.5 Hz, 1H), 7.72 (t, *J* = 7.9 Hz, 1H), 7.29 (d, *J* = 7.5 Hz, 1H), 3.44 (s, 6H), 2.55 (s, 6H). **^{13}C NMR (126 MHz, DMSO-*d_6*)** δ 166.6, 156.1, 146.9, 144.7, 133.9, 130.9, 130.6, 130.4, 124.5, 124.4, 52.3, 23.1. **HRMS (ESI) *m/z*** calcd for $C_{17}H_{17}O_6N_2$ $[M+H]^+$: **345.10811**; found: **345.10757**.

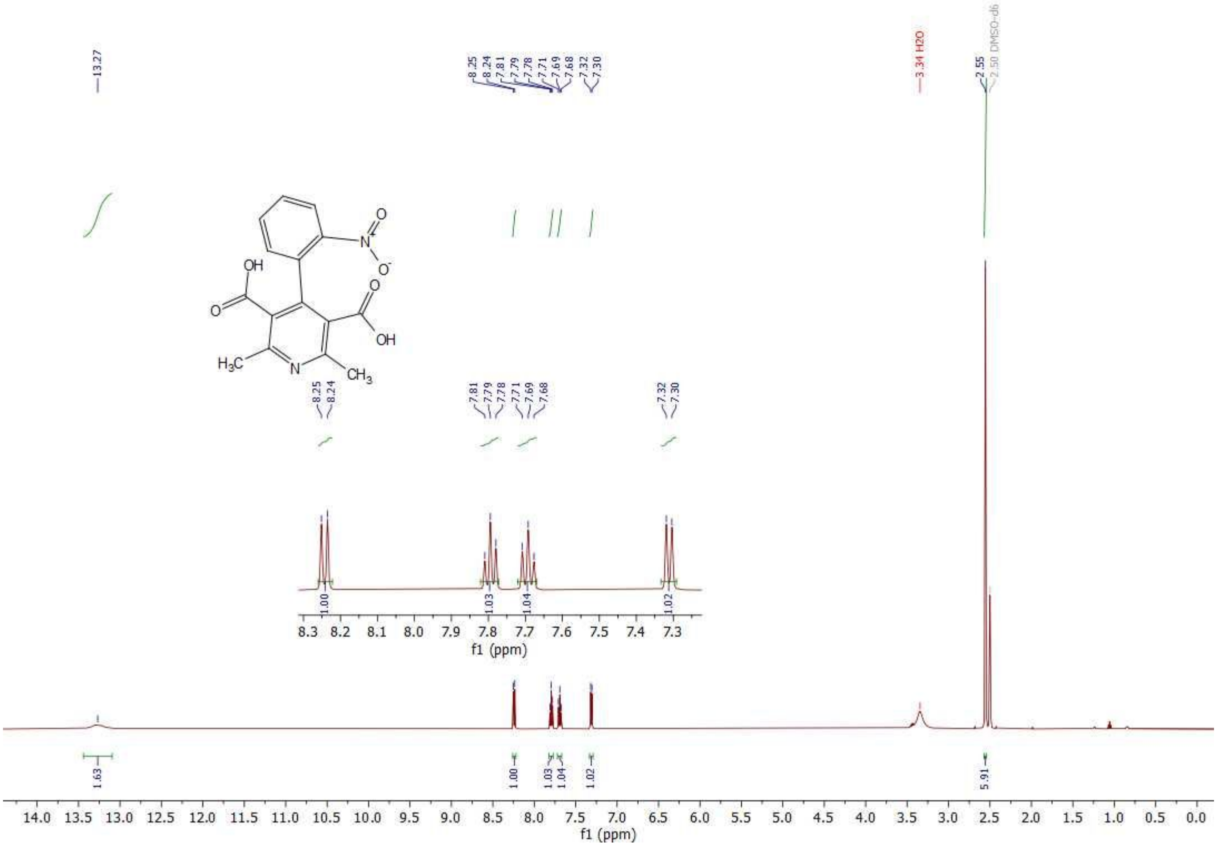

Figure S7: ^{1}H-NMR spectrum of compound (5)

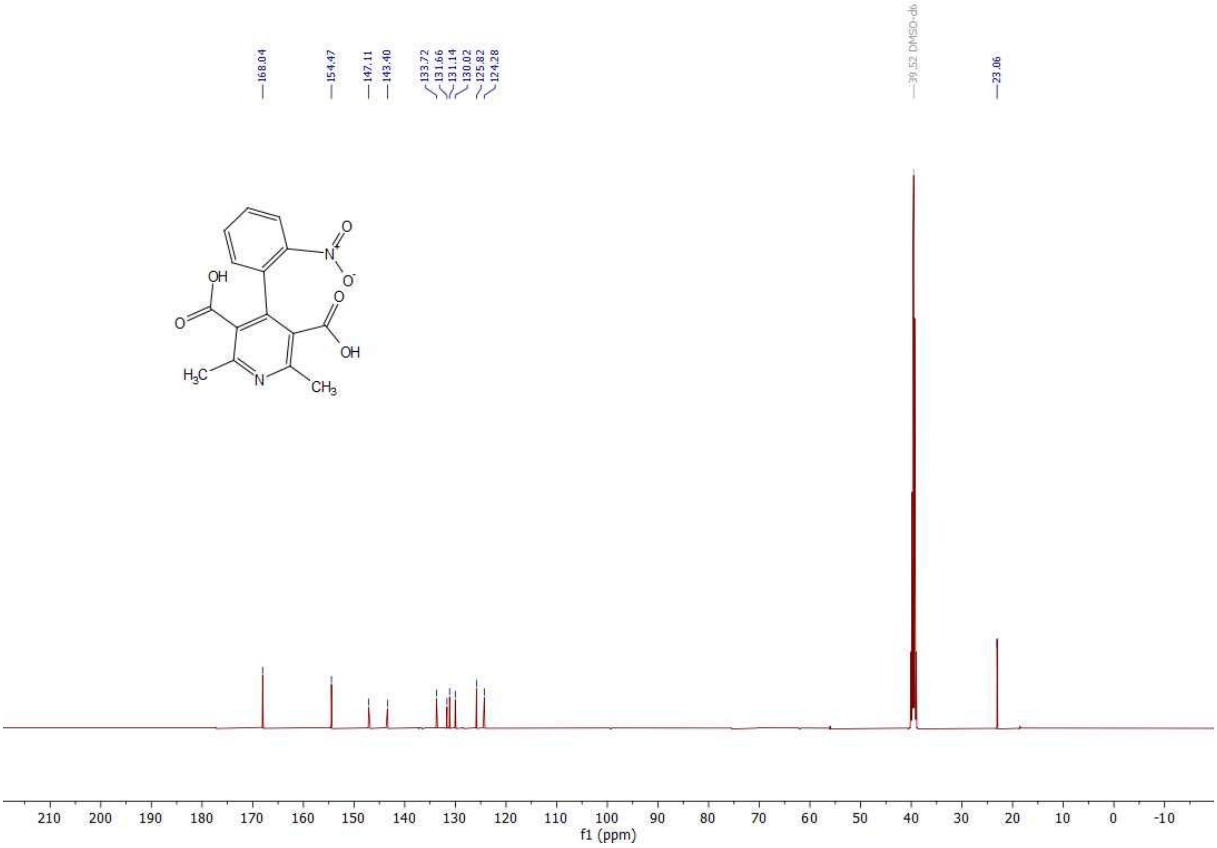

Figure S8: ^{13}C-NMR spectrum of compound (5)

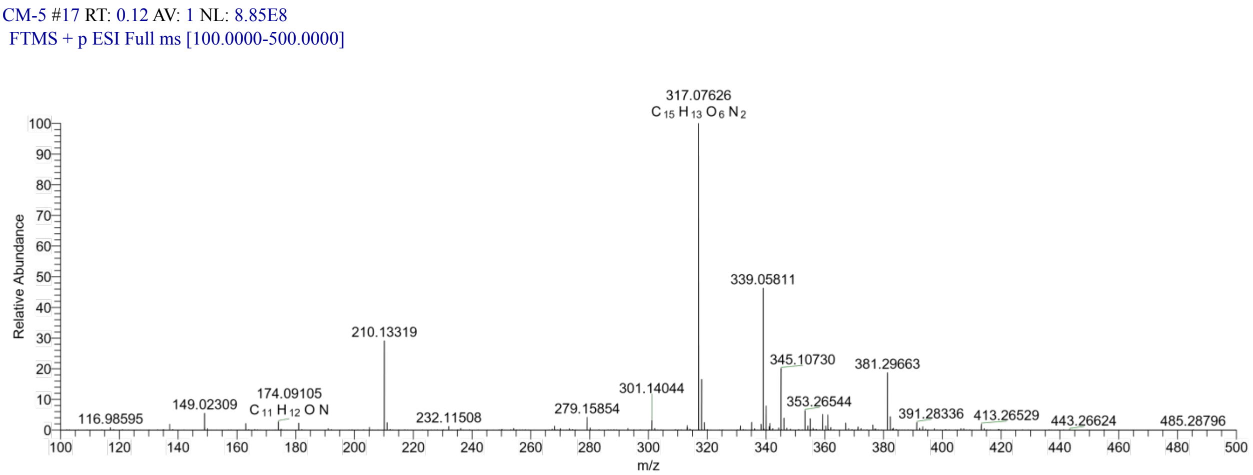

HCM-S2#17 RT: 0.12 SM: 7G
T: FTMS + p ESI Full ms [100.0000-500.0000]

m/z	Intensity	Relative	Theo. Mass	Delta (ppm)	RDB equiv.	Composition
149.02309	50130188.0	5.52				
174.09105	25751016.0	2.83	174.09134	-1.67	6.5	C11 H12 O N
210.13319	257956560.0	28.38				
279.15854	37200528.0	4.09				
301.14044	29888998.0	3.29				
317.07626	908963072.0	100.00	317.07681	-1.73	10.5	C15 H13 O6 N2
318.07953	146862656.0	16.16				
319.08160	22493134.0	2.47				
335.12466	23057312.0	2.54				
339.05811	435218592.0	47.88				
340.06140	73212016.0	8.05				
345.10730	181949008.0	20.02				
346.11078	34449320.0	3.79				
353.26544	59181300.0	6.51				

Figure S9: HRMS spectrum of compound (5)

^{1}H NMR (500 MHz, DMSO-*d_6*) δ 13.27 (s, 2H), 8.24 (d, *J* = 8.2 Hz, 1H), 7.79 (t, *J* = 7.5 Hz, 1H), 7.69 (t, *J* = 7.8 Hz, 1H), 7.31 (d, *J* = 7.5 Hz, 1H), 2.55 (s, 6H). **^{13}C NMR (126 MHz, DMSO-*d_6*)** δ 168.0, 154.5, 147.1, 143.4, 133.7, 131.7, 131.1, 130.0, 125.8, 124.3, 23.1. **HRMS (ESI) *m/z*** calcd for $C_{15}H_{13}O_6N_2$ $[M+H]^+$: **317.07681**; found: **317.07626**.

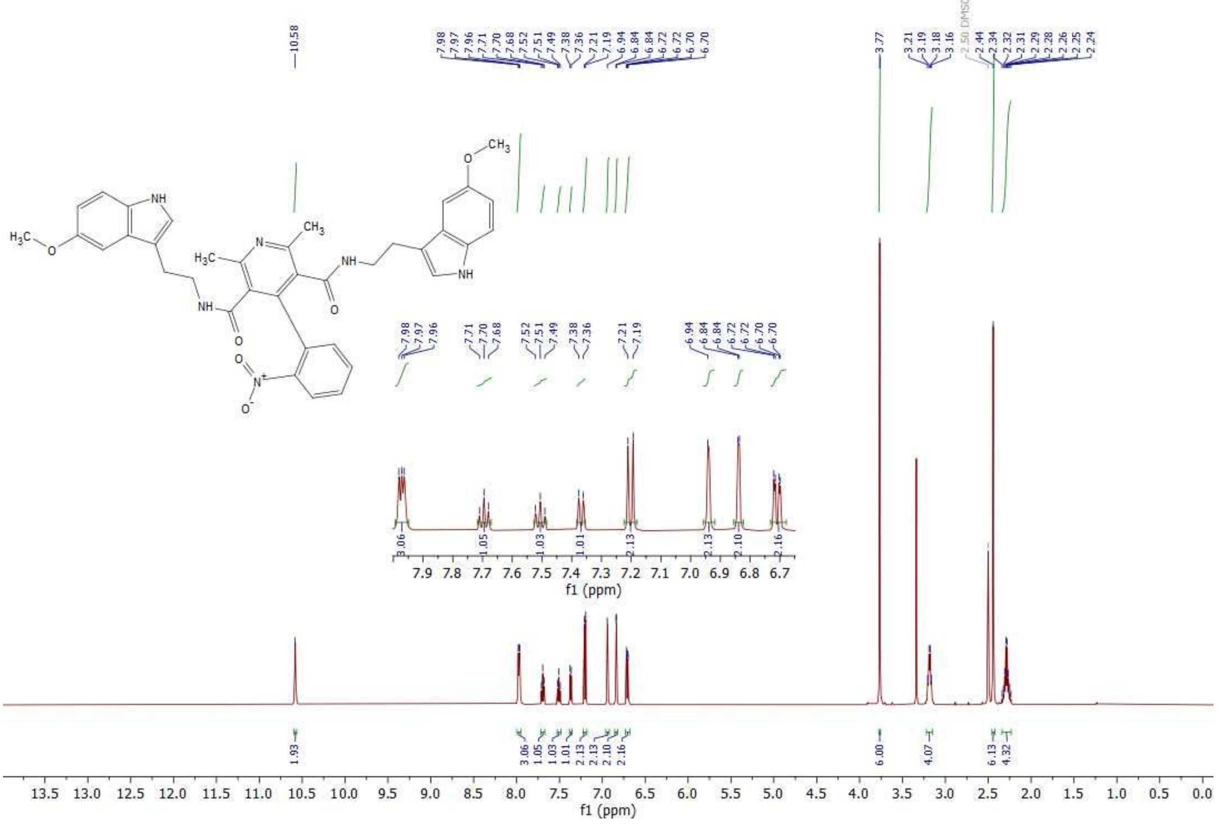

Figure S10: ^{1}H-NMR spectrum of HCM-01

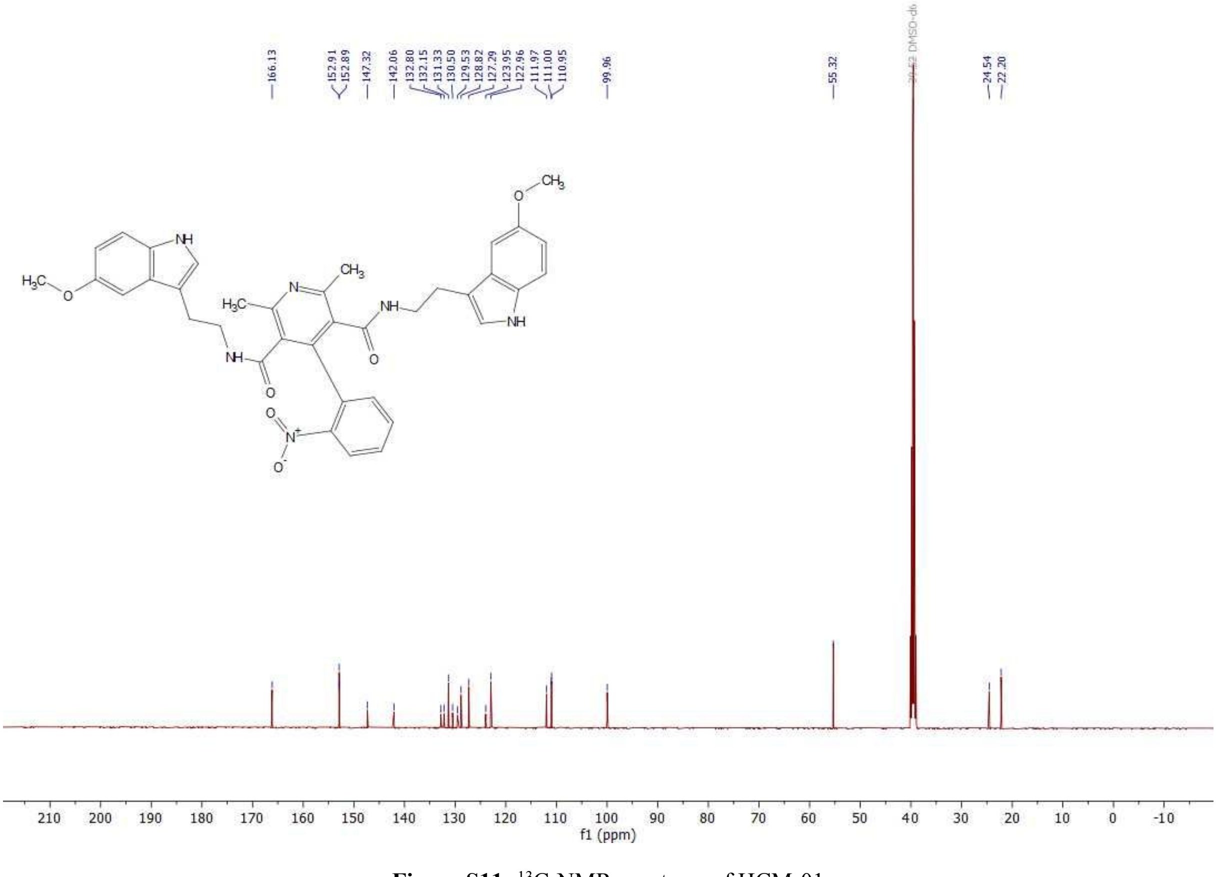

Figure S11: ^{13}C-NMR spectrum of HCM-01

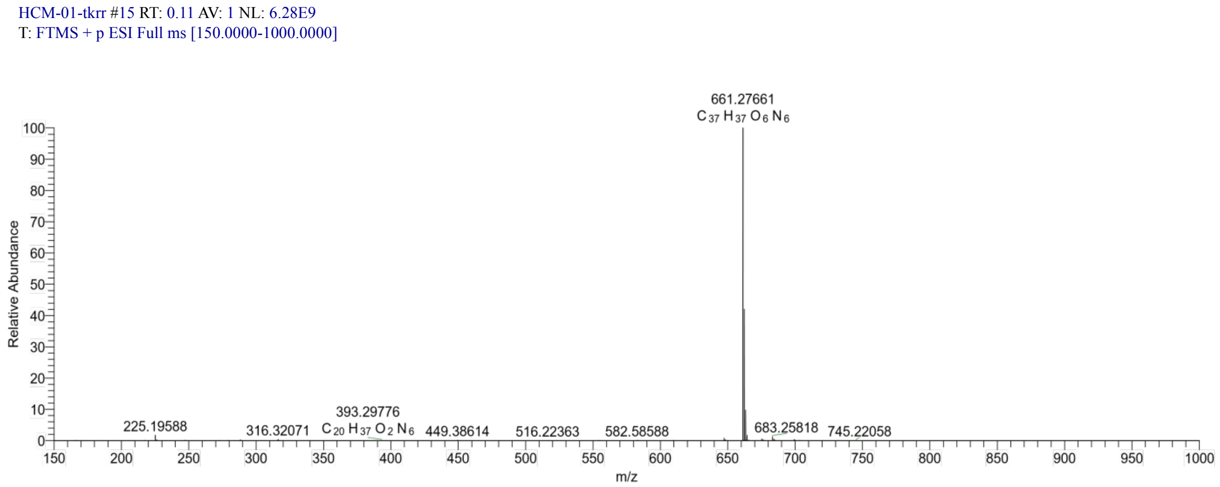

HCM-01-tkrr#15 RT: 0.11 SM: 7G
T: FTMS + p ESI Full ms [150.0000-1000.0000]

m/z	Intensity	Relative	Theo. Mass	Delta (ppm)	RDB equiv.	Composition
225.19588	109742880.0	1.75				
226.19943	15777234.0	0.25				
247.17766	11203798.0	0.18				
288.28952	18368880.0	0.29	288.28836	4.01	0.0	$C_{15}H_{36}ON_4$
316.32071	25544268.0	0.41				
449.38614	7928143.5	0.13				
647.26050	53863588.0	0.86	647.26126	-1.18	22.5	$C_{36}H_{35}O_6N_6$
648.26508	20297990.0	0.32				
661.27661	6286850048.0	100.00	661.27691	-0.45	22.5	$C_{37}H_{37}O_6N_6$
662.27942	2661171712.0	42.33				
663.28198	613622144.0	9.76				
664.28473	110185248.0	1.75				
665.29034	14474286.0	0.23				
675.29272	36980900.0	0.59				

Figure S12: HRMS spectrum of HCM-01

^{1}H NMR (500 MHz, DMSO-*d$_6$*) δ 10.58 (s, 2H), 7.99 – 7.95 (m, 3H), 7.70 (t, *J* = 7.6 Hz, 1H), 7.51 (t, *J* = 7.9 Hz, 1H), 7.37 (d, *J* = 7.4 Hz, 1H), 7.20 (d, *J* = 8.6 Hz, 2H), 6.94 (s, 2H), 6.84 (d, *J* = 2.4 Hz, 2H), 6.71 (dd, *J* = 8.7, 2.4 Hz, 2H), 3.77 (s, 6H), 3.22 – 3.15 (m, 4H), 2.44 (s, 7H), 2.35 – 2.22 (m, 5H). **^{13}C NMR (126 MHz, DMSO-*d$_6$*)** δ 166.1, 152.9, 152.9, 147.3, 142.1, 132.8, 132.1, 131.3, 130.5, 129.5, 128.8, 127.3, 123.9, 123.0, 112.0, 111.0, 111.0, 100.0, 55.3, 24.5, 22.2. **HRMS (ESI) *m/z*** calcd for $C_{37}H_{37}O_6N_6$ $[M+H]^+$: **661.27691**; found: **661.27661**.

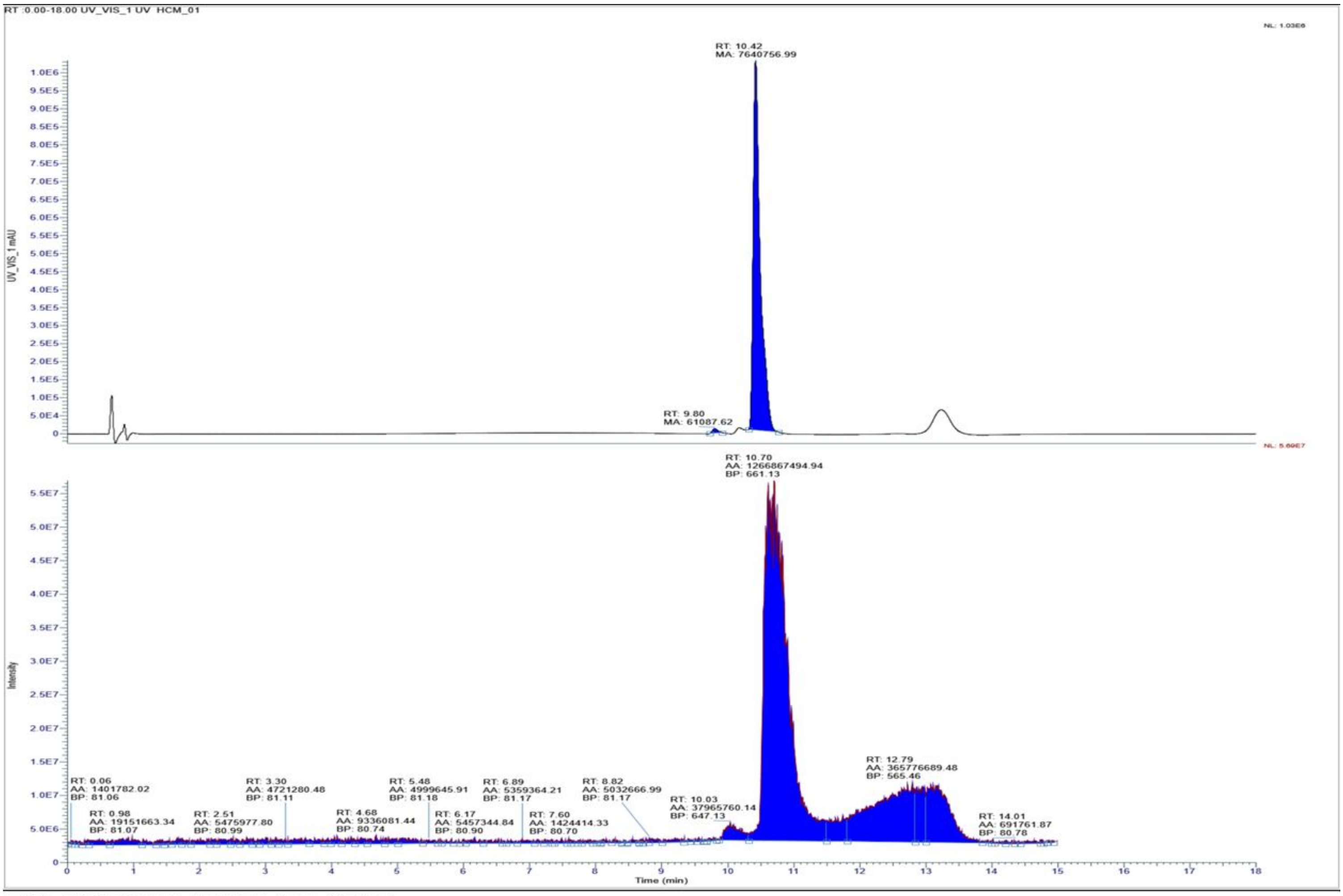

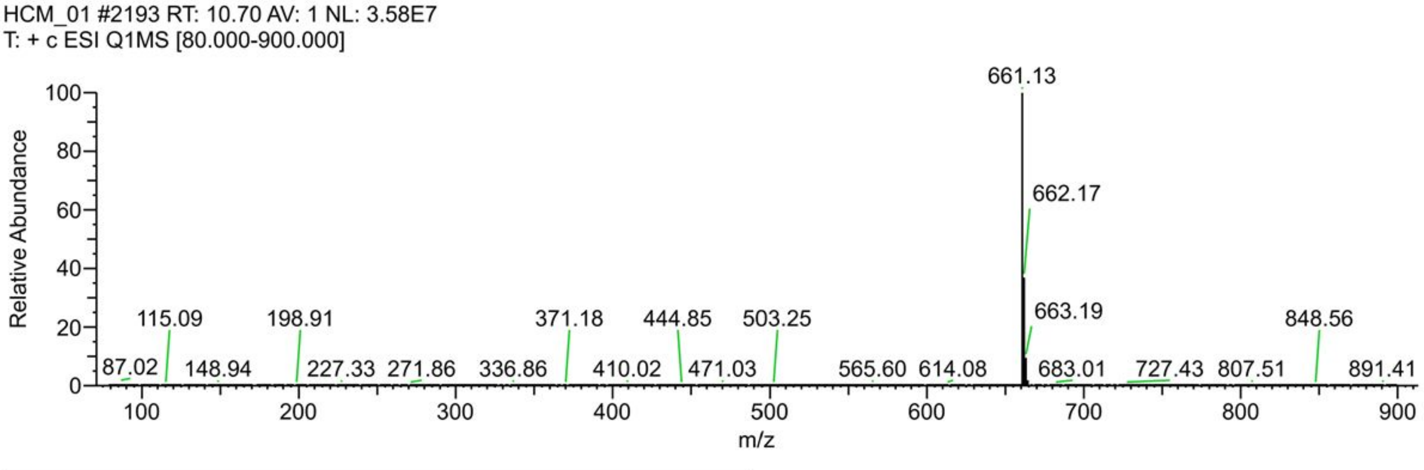

Index	RT (Min)	RT (Sec)	Start RT	End RT	Peak Area	Peak Height	% Area	% Height	Scan Filter
2	10.4234	625.404	10.3084	10.7584	7640756.9...	1022981.9...	99.21	98.86	No Filter
1	9.8034	588.204	9.7175129...	9.9123456...	61087.621...	11790.902...	0.79	1.14	No Filter

Figure S13:LCMS spectrum of HCM-01

Western Blot

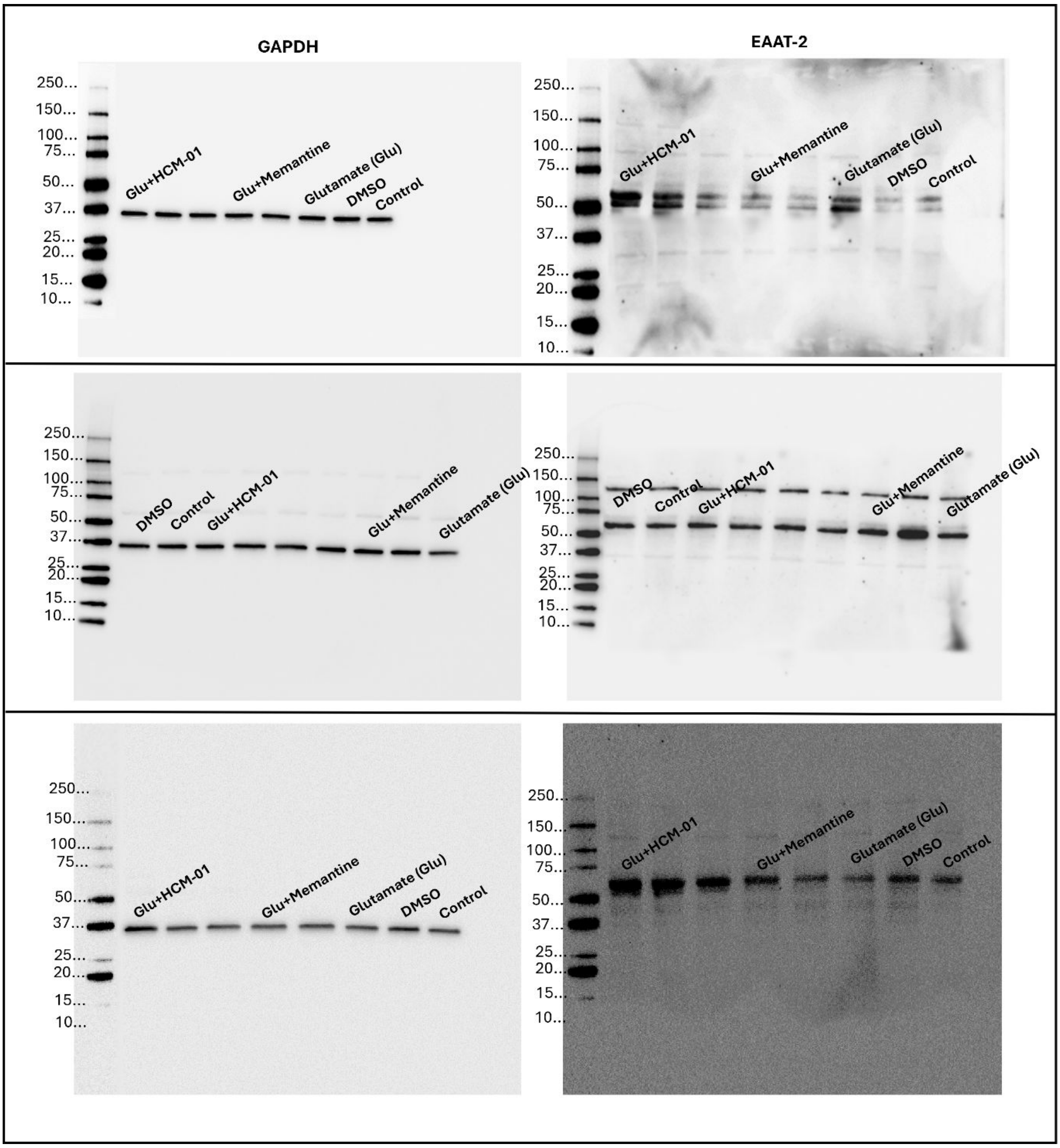

Figure S14: Western Blot immunoblot images of EAAT2 expression

Histopathological findings

Table S1. Statistical analysis of degenerative changes observed in neurons.

Groups	CA1/CA2	CA3
**Negative control**	00000000	01100000
**Sham Operation**	00000010	00010000
**STZ**	11011110	33222333

**Memantine**	00011000	11122222
**HCM-01 (Oral 50 mg/kg)**	00100000	22221122
**HCM-01 (Oral 100 mg/kg)**	00001000	22221112
**HCM-01 (I.P. 50 mg/kg)**	1000000	21121222

[a,b,c] It indicates the difference between groups in the same column ($p < 0.05$).

Immunohistochemical and immunofluorescence findings

Table S2. Immunohistochemical staining of Tau.

Groups	CA1/CA2	CA3
**Negative Control**	00111111	11121111
**Sham Operation**	00011111	12111111
**STZ**	11222222	44334444
**Memantine**	12121222	22112222
**HCM-01 (Oral 50 mg/kg)**	11121111	22222222
**HCM-01 (Oral 100 mg/kg)**	22121122	2,83±0,40[d]
**HCM-01 (I.P. 50 mg/kg)**	21111111	11212112

[a,b,c,d] It indicates the difference between groups in the same column ($p < 0.05$).

Table S3. Immunohistochemical staining with β-Amyloid**.**

Groups	CA1/CA2	CA3
**Negative Control**	00110001	00100100
**Sham Operation**	10000000	001010100
**STZ**	10000000	33332333
**Memantine**	00101100	22222111
**HCM-01 (Oral 50 mg/kg)**	01100100	00100100
**HCM-01 (Oral 100 mg/kg)**	00100011	10100100
**HCM-01 (I.P. 50 mg/kg)**	00100100	00100001

[a,b,c,d] It indicates the difference between groups in the same column ($p < 0.05$).

Table S4. Immunohistochemical staining with AChE.

Groups	CA1/CA2	CA3
**Negative Control**	00100100	11111111
**Sham Operation**	00100100	11111111
**STZ**	00100100	43344444

**Memantine**	00100100	33223333
**HCM-01 (Oral 50 mg/kg)**	10100001	22222232
**HCM-01 (Oral 100 mg/kg)**	00100100	10110000
**HCM-01 (I.P. 50 mg/kg)**	00100100	01000000

[a,b,c,d] It indicates the difference between groups in the same column ($p < 0.05$).
